# Engineered Binding Microenvironments in Halogen Bonding Polymers for Enhanced Anion Sensing

**DOI:** 10.1002/anie.202300867

**Published:** 2023-02-23

**Authors:** Krzysztof M. Bąk, Sophie C. Patrick, Xiaoxiong Li, Paul D. Beer, Jason J. Davis

**Affiliations:** ^1^ Department of Chemistry University of Oxford South Parks Road Oxford OX1 3QZ UK

**Keywords:** Anions, Electrochemistry, Host–Guest Systems, Polymers, Solvent Effects

## Abstract

Mimicking Nature's polymeric protein architectures by designing hosts with binding cavities screened from bulk solvent is a promising approach to achieving anion recognition in competitive media. Accomplishing this, however, can be synthetically demanding. Herein we present a synthetically tractable approach, by directly incorporating potent supramolecular anion‐receptive motifs into a polymeric scaffold, tuneable through a judicious selection of the co‐monomer. A comprehensive analysis of anion recognition and sensing is demonstrated with redox‐active, halogen bonding polymeric hosts. Notably, the polymeric hosts consistently outperform their monomeric analogues, with especially large halide binding enhancements of ca. 50‐fold observed in aqueous‐organic solvent mixtures. These binding enhancements are rationalised by the generation and presentation of low dielectric constant binding microenvironments from which there is appreciable solvent exclusion.

## Graphical abstract

Halogen‐bonding (XB), redox‐active copolymeric hosts are demonstrated to enhance anion binding and sensing performance with respect to their discrete receptive monomers. Through judicious choice of co‐monomers, the local dielectric environment within the polymeric hosts can be modulated, resulting in up to a 50‐fold improvement in binding constant with a XB polymeric host with I^−^ compared to its monomer.

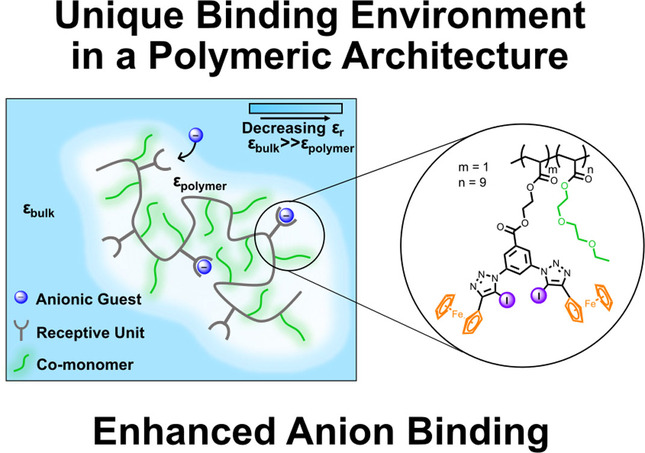

## Introduction

Anions play vital roles in many biological and industrial processes, necessitating their selective binding within a wide range of applications such as water quality monitoring, ion extraction and catalysis.^[1]^ A key challenge in the design of anion‐receptive host systems is overcoming competitive anion solvation.^[2]^ To date, the vast majority of reported abiotic anion receptors operate in low‐polarity organic solvents, where the energetic penalty of ion de‐solvation is low and the electrostatic components of non‐covalent interactions between the host and guest are strong.^[3]^ Recent reports have established a direct relationship between solvent dielectric (ϵ_r_) and anion binding affinity, further underlying the formidable challenge of anion recognition in more polar protic environments.[^2a^, ^4^] Nevertheless, receptors capable of strong and selective anion binding in water are ubiquitous in nature, with examples of anion receptive proteins for targets such as sulfate or phosphate.^[5]^ These biopolymeric hosts circumvent high de‐solvation penalties through the generation of shielded cavities buried within the protein scaffold, where the relative permittivity is lower than that of the surrounding bulk environment. Thus, the strength of hydrogen bonds, and other electrostatic interactions that commonly contribute to biotic anion binding, is promoted.^[6]^ Such natural receptors serve as a source of inspiration for host designs that exploit preorganised microenvironments to enable anion recognition in highly polar media; to date these include clam‐shell bicyclopeptides,^[7]^ mechanically interlocked structures,^[8]^ molecular cages,^[9]^ cavitands,^[10]^ and foldamers.[^4a^, ^11^] It has additionally been noted that the confinement of Lewis acidic receptors within two dimensional molecular films often enhances anion binding; again, this has been attributed to generated low dielectric environments and associated solvent exclusion effects.^[12]^

Although the aforementioned abiotic synthetic approaches have enabled anion binding to be achieved in polar media, the synthesis and potential scaling‐up of these, e.g. for sensing applications, is profoundly challenging.^[13]^ Polymer architectures offer a chemically rich and synthetically tractable alternative to the complex molecular design of discrete molecular systems. The ease by which a polymer's properties can be tuned by the strategic selection of monomers has facilitated the generation of scalable and robust sensors for a large variety of targets.^[14]^ The use of polymers for anion recognition, however, remains largely undeveloped. Recently, (co)polymers containing well‐established hydrogen bonding (HB) anion recognition motifs, such as calix[4]pyrroles,^[15]^ triazoles,^[16]^ ureas,^[17]^ and squaramides^[18]^ have been described, but their binding properties across different solvents have not been studied in detail. The effect on anion binding performance when these motifs are introduced into polymeric architectures remains largely unexplored. Interestingly, some polymeric hosts have been shown to extract anions from water,[^1c^, ^19^] and bind weakly hydrated anions such as SCN^−^ or I^−^ even in the absence of specific anion receptive units.^[20]^ These are clear indications that polymeric hosts can have significant and beneficial effects on anion binding over their constituent monomeric receptors.

Whilst the vast majority of artificial anion receptors have been based on HB motifs, in recent years sigma‐hole interactions such as halogen bonding (XB) have emerged as a compelling alternative. In comparison to HB, these interactions are characterised by more stringent directionality, with a larger charge‐transfer contribution to binding, and are generally less sensitive to solvent and pH.^[21]^ As a result, XB hosts frequently outperform their HB analogues in competitive polar solvents including water, and often display unique selectivity trends.[^12b^, ^21^, ^22^] The XB interaction strength can be tuned with electron‐withdrawing groups or appended redox switches (e.g. ferrocene, Fc), giving rise to highly functional anion‐binding sites.[^12a^, ^23^] Furthermore, the hydrophobic character of XB‐based binding motifs makes them exceptionally promising candidates to incorporate into polymeric scaffolds in the formation of binding cavities of reduced polarity. XB polymers are only beginning to emerge as a fascinating class of new materials and remain largely unexplored in the context of anion recognition.^[24]^

Herein, we present engineered, redox‐active, XB polymeric host systems, which demonstrate significantly enhanced anion binding strengths (up to 47‐fold) relative to their monomeric receptor analogues. Systematic comparisons of polymer and monomer binding were conducted by complementary ^1^H NMR and electrochemical voltammetric methods in a range of solvent media, to elucidate both the impact of a polymeric scaffold on anion binding performance, and the solvation effects which govern these phenomena. We further demonstrate that a judicious selection of co‐monomer, and thus modulation of binding microenvironment, has a profound and beneficial impact on anion binding strength. We envisage this approach to be applicable to a range of hosts and targets, enabling recognition and sensing in highly polar media, a capability unattainable with the monomeric binding units alone.

## Results and Discussion

### Polymeric receptor design and synthesis

**Figure 1:**
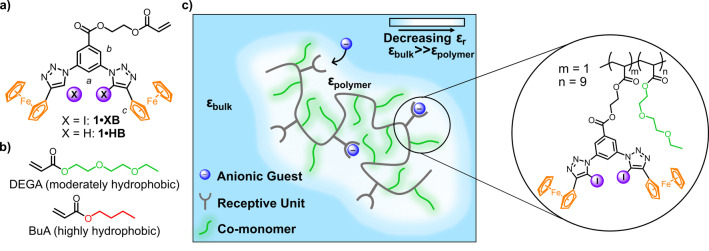
a) Monomeric receptors **1⋅XB/HB**. b) Acrylates selected as co‐monomers for synthesis of polymeric hosts: DEGA and BuA. c) Cartoon representation of polymeric anion binding host **pDEGA‐1⋅XB** obtained by a statistical RAFT polymerisation. Local solvation and dielectric properties around the receptive units can be controlled to generate a microenvironment (ϵ_polymer_) where anion binding is markedly enhanced over that in bulk solvent (ϵ_bulk_).

Building upon our previous work with XB receptors, we incorporated 3,5‐bis‐(iodotriazole)benzene XB donors as the anion binding motifs in the acrylate monomer **1⋅XB** (Figure 1a).^[25]^ The electron withdrawing ester group at position 1 of the aromatic ring was chosen to increase the potency of the halogen bond donor sites. Ferrocene units were directly appended to the triazole rings to act as redox‐active molecular sensing probes and electrochemically modulate the strength of the XB interactions.[^23c^, ^26^] Target monomer **1⋅XB**, and its HB analogue **1⋅HB**, were prepared via a robust copper(I)‐catalysed azide‐alkyne cycloaddition (CuAAC) reaction between two equivalents of (iodo)ethynylferrocene and 3,5‐diazidobenzoate containing a polymerisable acrylate unit (Supporting Information, Section S2.1).

A statistical co‐polymerisation of monomers **1⋅XB/1⋅HB** and non‐receptive co‐monomers (see Figure 1a) was achieved by a reversible addition‐fragmentation chain transfer (RAFT) polymerisation in the presence of AIBN as the thermal initiator and 2‐(dodecylthiocarbonothioylthio)‐2‐methylpropanoic acid (DDMAT) as the RAFT agent (Supporting Information, Section S2.2).^[27]^ In a typical procedure, monomer **1⋅XB** was mixed with a co‐monomer (Figure 1b) in a 1 : 9 ratio for at least 24 h at 70 °C in DMF (molar ratio of 200 : 1 : 0.2 [M_total_]:[DDMAT]:[AIBN]) followed by purification by size exclusion chromatography to obtain final polymers in yields ranging from 30 % to 65 % (Figure 1c). Reaction progress was monitored by a gradual reduction of the intensity of the characteristic acrylate ^1^H NMR signals (δ=5.5–6.5 ppm), and the formation of a new set of broad signals typical for polymers (Supporting Information, Figures S2.10‐S2.19). The incorporation of the monomers into the polymer backbone was further confirmed by the loss of the characteristic acrylate C=C stretch band at 1610 cm^−1^ in ATR‐IR spectra (Supporting Information, Figures S3.1 & S3.2). *M*_n_ (number average molecular weight) and PDI (polydispersity index) were resolved by gel permeation chromatography (GPC, see Supporting Information Figures S3.3 & S3.4, Table S3.1). The average composition of the polymers was estimated from ^1^H NMR integration and *M*_n_. Full synthetic procedures and compound characterisation data are provided in the Supporting Information, Sections S2 and S3.

Di(ethylene glycol) ethyl ether acrylate (DEGA) was chosen as one of the model co‐monomers because it ensured good solubility of the resulting polymeric hosts across a range of chosen solvents. It also has a sufficiently low permittivity in comparison to the chosen solvents to potentially induce local dielectric effects.^[28]^ n‐Butyl acrylate (BuA) was selected as a distinctly more hydrophobic monomer and was incorporated into a soluble polymer as a mixture with DEGA (1 : 1 molar ratio).^[29]^ For further analysis we selected four distinct co‐polymers (m:n:o, the statistical composition where *m*=**1⋅XB**, *n*=DEGA, and o=BuA):

- **pDEGA‐1⋅XB** (*m*:*n*=1 : 9, *M*_n_=15.7 kDa, PDI=1.4),
- **pDEGA‐1⋅HB** (*m*:*n*=1 : 9, *M*_n_=28.1 kDa, PDI=1.4)—a hydrogen bonding analogue,
- **pDEGA‐1⋅XB_0.5_** (*m*:*n*=5 : 95, *M*_n_=12.2 kDa, PDI=1.4)—an analogue with a lower ratio of the XB motif to DEGA co‐monomer, and
- **pDEGA‐BuA‐XB** (*m*:*n*:*o*=10 : 45 : 45, *M*_n_=16.7 kDa, PDI=1.4)—an analogue containing a non‐polar BuA monomer.

### ^1^H NMR anion binding studies of 1⋅XB/HB

The anion binding properties of **1⋅XB** were investigated by ^1^H NMR titrations in three organic‐aqueous solvent systems of increasing polarity: 97.5 : 2.5 acetone‐d_6_/D_2_O, 48.75 : 48.75 : 2.5 acetone‐d_6_/ACN‐d_3_/D_2_O and 97.5 : 2.5 ACN‐d_3_/D_2_O. The choice of solvent media, spanning dielectric constants from ϵ_r_=26 to ϵ_r_=39 (determined as molar linear combinations of reported ϵ_r_ values of the pure solvents), allowed a detailed investigation into the impact of solvent polarity on complexation (Supporting Information, Section S4).

**Figure 2:**
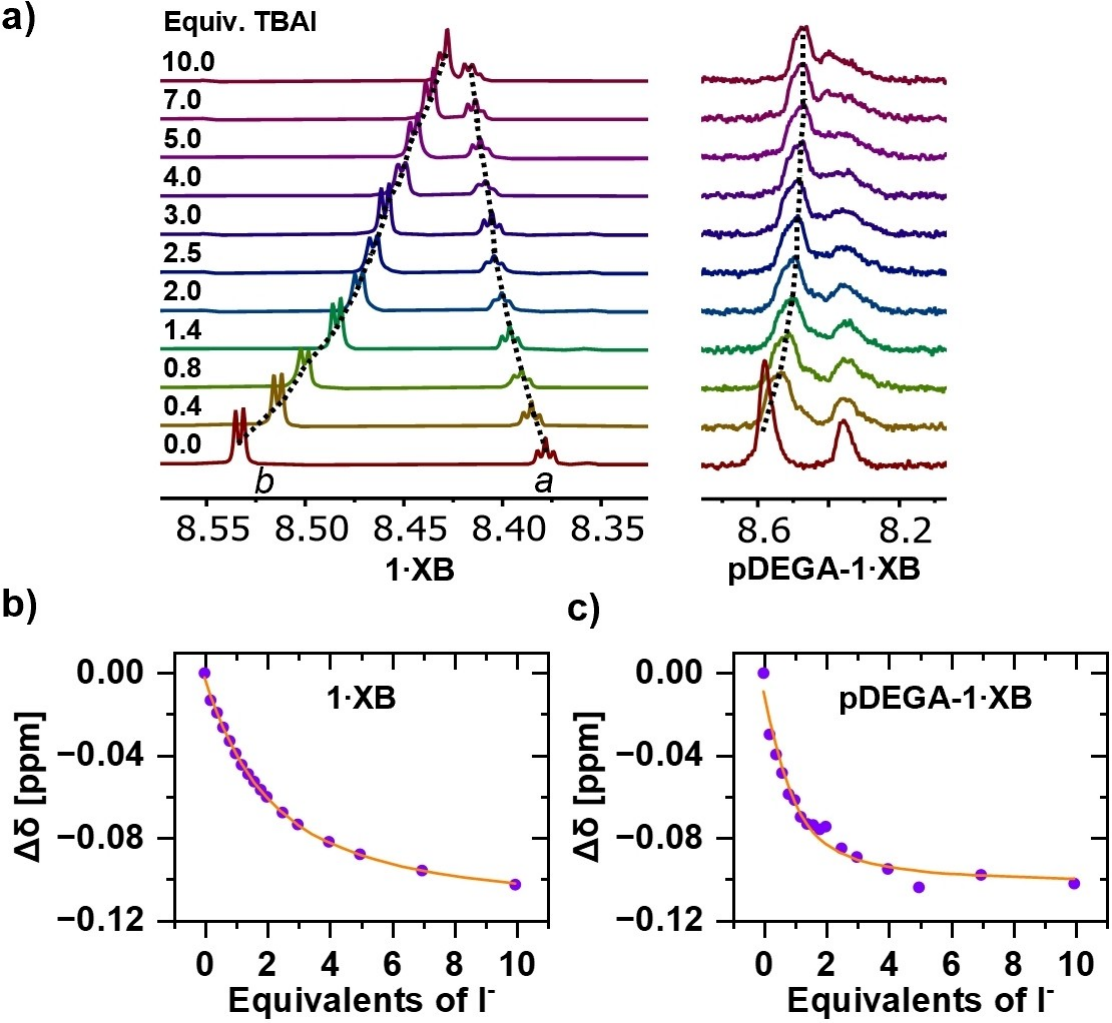
a) Truncated ^1^H NMR spectra of **1⋅XB** and **pDEGA‐1⋅XB** upon titration with TBAI in 97.5 : 2.5 acetone‐d_6_/D_2_O. Corresponding I^−^ binding isotherms of b) **1⋅XB** and c) **pDEGA‐1⋅XB**.

The addition of tetrabutylammonium salts (TBAX) of various anions (X=I^−^, Br^−^, Cl^−^ or H_2_PO_4_^−^) to a solution of **1⋅XB** in 97.5 : 2.5 acetone‐d_6_/D_2_O induced a significant downfield shift of the receptor's central aryl proton *a* and an upfield shift of protons *b* and *c* in all cases, confirming that the anion binding event occurred in the cleft formed by the central aryl ring and iodotriazole moieties (Figure 2a). Bindfit analyses of the binding isotherms (for the most perturbed protons *a & b*) revealed 1 : 1 host–guest stoichiometry with corresponding association constants shown in Table 1.^[30]^ The observed anion binding selectivity trend follows the Hofmeister bias of anion hydration enthalpies, where the large, lipophilic, and polarisable iodide forms the most stable complex with receptor **1⋅XB**: I^−^>Br^−^>Cl^−^>H_2_PO_4_^−^ (Table 1). It should be noted that the marked differences in anion binding with the XB and HB receptors (see below) suggest that recognition is dominated by specific XB/HB interactions, and is not just a simple Hofmeister‐biased recruitment.

**Table 1:** Anion association constants *K*_a_ [M^−1^] of monomeric receptor **1⋅XB** as determined by ^1^H NMR titrations in 97.5 : 2.5 acetone‐d_6_/D_2_O, 48.75 : 48.75 : 2.5 acetone‐d_6_/ACN‐d_3_/D_2_O and 97.5 : 2.5 ACN‐d_3_/D_2_O.

**Anion**	97.5 : 2.5 acetone‐d_6_/D_2_O^[a]^	48.75 : 48.75 : 2.5 acetone‐d_6_/ACN‐d_3_/D_2_O^[a]^	97.5 : 2.5 ACN‐d_3_/D_2_O^[a]^
Cl^−^	170	40	20
Br^−^	470	130	60
I^−^	640	220	110
H_2_PO_4_^−^	60	25	<10

[a] Standard errors estimated to be 10 %.

Notably, **1⋅XB** significantly outperformed its HB analogue, **1⋅HB**, (Tables S4.1 & S4.2) with over a 12‐fold increase of the I^−^ binding constant value observed for **1⋅XB** (*K*_a_=640 M^−1^) in comparison to **1⋅HB** (*K*_a_=50 M^−1^), highlighting the superior binding properties of σ‐hole donors over HB motifs and underlining the halogen bonding preference for softer and more lipophilic anions in polar media.

Analogous ^1^H NMR anion titration experiments were undertaken in more competitive organic‐aqueous solvent mixtures (48.75 : 48.75 : 2.5 acetone‐d_6_/ACN‐d_3_/D_2_O and 97.5 : 2.5 ACN‐d_3_/D_2_O). As expected, binding affinities were observed to diminish (Table 1), particularly for highly solvated anions: Cl^−^ and H_2_PO_4_^−^.

### ^1^H NMR anion binding studies of pDEGA‐1⋅XB

Following an assessment of the anion binding performance of the **1⋅XB** monomer motif, ^1^H NMR titrations of the polymeric hosts were conducted in the same organic‐aqueous solvent systems at the same effective binding unit concentration as in the titrations of monomers to ensure strictly comparable conditions (Supporting Information, Section S5). The effective concentrations were calculated using the results of *M*_n_ analysis by GPC and the molar ratios (*m*:*n*:*o*) as determined by ^1^H NMR. For example, **pDEGA‐1⋅XB** host contained on average 5.7 receptive units per *M*_n_=15.7 kDa polymer strand, thus concentrations of **pDEGA‐1⋅XB** were normalised by a factor of 5.7. A comparative integration of characteristic ^1^H NMR signals from the polymeric host and TBA salts throughout the titrations confirmed that the correct equivalents of anionic guest were added. An analysis of the errors associated with this method is included in Supporting Information, Section S6.1 (confirming the potential impact of error in determining binding unit concentration is near identical for monomers and polymers, i.e. there is no additional uncertainty in the binding unit concentration of the polymeric hosts compared to the monomeric hosts. The proton signals of **pDEGA‐1⋅XB** were significantly broader than those of monomeric **1⋅XB** (Figure 2a), as is expected for a high molecular weight macromolecule whose repeating units are statistically distributed through marginally different chemical environments. A detailed analysis is included in Supporting Information, Section S6.2, Table S6.2 confirming that this does not introduce a significant error (<10 %) in the resolved binding constants.^[31]^ Importantly, the polymer host's proton signal perturbations caused by the addition of TBA salts can, then, be monitored and used to determine anion binding constants (Figure 2c).

The addition of TBA salts to a solution of **pDEGA‐1⋅XB** in 97.5 : 2.5 acetone‐d_6_/D_2_O induced shifts of protons *a*, *b*, and *c* that resembled changes observed during titrations of the **1⋅XB** monomer (Figure 2a). Notably, no additional changes of the DEGA co‐monomer and polymer backbone signals were observed, confirming that anion recognition was localised in the XB cleft of the binding motif (as with monomeric **1⋅XB**). A quantitative analysis of the binding isotherms revealed a 1 : 1 stoichiometric binding unit‐guest association, confirming that each chelating bidentate XB motif binds anions independently within the polymer architecture.^[32]^

The consistency of the 1 : 1 host–guest binding mode enabled a direct comparison of the polymer host binding properties with monomer **1⋅XB**. Significantly, the receptor unit incorporated in the polymer binds anions ca. 6 times stronger than **1⋅XB** (Table 2), while the binding selectivity trend remains practically unaltered : I^−^ (*K*_a_=3000 M^−1^)>Br^−^ (*K*_a_=2800 M^−1^)>Cl^−^ (*K*_a_=1100 M^−1^)>H_2_PO_4_^−^ (*K*_a_=410 M^−1^). The magnitude of binding constants is particularly impressive considering the electrostatic neutrality of the polymeric host. Interestingly, this polymer‐based binding enhancement was also observed in the case of the HB analogue **pDEGA‐1⋅HB**, but was weaker in comparison to **pDEGA‐1⋅XB**, with an average 2‐fold increase of halide association constants from the analogous monomers (Supporting Information, Tables S5.2 & S5.3).This difference in polymer potency may originate from discrepancies in binding site solvation due to stronger interactions between the HB binding motif and solvent molecules or even with DEGA co‐monomers (supported by comparison of the resolved *E*_1/2_ values of the XB vs. HB analogues in different solvent media, see below). Importantly, these observations highlight the potential of XB donors in the construction of highly effective hosts.

**Table 2:** Anion association constants *K*_a_ [M^−1^] of **pDEGA‐1⋅XB** and the polymeric enhancement in binding constant (**K_pDEGA‐1⋅XB_/K_1⋅XB_**), as determined by ^1^H NMR titrations in competitive polar media.

	97.5 : 2.5 acetone‐d_6_/D_2_O		48.75 : 48.75 : 2.5 acetone‐d_6_/ACN‐d_3_/D_2_O		97.5 : 2.5 ACN‐d_3_/D_2_O	
Anion	**pDEGA‐1⋅XB** ^[a]^	K_**pDEGA‐1⋅XB**_/K_**1⋅XB**_^[b]^	**pDEGA‐1⋅XB** ^[a]^	K_**pDEGA‐1⋅XB**_/K_**1⋅XB**_^[b]^	**pDEGA‐1⋅XB** ^[a]^	K_**pDEGA‐1⋅XB**_/K_**1⋅XB**_^[b]^
Cl^−^	1100	6.5	320	8.0	240	12.0
Br^−^	2800	6.0	1300	10.0	880	14.7
I^−^	3000	4.7	1800	8.2	1650	15.0
H_2_PO_4_^−^	410	5.8	240	9.6	80	>8.0

[a] Standard errors estimated to be <20 %. [b] Standard errors estimated to be 22.5 %. Error approximation is detailed in Supporting Information, Section S6.

**Figure 3:**
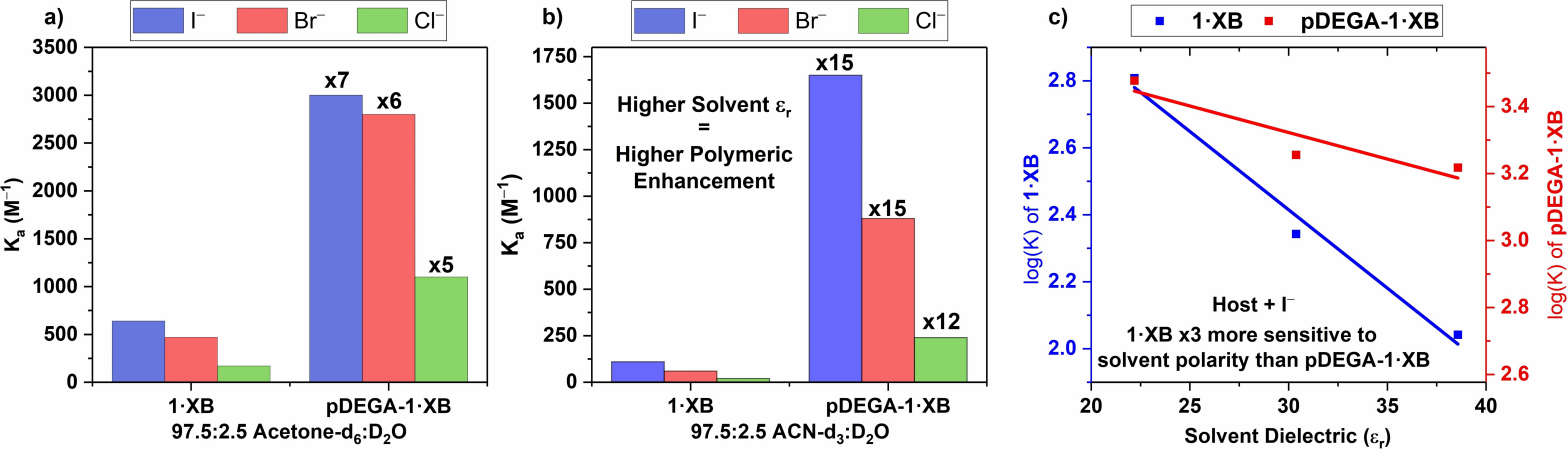
Comparison of halide binding constants with **1⋅XB** and **pDEGA‐1⋅XB** in a) 97.5 : 2.5 acetone‐d_6_/D_2_O and b) 97.5 : 2.5 ACN‐d_3_/D_2_O. c) Comparison of the log(*K*_a_) vs. solvent dielectric constant for binding constants with **1⋅XB** and **pDEGA‐1⋅XB** in response to I^−^.

The anion binding properties of **pDEGA‐1⋅XB** were then investigated further in the more competitive organic‐aqueous solvent mixtures: 48.75 : 48.75 : 2.5 acetone‐d_6_/ACN‐d_3_/D_2_O and 97.5 : 2.5 ACN‐d_3_/D_2_O. As expected, a decrease of association constant values was observed, however the relative suppression of binding was considerably less significant for **pDEGA‐1⋅XB** than **1⋅XB** (Table 2). **pDEGA‐1⋅XB** clearly outperforms the monomer as solvent polarity rises (there is a progressively higher “polymer enhancement”, Figure 3), with halide binding constants being ca. 10‐fold and up to 15‐fold higher than those of **1⋅XB** in 48.75 : 48.75 : 2.5 acetone‐d_6_/ACN‐d_3_/D_2_O and 97.5 : 2.5 ACN‐d_3_/D_2_O respectively. Notably, increasing the polarity of the solvent only has a modest effect on halide binding by **pDEGA‐1⋅XB**. Switching from the 97.5 : 2.5 acetone‐d_6_/D_2_O (ϵ_r_=26) solvent system to 48.75 : 48.75 : 2.5 acetone‐d_6_/ACN‐d_3_/D_2_O (ϵ_r_=33.5) resulted in ca. 40 % decrease of I^−^ and Br^−^ binding constant values, while for **1⋅XB** the observed decrease is ca. 70 % (Figure 3).

To confirm that the specific integration of the bidentate XB binding sites within the polymeric structural framework was responsible for the observed enhanced anion recognition effects, a control ^1^H NMR titration of **1⋅XB** monomer with I^−^ was conducted in presence of a non‐receptive pDEGA homopolymer, ensuring the 1 : 9 molar statistical distribution of **1⋅XB** to DEGA as in **pDEGA‐1⋅XB**. Bindfit analysis determined an almost identical association constant (*K*_a_=110 M^−1^) to that of **1⋅XB** alone (*K*_a_=105 M^−1^) in 97.5 : 2.5 ACN‐d_3_/D_2_O. As expected, none of the signals associated with pDEGA shifted significantly (Δδ<0.05 ppm) confirming that the DEGA side chains themselves do not directly contribute to anion binding. Analysis of the TBA^+^ signals during titrations, and a control NaI titration, confirmed that TBA^+^ does not interact/incorporate significantly with the polymeric hosts or contribute to binding enhancements in any way (see Supporting Information, Section S7 for further details).

The notably enhanced anion recognition properties of polymeric host **pDEGA‐1⋅XB** in comparison to monomer **1⋅XB** across different organic‐aqueous solvent mixtures, is indicative of the unique local microenvironments within the polymeric host scaffold, where partial exclusion of competing polar solvent molecules and the inherently lower relative dielectric constant of the DEGA co‐monomer serve to augment anion binding potency. As noted, this is further supported by comparison of the characteristic half‐wave potentials which are indicative of the polymer host receptive units being in a less polar environment (*E*_1/2,poly_>*E*_1/2,mon_, discussed in detail below).

We sought to further modulate the binding site local microenvironment, initially by increasing the dilution of the XB motif with the DEGA co‐monomer (**pDEGA‐1⋅XB_0.5_**). Proton NMR binding studies revealed weaker I^−^ binding than **pDEGA‐1⋅XB** (*K*_a_=570 M^−1^ vs. 1650 M^−1^, respectively in 97.5 : 2.5 ACN‐d_3_/D_2_O). These observations are consistent with a higher local concentration of more polar and flexible DEGA units around each XB unit increasing both local solvation and dielectric, and reducing anion binding affinity.

**Figure 4:**
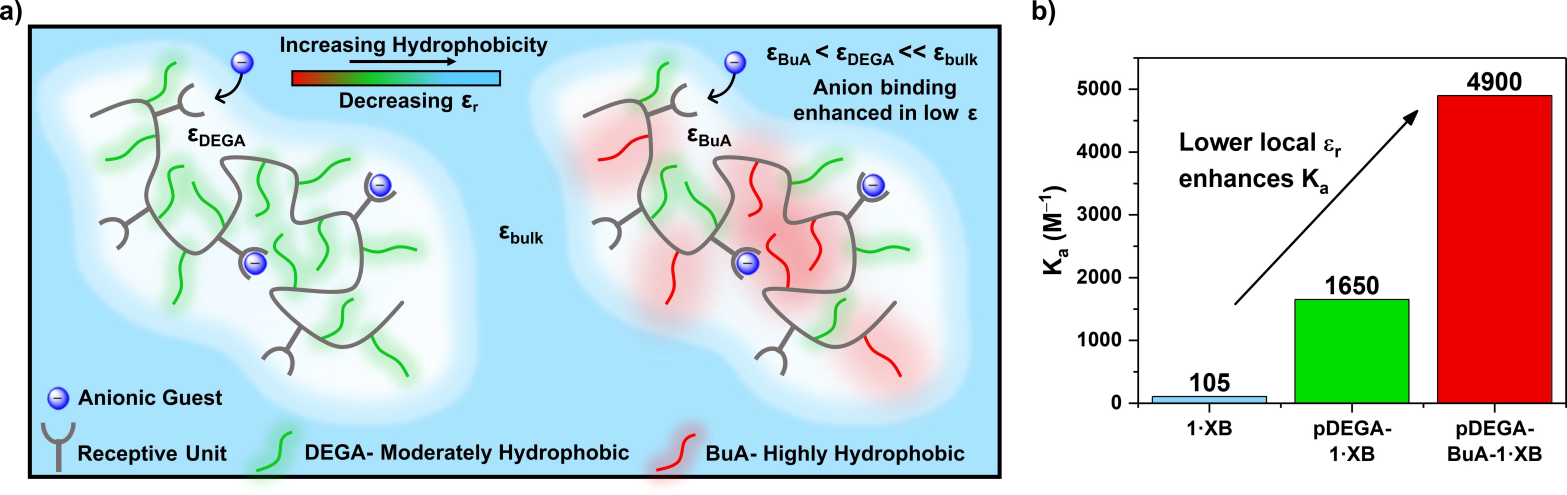
a) Schematic representation of binding microenvironment tuning by the strategic use of more hydrophobic co‐monomers. b) Enhancement of I^−^ binding constants with a series of hosts: **1⋅XB**, **pDEGA‐1⋅XB** and **pDEGA‐BuA‐1⋅XB**, as determined from ^1^H NMR titrations in 97.2 : 2.5 ACN‐d_3_/D_2_O.

The anion binding performance of **pDEGA‐BuA‐1⋅XB**, a polymeric analogue containing a non‐polar butyl acrylate co‐monomer was then investigated. It was envisioned that the incorporation of such a moiety into the polymer would result in further exclusion of solvent from the XB binding pocket, a lowering of the local dielectric, and a stronger anion binding (Figure 4a). Indeed a ^1^H NMR titration experiment with the strongest bound I^−^ in 97.5 : 2.5 ACN‐d_3_/D_2_O revealed a 47‐fold increase of the association constant in comparison to **1⋅XB** (**pDEGA‐BuA‐1⋅XB** K_a_ for I^−^=4900 M^−1^, Figure 4b). This clearly shows that modulation of the hydrophobicity of the surrounding co‐monomers can directly impact and tune the anion binding properties of polymeric hosts.

### Electrochemical characterisation of monomeric and polymeric hosts

**Figure 5:**
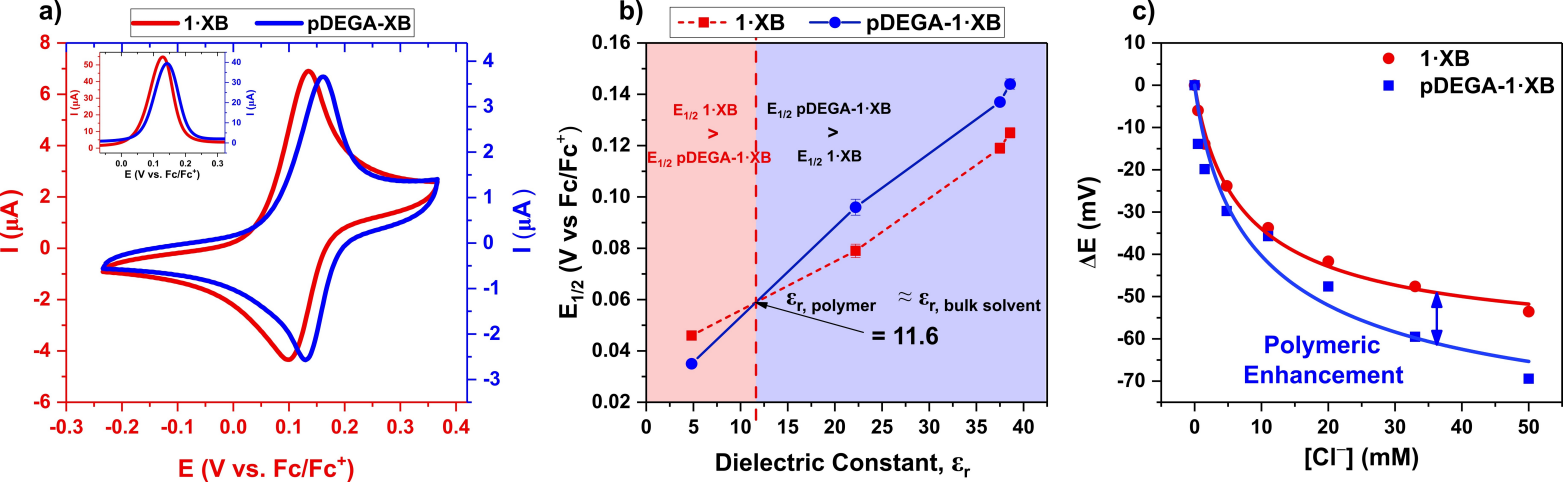
a) Cyclic voltammograms and square wave voltammograms (inset) of 0.1 mM **1⋅XB** (red) and **pDEGA‐1⋅XB** (blue) in 97.5 : 2.5 ACN/H_2_O, with 100 mM TBAClO_4_ as supporting electrolyte. b) Half wave potentials of **pDEGA‐1⋅XB** and **1⋅XB** determined in a range of solvent media of differing polarity. Connecting lines are shown to guide the eye only. Error bars represent one standard deviation of three independent repeats. All potentials wrt. Fc|Fc^+^. We hypothesise that the labelled crossover point at ≈12 represents the approximate dielectric constant within **pDEGA‐1⋅XB**. c) Binding isotherms for **1⋅XB** and **pDEGA‐1⋅XB** in 97.5 : 2.5 ACN/H_2_O with 100 mM TBAClO_4_ as supporting electrolyte, demonstrating the enhanced binding responses of the polymeric system as observed in response to Cl^−^.

The electrochemical characteristics and anion sensing performance of the monomeric and polymeric hosts were assessed by addressing the ferrocene units directly appended to the bidentate XB iodo‐triazole heterocycle motif (Supporting Information, Section S8). Ferrocene serves as an excellent electrochemical probe for transducing anion binding and as a switch increasing XB potency upon in situ oxidation.[^12a^, ^33^] Cyclic and square wave voltammetry (CV & SWV) revealed well‐defined voltammetric traces of all monomeric and polymeric hosts in solution, with single‐electron behaviour attributable to the Fc|Fc^+^ redox couples, as representatively shown by voltammograms of **1⋅XB** and **pDEGA‐1⋅XB** in 97.2 : 2.5 ACN/H_2_O (Figure 5a, potentials are reported vs. a Fc|Fc^+^ external standard). Varying the scan rate during CV measurements confirmed a quasi‐reversibility of the Fc|Fc^+^ redox couple, and any slight deviations from ideal peak current vs scan rate trends were attributed to some degree of physisorption (to a greater extent with the polymeric hosts; see graph D of Figures S8.6 and S8.9–S8.12) to the glassy carbon (GC) electrode surface. To counter this, mechanical polishing of the GC electrode was performed before every measurement. The observation of a single redox peak for all hosts indicated that both Fc transducers of each individual binding unit/monomer were addressed simultaneously. Given that the half‐wave potential (*E*_1/2_) of each redox‐active host represents the relative stabilisation of the Fc|Fc^+^ oxidation states, in turn modulated by both through‐bond (see Supporting Information, Section S8 for further details) and through‐space effects, these characteristic *E*_1/2_ values will directly report on the local environment within each polymeric host. Thus, a lower local dielectric constant would be expected to hinder oxidation and be associated with a corresponding anodic shift of half‐wave potential. As predicted, the *E*_1/2_ of **pDEGA‐1⋅XB** was observed to be 19 mV more anodic than that of **1⋅XB** (144±2 mV vs. 125±2 mV in 97.5 : 2.5 ACN/H_2_O, respectively) with **pDEGA‐BuA‐1⋅XB** more anodic still (154±1 mV vs. 144±2 mV in 97.5 : 2.5 ACN/H_2_O, for **pDEGA‐BuA‐1⋅XB** and **pDEGA‐1⋅XB**, respectively).

Interestingly, the disparity between the *E*_1/2_s of **1⋅XB** and **pDEGA‐1⋅XB** was most pronounced in highly polar solvent systems, with this difference contracting, and then disappearing, on decreasing polarity (Figure 5b). At the point of convergence (*E*_1/2,poly_=*E*_1/2,mon_), the transducers of each host can be assumed to be in near identical local environments and this enables an approximation of microenvironment local dielectric. The dielectric constants of the polymer binding sites can be estimated as being ≈12 for **pDEGA‐1⋅XB** and ≈16 for **pDEGA‐1⋅HB** (see Figures 5b and S8.13, and Supporting Information, Section 8 for more details). The higher local dielectric constant of **pDEGA‐1⋅HB** may be, as indicated earlier, a result of more pronounced penetration of HB accepting solvents (e.g. acetone, ACN, and water) due to the stronger interaction of proto‐triazole units in comparison to iodo‐triazoles in **pDEGA‐1⋅XB**.

### Electrochemical anion sensing by monomeric and polymeric hosts

Electrochemical anion sensing studies were then performed with **1⋅XB** and **pDEGA‐1⋅XB** and their HB analogues (comparisons detailed in Supporting Information, Section S9) in two of the solvent systems used for the ^1^H NMR binding studies: 97.5 : 2.5 acetone/H_2_O and 97.5 : 2.5 ACN/H_2_O. In both solvent systems, significant cathodic shifts of the host's *E*_1/2_ (Δ*E*_1/2_) were observed upon addition of a range of anions (e.g. 201 mV cathodic shift with **pDEGA‐1⋅XB** in response to H_2_PO_4_^−^). A selectivity trend in terms of maximum cathodic shift Δ*E*_max_: H_2_PO_4_^−^>HSO_4_^−^>Cl^−^>Br^−^ was observed for both **1⋅XB** and **pDEGA‐1⋅XB**, with the XB hosts generally exhibiting larger responses to the halides than HB hosts.^[34]^ Interestingly, the Δ*E*_max_ selectivity trend is the reverse of that determined with the neutral hosts by ^1^H NMR. However, it is worth noting here that selectivity in terms of Δ*E* response is not a simple reflection of binding by the neutral hosts, but a combination of several factors such as interactions with the charged (oxidised) hosts, or ion‐specific signal enhancements.^[12a]^ Importantly, significant anion binding induced cathodic shift response enhancements (Δ*E*_1/2_) were observed for polymeric host **pDEGA‐1⋅XB** in comparison to **1⋅XB** in all cases (Table 3; Figure 5c).

**Table 3:** Maximum cathodic shifts Δ*E*_max_ [mV] of the *E*_1/2_ of **1⋅XB** and **pDEGA‐1⋅XB** in response to a range of anions, in 97.5 : 2.5 acetone/H_2_O and 97.5 : 2.5 ACN/H_2_O.

	97.5 : 2.5 acetone/H_2_O			97.5 : 2.5 ACN/H_2_O		
Anion	**1⋅XB**	**pDEGA‐1⋅XB**	Δ*E*_**pDEGA‐1⋅XB**_ /Δ*E*_**1⋅XB**_	**1⋅XB**	**pDEGA‐1⋅XB**	Δ*E*_**pDEGA‐1⋅XB**_ /Δ*E*_**1⋅XB**_
Cl^−^	−54	−75	1.4	−54	−69	1.3
Br^−^	−46	−74	1.6	−52	−54	1.1
HSO_4_^−^	−77	−98	1.3	−54	−76	1.4
H_2_PO_4_^−^	−157	−201	1.3	−115	−147	1.3

A control experiment in which the **1⋅XB** monomer was titrated with Br^−^ in presence of 9 equiv. of a non‐receptive pDEGA homopolymer generated no detectable difference in electrochemical response (−2 mV, <4% difference wrt. the response of **1⋅XB** alone) in comparison to titration of **1⋅XB** without pDEGA, proving that the polymeric integration is an integral and necessary requirement for binding enhancements over the monomer to be observed (Supporting Information, Figures S9.3 & S9.4).

Although the polymer vs. monomer enhancements resolved by NMR are replicated in these electroanalyses (for both HB and XB), the differences between **pDEGA‐BuA‐1⋅XB** and **pDEGA‐1⋅XB** are not (in that these two polymers show very similar resolved responses). This may be a reflection of electrolyte ingress during voltammetric analysis and is the subject of further work. These results, nonetheless, clearly indicate that the encapsulation of Lewis acidic motifs within polymers presents an attractive platform for the development of derived anion sensors.

## Conclusion

In this work we report, for the first time, a detailed comparison of anion binding between monomeric and polymeric sigma‐hole hosts. Significantly, we demonstrate that the incorporation of relatively simple XB binding motifs into co‐polymeric scaffold structures results in a significant enhancement of anion binding strength across a series of polar organic‐aqueous solvent systems. This so‐called specific polymeric enhancement effect was quantified by comprehensive anion binding and sensing studies across a range of organic/aqueous solvent media. This approach offers a highly attractive alternative to the challenging synthesis of highly preorganised and structurally complex discrete molecular hosts. Moreover, the considered use of co‐polymers supports a unique resistance to the diminished anion binding strength typically associated with increased bulk solution polarity. These benefits result from the generation of a low dielectric constant microenvironment around the binding units in which there is significant solvent exclusion and are most marked when co‐monomers of lower polarity are included (enabling, for example, a 47‐fold iodide binding enhancement over the monomer). The polymeric anion binding enhancements were particularly pronounced for the XB polymers, which significantly outperformed their HB analogues, observations we attribute to the higher hydrophobicity of the XB centers.

The incorporation of electrochemically active ferrocene units into the structure of the receptive binding units enabled an estimation of the value of local dielectric constant, with resolved half‐wave potentials (*E*_1/2_) both corroborating anion binding enhancements and supporting the exploitation of these polymers in sensing applications.

The potent polymeric sigma‐hole host architectural design serves as a powerful alternative to more traditional approaches in supramolecular ion recognition and is natively highly chemically tuneable; we anticipate that the toolbox this opens will not only support the generation of highly engineered binding configurations, but also support practical translation.

## Conflict of interest

The authors declare no conflict of interest.

1

## Supporting information

As a service to our authors and readers, this journal provides supporting information supplied by the authors. Such materials are peer reviewed and may be re‐organized for online delivery, but are not copy‐edited or typeset. Technical support issues arising from supporting information (other than missing files) should be addressed to the authors.

Supporting Information

## Data Availability

The data that support the findings of this study are available in the supplementary material of this article.

## References

[1a] N. Busschaert , C. Caltagirone , W. Van Rossom , P. A. Gale , Chem. Rev. 2015, 115, 8038–8155; 25996028 10.1021/acs.chemrev.5b00099

[1b] M. A. Shannon , P. W. Bohn , M. Elimelech , J. G. Georgiadis , B. J. Mariñas , A. M. Mayes , Nature 2008, 452, 301–310; 18354474 10.1038/nature06599

[1c] X. Ji , R. Wu , L. Long , C. Guo , N. M. Khashab , F. Huang , J. L. Sessler , J. Am. Chem. Soc. 2018, 140, 2777–2780; 29437394 10.1021/jacs.7b13656

[1d] Q. Zhang , Y. Zhou , M. Ahmed , N. M. Khashab , W. Han , H. Wang , Z. A. Page , J. L. Sessler , J. Mater. Chem. A 2022, 10, 15297–15308;

[1e] L. K. Macreadie , A. M. Gilchrist , D. A. McNaughton , W. G. Ryder , M. Fares , P. A. Gale , Chem 2022, 8, 46–118;

[1f] O. García Mancheño , Anion-Binding Catalysis, Wiley-VCH, Weinheim, 2022.

[2a] Y. Liu , A. Sengupta , K. Raghavachari , A. H. Flood , Chem 2017, 3, 411–427;

[2b] S. Kubik , Chem. Soc. Rev. 2010, 39, 3648–3663; 20617241 10.1039/b926166b

[2c] F. Sommer , S. Kubik , Org. Biomol. Chem. 2014, 12, 8851–8860; 25254969 10.1039/c4ob01497a

[2d] M. J. Langton , C. J. Serpell , P. D. Beer , Angew. Chem. Int. Ed. 2016, 55, 1974–1987; 10.1002/anie.201506589 PMC4755225 26612067

[2e] S. L. Cockroft , Chem 2017, 3, 383–384.

[3] P. Molina , F. Zapata , A. Caballero , Chem. Rev. 2017, 117, 9907–9972. 28665114 10.1021/acs.chemrev.6b00814

[4a] Y. Liu , F. C. Parks , E. G. Sheetz , C. Chen , A. H. Flood , J. Am. Chem. Soc. 2021, 143, 3191–3204; 33596052 10.1021/jacs.0c12562

[4b] A. Sengupta , Y. Liu , A. H. Flood , K. Raghavachari , Chem. Eur. J. 2018, 24, 14409–14417; 30036449 10.1002/chem.201802657

[4c] T. J. Sherbow , H. A. Fargher , M. M. Haley , M. D. Pluth , D. W. Johnson , J. Org. Chem. 2020, 85, 12367–12373; 32916056 10.1021/acs.joc.0c01616 PMC10778081

[4d] F. C. Parks , E. G. Sheetz , S. R. Stutsman , A. Lutolli , S. Debnath , K. Raghavachari , A. H. Flood , J. Am. Chem. Soc. 2022, 144, 1274–1287. 35015538 10.1021/jacs.1c10758

[5] P. Chakrabarti , J. Mol. Biol. 1993, 234, 463–482. 8230226 10.1006/jmbi.1993.1599

[6] A. Fernández , A. Crespo , Chem. Soc. Rev. 2008, 37, 2373–2382. 18949110 10.1039/b804150b

[7] S. Kubik , Acc. Chem. Res. 2017, 50, 2870–2878. 29125287 10.1021/acs.accounts.7b00458

[8a] K. M. Bąk , K. Porfyrakis , J. J. Davis , P. D. Beer , Mater. Chem. Front. 2020, 4, 1052–1073;

[8b] M. J. Langton , S. W. Robinson , I. Marques , V. Félix , P. D. Beer , Nat. Chem. 2014, 6, 1039–1043. 25411880 10.1038/nchem.2111

[9a] Y. Liu , W. Zhao , C. Chen , A. H. Flood , Science 2019, 365, 159–161; 31123106 10.1126/science.aaw5145

[9b] R. Custelcean , Chem. Soc. Rev. 2014, 43, 1813–1824. 24384869 10.1039/c3cs60371g

[10a] P. Sokkalingam , J. Shraberg , S. W. Rick , B. C. Gibb , J. Am. Chem. Soc. 2016, 138, 48–51; 26702712 10.1021/jacs.5b10937 PMC5571644

[10b] R. S. Carnegie , C. L. D. Gibb , B. C. Gibb , Angew. Chem. Int. Ed. 2014, 53, 11498–11500; 10.1002/anie.201405796 PMC4214138 25196481

[10c] M. Lisbjerg , B. E. Nielsen , B. O. Milhøj , S. P. A. Sauer , M. Pittelkow , Org. Biomol. Chem. 2015, 13, 369–373; 25407665 10.1039/c4ob02211d

[10d] N. N. Andersen , K. Eriksen , M. Lisbjerg , M. E. Ottesen , B. O. Milhøj , S. P. A. Sauer , M. Pittelkow , J. Org. Chem. 2019, 84, 2577–2584; 30721069 10.1021/acs.joc.8b02797

[10e] M. A. Yawer , V. Havel , V. Sindelar , Angew. Chem. Int. Ed. 2015, 54, 276–279; 10.1002/anie.201409895 25385515

[10f] T. Lizal , V. Sindelar , Isr. J. Chem. 2018, 58, 326–333.

[11a] J. Suk , K. Jeong , J. Am. Chem. Soc. 2008, 130, 11868–11869; 18700772 10.1021/ja804845m

[11b] A. Borissov , I. Marques , J. Y. C. Lim , V. Félix , M. D. Smith , P. D. Beer , J. Am. Chem. Soc. 2019, 141, 4119–4129; 30730716 10.1021/jacs.9b00148

[11c] H. Juwarker , K. Jeong , Chem. Soc. Rev. 2010, 39, 3664–3674; 20730154 10.1039/b926162c

[11d] E. A. John , C. J. Massena , O. B. Berryman , Chem. Rev. 2020, 120, 2759–2782; 32039583 10.1021/acs.chemrev.9b00583 PMC7179952

[11e] K. M. Bąk , K. Masłowska , M. J. Chmielewski , Org. Biomol. Chem. 2017, 15, 5968–5975. 28675234 10.1039/c7ob01358b

[12a] R. Hein , X. Li , P. D. Beer , J. J. Davis , Chem. Sci. 2021, 12, 2433–2440; 10.1039/d0sc06210c PMC8179314 34164009

[12b] S. C. Patrick , R. Hein , A. Docker , P. D. Beer , J. J. Davis , Chem. Eur. J. 2021, 27, 10201–10209; 33881781 10.1002/chem.202101102 PMC8360193

[12c] R. Hein , A. Borissov , M. D. Smith , P. D. Beer , J. J. Davis , Chem. Commun. 2019, 55, 4849–4852; 10.1039/c9cc00335e 30950463

[12d] J. F. Neal , W. Zhao , A. J. Grooms , M. A. Smeltzer , B. M. Shook , A. H. Flood , H. C. Allen , J. Am. Chem. Soc. 2019, 141, 7876–7886. 31025857 10.1021/jacs.9b02148

[13a] P. A. Gale , C. Caltagirone , Chem. Soc. Rev. 2015, 44, 4212–4227; 24975326 10.1039/c4cs00179f

[13b] H. M. Tay , P. D. Beer , Org. Biomol. Chem. 2021, 19, 4652–4677; 33982045 10.1039/d1ob00601k

[13c] M. J. Langton , P. D. Beer , Acc. Chem. Res. 2014, 47, 1935–1949. 24708030 10.1021/ar500012a

[14a] B. Adhikari , S. Majumdar , Prog. Polym. Sci. 2004, 29, 699–766;

[14b] U. Lange , N. V. Roznyatovskaya , V. M. Mirsky , Anal. Chim. Acta 2008, 614, 1–26. 18405677 10.1016/j.aca.2008.02.068

[15] A. Aydogan , D. J. Coady , S. K. Kim , A. Akar , C. W. Bielawski , M. Marquez , J. L. Sessler , Angew. Chem. Int. Ed. 2008, 47, 9648–9652; 10.1002/anie.200803970 PMC2862972 18925601

[16] K. P. McDonald , B. Qiao , E. B. Twum , S. Lee , P. J. Gamache , C. Chen , Y. Yi , A. H. Flood , Chem. Commun. 2014, 50, 13285–13288. 10.1039/c4cc03362k 25233076

[17a] L. Zhu , C. Yang , W. Zhang , J. Qin , Polymer 2008, 49, 217–224;

[17b] R. Kakuchi , S. Nagata , R. Sakai , I. Otsuka , H. Nakade , T. Satoh , T. Kakuchi , Chem. Eur. J. 2008, 14, 10259–10266. 18846603 10.1002/chem.200801235

[18] A. Rostami , C. J. Wei , G. Guérin , M. S. Taylor , Angew. Chem. Int. Ed. 2011, 50, 2059–2062; 10.1002/anie.201006884 21344551

[19] J. Romański , P. Piątek , Chem. Commun. 2012, 48, 11346–11348. 10.1039/c2cc36607j 23080000

[20a] B. A. Rogers , H. I. Okur , C. Yan , T. Yang , J. Heyda , P. S. Cremer , Nat. Chem. 2022, 14, 40–45; 34725491 10.1038/s41557-021-00805-z

[20b] K. B. Rembert , H. I. Okur , C. Hilty , P. S. Cremer , Langmuir 2015, 31, 3459–3464. 25764296 10.1021/acs.langmuir.5b00127

[21] J. Y. C. Lim , P. D. Beer , Chem 2018, 4, 731–783.

[22] J. Pancholi , P. D. Beer , Coord. Chem. Rev. 2020, 416, 213281.

[23a] J. Y. C. Lim , M. J. Cunningham , J. J. Davis , P. D. Beer , Chem. Commun. 2015, 51, 14640–14643; 10.1039/c5cc05704c 26289779

[23b] A. Docker , C. H. Guthrie , H. Kuhn , P. D. Beer , Angew. Chem. Int. Ed. 2021, 60, 21973–21978; 10.1002/anie.202108591 PMC8518858 34297867

[23c] R. Hein , A. Docker , J. J. Davis , P. D. Beer , J. Am. Chem. Soc. 2022, 144, 8827–8836. 35522996 10.1021/jacs.2c02924 PMC9121379

[24a] R. Kampes , S. Zechel , M. D. Hager , U. S. Schubert , Chem. Sci. 2021, 12, 9275–9286; 34349897 10.1039/d1sc02608a PMC8278954

[24b] R. Tepper , S. Bode , R. Geitner , M. Jäger , H. Görls , J. Vitz , B. Dietzek , M. Schmitt , J. Popp , M. D. Hager , U. S. Schubert , Angew. Chem. Int. Ed. 2017, 56, 4047–4051; 10.1002/anie.201610406 28266170

[25] L. E. Bickerton , A. Docker , A. J. Sterling , H. Kuhn , F. Duarte , P. D. Beer , M. J. Langton , Chem. Eur. J. 2021, 27, 11738–11745. 34014001 10.1002/chem.202101681 PMC8453555

[26a] R. Hein , P. D. Beer , Chem. Sci. 2022, 13, 7098–7125; 35799814 10.1039/d2sc01800d PMC9214886

[26b] R. Hein , P. D. Beer , J. J. Davis , Chem. Rev. 2020, 120, 1888–1935. 31916758 10.1021/acs.chemrev.9b00624

[27a] S. Perrier , Macromolecules 2017, 50, 7433–7447;

[27b] G. Moad , E. Rizzardo , RAFT Polymerization, Wiley-VCH, Weinheim, 2021, pp. 1–13.

[28] G. Vancoillie , D. Frank , R. Hoogenboom , Prog. Polym. Sci. 2014, 39, 1074–1095.

[29] Copolymer **pBuA-1⋅XB** was synthesised, but poor solubility in most solvents of interest prevented anion binding studies, necessitating dilution with DEGA units.

[30] D. Brynn Hibbert , P. Thordarson , Chem. Commun. 2016, 52, 12792–12805. 10.1039/c6cc03888c 27779264

[31] The impact of signal broadening, as well as the potential impact of error in host/guest concentration and data sampling on resolved binding constants is detailed and quantified in depth in Supporting Information, Section S6.2. These analyses confirm that resolved polymer binding constants are within 20 % error.

[32] Monitoring ^1^H NMR signals of the polymers is associated with a larger error than that of monomers due to changes of signal shape during titration experiments. This reflects the different possible positions of the receptive units along each polymer strand, as well as each signal representing the average of all conformations of polymer in solution. See a detailed discussion of the determination of binding constants and associated error in Supporting Information, Section S6.

[33] S. C. Patrick , R. Hein , P. D. Beer , J. J. Davis , J. Am. Chem. Soc. 2021, 143, 19199–19206. 34730337 10.1021/jacs.1c09743

[34] Overlap in oxidation potential with Fc|Fc^+^ prevented electrochemical binding studies with iodide.

