# Supplementary material for: Engineered Binding Microenvironments in Halogen Bonding Polymers for Enhanced Anion Sensing
Source: Angew Chem Int Ed Engl. 2023 Feb 23;62(14):e202300867. doi: 10.1002/anie.202300867 (PMC10946961; doi:10.1002/anie.202300867)
Supplement: Supplementary file 1 — Supporting Information [file ANIE-62-0-s001.pdf]

Angewandte Chemie
Eine Zeitschrift der Gesellschaft Deutscher Chemiker

Supporting Information

Engineered Binding Microenvironments in Halogen Bonding Polymers for Enhanced Anion Sensing

K. M. Bąk, S. C. Patrick, X. Li, P. D. Beer, J. J. Davis**

Table of Contents:

S1 Materials and Experimental Details **3**

S1.1 General Information 3

S1.2 NMR Measurements 3

S1.3 GPC Measurements 3

S1.4 Electrochemical Measurements 4

S1.5 Data Analysis and Fitting of Binding Isotherms 4

S2 Synthesis **5**

S2.1 Synthesis of **1·XB/HB** 5

S2.2 Synthesis of Polymeric Hosts 13

S3 Polymer Characterisation **22**

S3.1 ATR-FTIR Characterisation 22

S3.2 GPC Data 23

S4 Anion Binding Performance of 1·XB/HB by ^{1}H-NMR **25**

S5 Anion Binding Performance of pDEGA-1·XB/HB by ^{1}H-NMR **38**

S6 Discussion of Errors Associated with K by 1H NMR Titrations **51**

S6.1 Concentration of the Binding Units 51

S6.2 Stability of the Determined Binding Constants 52

S6.3 Repetition of Titration Experiments 56

S7 Role of a Cation in Anion Binding by Polymeric Receptors **58**

S8 Electrochemical Characterisation **61**

S8.1 Electrochemical Characterisation of Hosts in 97.5:2.5 acetone/H_2O 61

S8.2 Electrochemical Characterisation of Hosts in 97.5:2.5 ACN/H_2O 63

S9 Electrochemical Sensing Data **67**

S9.1 Electrochemical Anion Sensing Performance of **1·HB** and **pDEGA-1·HB** 67

S9.2 NMR vs Echem Anion Binding Constants 69

S9.3 **pDEGA** Homopolymer Electrochemical Control 70

S9.4 Tabulated Nernst Binding Isotherm Data 71

S10 References **73**

S1 Materials and Experimental Details

S1.1 General Information

All experiments were performed in the presence of air, at room temperature, unless otherwise stated. All commercially available chemicals and solvents were used as received without further purification, other than di(ethylene glycol) ethyl ether acrylate (DEGA) and n-butyl acrylate (BuA) monomers which were passed through a basic alumina column immediately prior to use to remove inhibitors. All hygroscopic tetrabutylammonium (TBA) salts were stored in vacuum desiccators at room temperature. Ultrapure water was obtained from a Milli-Q system (18.2 MΩcm). Supporting electrolyte ($TBAClO_4$ from Sigma Aldrich) was of electrochemical grade. FT-ATR-IR spectra were measured on an IRTracer-100 (Shimadzu). Mass spectra were recorded on a Bruker µTOF spectrometer. Where mixtures of solvents were used, ratios are reported by volume. GPC was carried out with a Shimadzu LC-20AD GPC instrument equipped with two PSS SDV 5 µm linear M columns and a refractive index (RI) detector, calibrated with an Agilent EasiVial PS 2 mL pre-weighed calibration kit.

S1.2 NMR Measurements

Routine NMR spectra were recorded on Bruker NMR spectrometers AVIII HD 400, with ^{1}H-NMR anion binding titrations recorded on a Bruker AVIII HD 500 spectrometer. Chemical shifts are given in parts per million (ppm) relative to the residual solvent peak. Unless otherwise stated, a 50 mM solution of the chosen TBA salt of an anion was added to 0.5 mL containing 1 mM of the binding unit (**1·XB/HB**) in the same solvent media as the TBA salt. Each titration isotherm comprises 17 data points, corresponding to 0.0, 0.2, 0.4, 0.6, 0.8, 1.0, 1.2, 1.4, 1.6, 1.8, 2, 2.5, 3.0, 4.0, 5.0, 7.0, 10.0 equivalents of added guest anion. All anion binding events were found to be fast on the NMR timescale. Chemical shifts of polymer proton signals were determined using the MestReNova (v. 14.2.0) peak picking algorithm, ensuring that the value corresponds to the centre of the signal. Binding constants were determined using BindFit,[1] applying a 1:1 host-guest binding model in all cases. Fitting using 2:1 host-guest model failed in all cases.

S1.3 GPC Measurements

Samples were prepared by dissolving approximately 5 mg of each polymer in 1 mL HPLC grade THF (inhibitor free), then filtered through 2 µm PTFE syringe filters. Each sample was then injected into the GPC instrument where an eluent of HPLC grade THF (inhibitor free) was used at a flow rate of 1.0 mL min^{-1} (heated to 30 °C). RI and UV detectors were calibrated using narrow molecular weight polystyrene standards covering a M_w range of 900-360,000 g mol^{-1}. Analysis of GPC data was completed with Shimadzu GPC post-run program.

S1.4 Electrochemical Measurements

All experiments were conducted using an Autolab Potentiostat (Metrohm) with a three-electrode setup equipped with a glassy carbon working electrode for all solution-phase experiments (BaSi, 3 mm diameter, a platinum wire counter electrode and a non-aqueous Ag|$AgNO_3$ reference electrode (with an inner filling solution of 10 mM $AgNO_3$, 100 mM $TBAClO_4$ in ACN). Glassy carbon disc electrodes were polished mechanically with a 0.05 µm alumina slurry for 2 min, followed by sonication in 1:1 H_2O/EtOH for 2 min, then rinsed with the solvent used for characterisation/sensing studies prior to all electrochemical measurements, and in between each addition during anion sensing studies. All potentials are reported wrt. an external ferrocene standard, unless otherwise stated. All experiments were carried out with 100 mM $TBAClO_4$ as a supporting electrolyte, and a constant ionic strength of 100 mM TBA-anion was maintained throughout. Cyclic voltammetry measurements were carried at with a step potential of 2 mV at a scan rate of 0.1 V s^{-1}, and measurements at varying scan rates were performed at: 0.025, 0.05, 0.075, 0.1, 0.2, 0.4, 0.6 and 0.8 V s^{-1}. The half wave potentials ($E_{1/2}$) of all hosts were determined from square wave voltammetry (SWV) measurements, which were conducted with a step potential of 2 mV, amplitude of 20 mV and frequency of 25 Hz.

Titrations were performed with 100 µM host in the chosen solvent system, with 100 mM $TBAClO_4$ as a supporting electrolyte. The ionic strength (and host concentration in diffusive experiments) was kept constant at 100 mM throughout by sequential additions of 100 mM TBAX (X = Cl^-, Br^-, HSO_4^- or $H_2PO_4^-$) up to a final anion concentration of 50 mM anion in all cases. Voltammetric responses were assessed by the change in the receptors' $E_{1/2}$, as monitored by SWV.

S1.5 Data Analysis and Fitting of Binding Isotherms

All analysis and fitting of electrochemical data was carried out with OriginPro 2017. Analysis of the sensor responses was carried out via eqns. 1-2. Fitting of the sensing isotherms according to eqn. 2 was carried out without any restriction of the parameters unless stated otherwise. In some cases, nonsensical values were obtained from fits with eqn. 2 (e.g. negative values for K_{red}) which is chemically not possible, and is purely the result of the fitting (affording the best mathematical fit to eqn. 2), which can be restricted to K_{Red} = 0 to afford similar fits.

$$\Delta E = -\frac{RT}{nF}\ln\left(\frac{K_{Ox}}{K_{Red}}\right) \qquad \text{(Eqn 1)}$$

$$\Delta E = -\frac{RT}{nF}\ln\left(\frac{1 + K_{Ox}[A^-]}{1 + K_{Red}[A^-]}\right) \qquad \text{(Eqn 2)}$$

S2 Synthesis & Characterisation

S2.1 Synthesis of 1·XB/HB

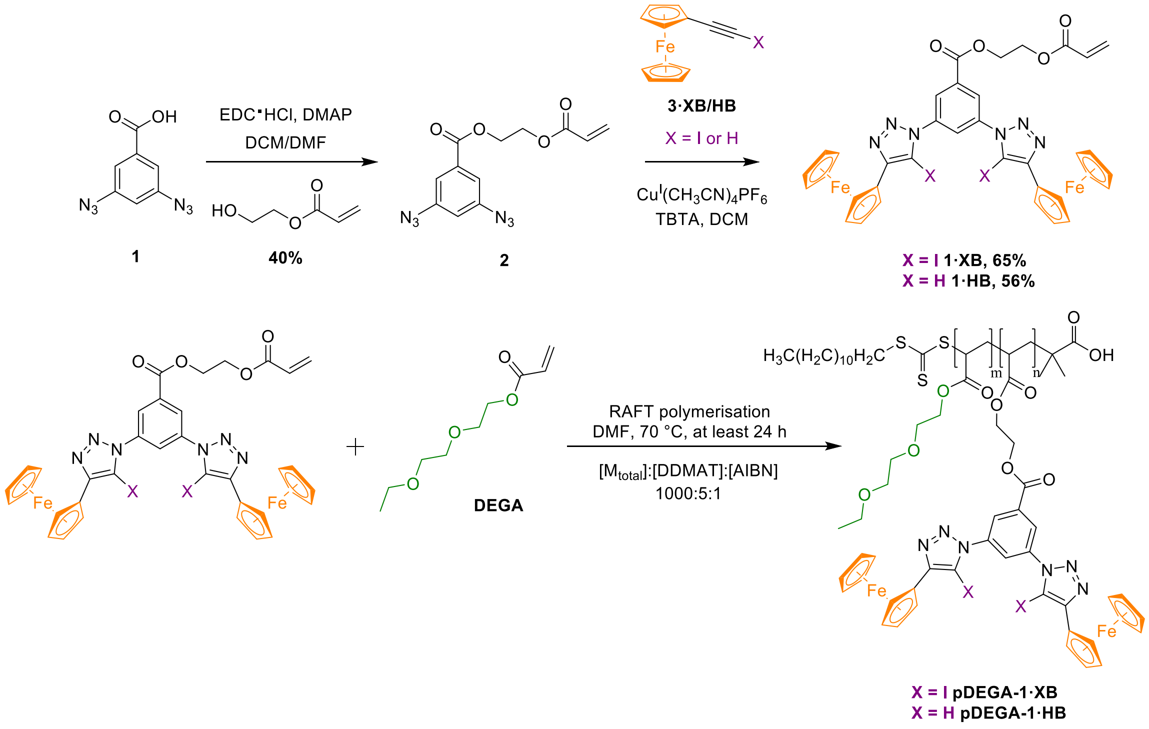

Figure S2.1. Reaction scheme to **1·XB/HB** and **pDEGA-1·XB/HB**.

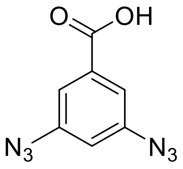

1

3,5-diazidobenzoic acid, **1** was prepared from 3,5-diaminobenzoic acid following a previously reported procedure.[2] ^{1}H NMR in accordance with the published data.

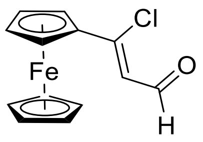

4

(2-Formyl-1-chlorovinyl)-ferrocene 4 was prepared following a previously published procedure.[3] A stirred solution of acetylferrocene (2.00 g, 8.77 mmol) in dry DMF (25 mL) was purged with N_2 for 15 min and then cooled to 0 °C in an ice bath. The solution was stirred for 15 min under an N_2 atmosphere. In a separate flask, dry DMF (5.0 mL) was cooled in an ice bath, to which phosphorus oxychloride (2.6 mL) was added under N_2. The prepared mixture was transferred to the vigorously stirred solution of the acetylferrocene. The addition was completed within 10 min and the stirring was continued at 0°C under N_2 for 1.5 h. 20%

aqueous sodium acetate (65 mL) was cautiously added to the reaction mixture in one portion and stirring was continued for 1 h at RT. The organic phase was separated and the aqueous phase was extracted with CH_2Cl_2. The combined organic phase was dried over anhydrous $MgSO_4$ and solvent was removed *in vacuo*. Purification by column chromatography (SiO_2; hexane/ethyl ether 3:1) afforded product **4** as dark purple powder (1.48 g, 65% yield). ^{1}H NMR and MS in accordance with previously published data.

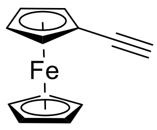

3·HB

Ethynylferrocene 3·HB was prepared following a previously published procedure.[3] A stirred solution of 2-formyl-1-chlorovinyl-ferrocene **4** (1.56 g, 5.69 mmol) in 1,4-dioxane (15 mL) was refluxed for 5 min under N_2. A 0.5 M sodium hydroxide solution (35 mL, 17.5 mmol) was added in one portion to the reaction mixture. The mixture was refluxed for 30 min. The solution was allowed to cool down to RT, poured onto ice, and then it was neutralised to pH = 7 by 2 M hydrochloric acid. The crude mixture was extracted with petroleum ether (2 x 50 mL). The organic phase was separated and washed with saturated $NaHCO_3$ solution (2 × 20 mL), and water (2 × 20 mL). The organic layer was dried over Na_2SO_4 and solvent was removed *in vacuo*. Purification by column chromatography (SiO_2, hexane/DCM 9:1) afforded product **3·HB** as an orange solid (820 mg, 69% yield). ^{1}H NMR and MS in accordance with previously published data.

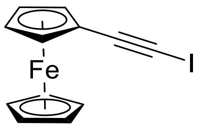

3·XB

Iodoethynylferrocene 3·XB. An aqueous solution of KOH (5.2 M, 1.7 mL) was added at 0°C to a methanol solution (10 mL) of ethynylferrocene **3·HB** (500 mg, 2.38 mmol). The mixture was stirred for 10 min, after which iodine (664 mg, 2.62 mmol) was added in one portion. The solution was warmed to room temperature, protected from light and stirred for 2 h. The solution was poured into water (20 mL) and extracted with Et_2O (20 mL x 3). The organic layers were combined and washed with 10% aq. $Na_2S_2O_3$ solution (20 mL), brine (20 mL) and then dried with $MgSO_4$. The solvent was removed *in vacuo* to afford the product **3·XB** as an orange solid (776 mg, 97% yield). ^{1}H NMR and MS in accordance with previously published data.

^{1}H-NMR (400 MHz, $CDCl_3$), δ: 4.44 (t, J = 1.825, 2H), 4.22 (s, 5H), 4.18 (t, J = 1.825, 2H).
^{13}C-NMR (151 MHz, $CDCl_3$), δ: 92.19, 72.03, 70.35, 69.00, 66.00.
HRMS (ESI+ve) m/z: 335.9085 ($[M+]^+$, $C_{12}FeH_9I$ requires 335.9171)

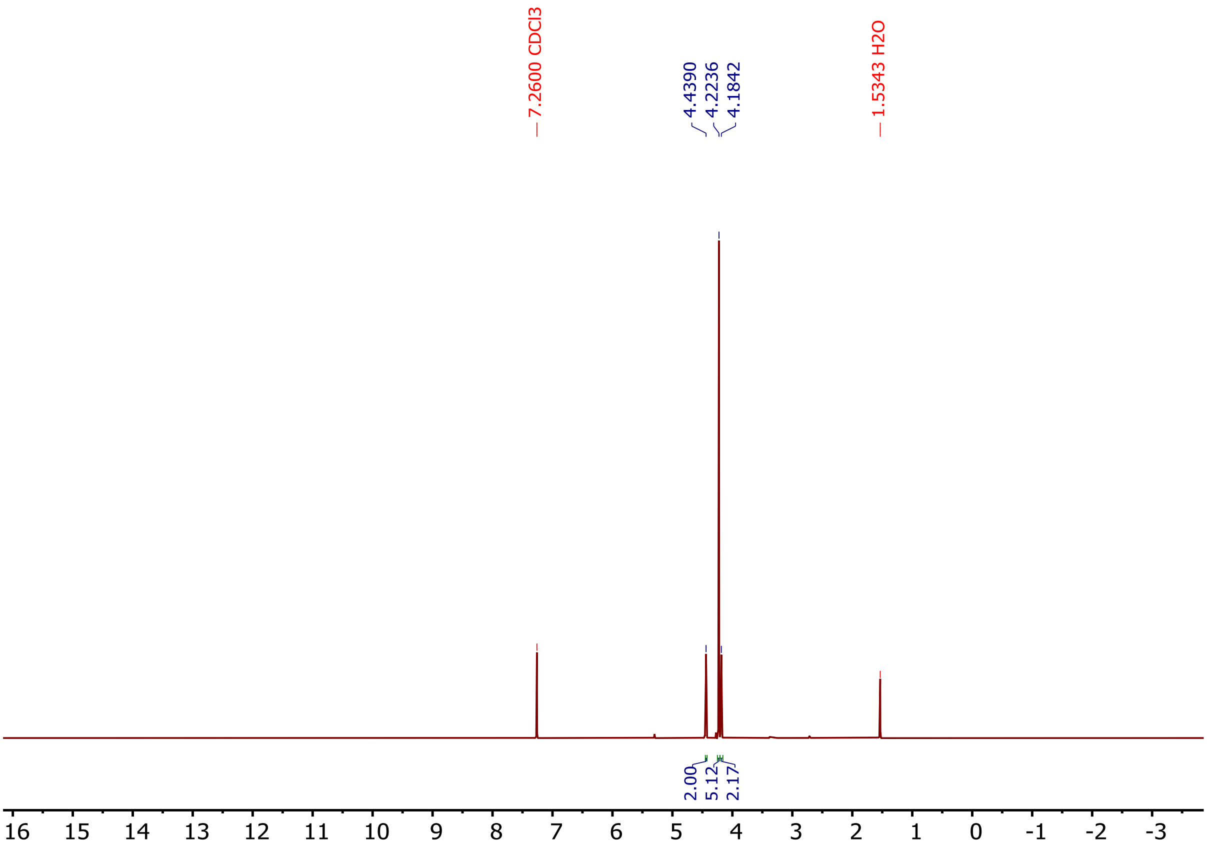

Figure S2.2. ^{1}H NMR spectrum of **3·XB** ($CDCl_3$, 298K, 400 MHz).

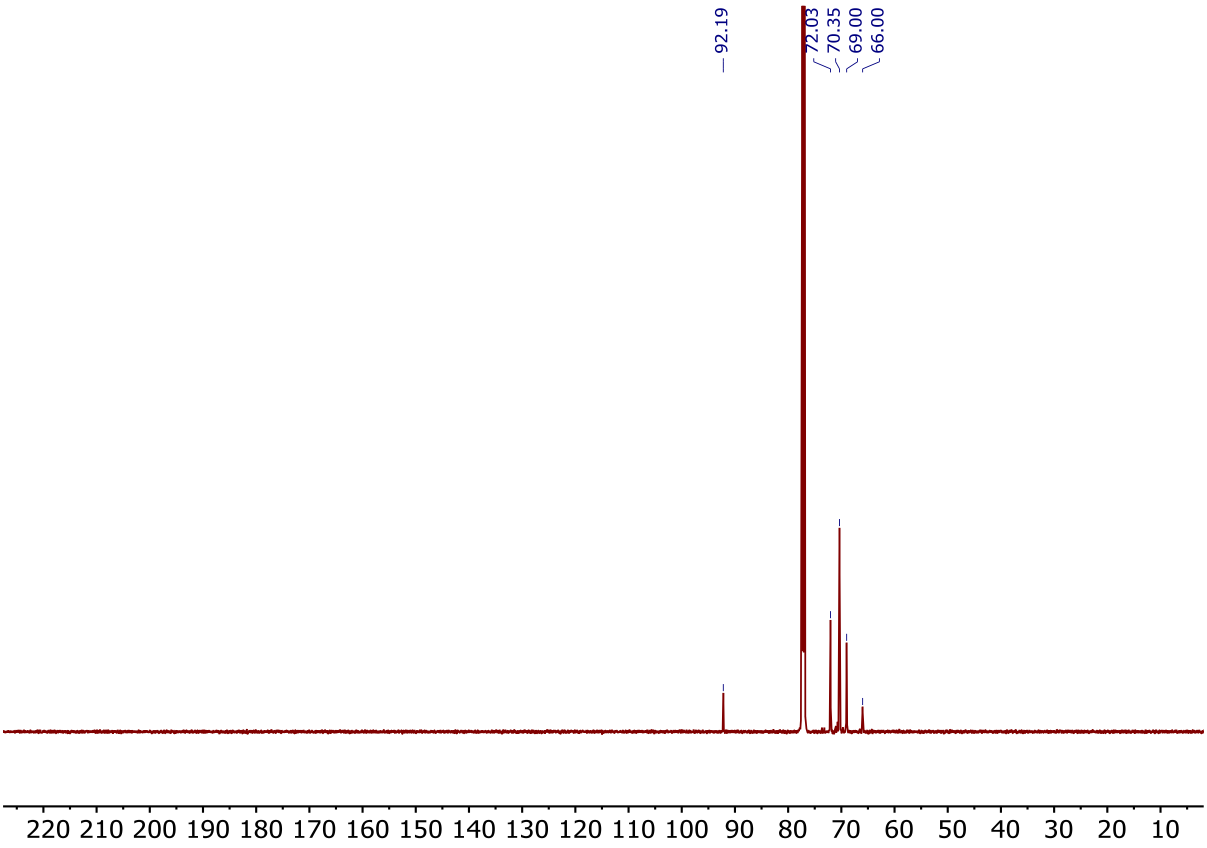

Figure S2.3. ^{13}C NMR spectrum of **3·XB** ($CDCl_3$, 298K, 151 MHz).

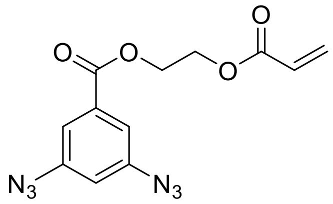

2

2-(Acryloyloxy)ethyl 3,5-diazidobenzoate 2: 3,5-Diazidobenzoic acid, **1** (500 mg, 2.45 mmol) was dissolved in dry CH_2Cl_2 (50 mL) and dry DMF (3 mL). 2-Hydroxyethyl acrylate (1.14 g, 9.80 mmol) was added to the solution followed by EDC·HCl (563 mg, 2.94 mmol) and DMAP (359 mg, 2.94 mmol). The solution was stirred at RT for 48 h under N_2. The reaction mixture was then washed with 5% aq. citric acid solution (3 x 20 mL), saturated aq. $NaHCO_3$ solution (20 mL) and brine (20 mL). The organic layer was dried over $MgSO_4$ and solvent was removed *in vacuo*. Purification by column chromatography (SiO_2; DCM/MeOH 99:1) afforded product **2** as white solid (293 mg, 40% yield).

^{1}H NMR (600 MHz, $CDCl_3$), δ: 7.48 (d, J = 2.05 Hz, 2H), 6.81 (t, J = 2.05 Hz, 1H), 6.45 (dd, 1H), 6.16 (dd, 1H) 5.88 (dd, 1H), 4.58 (m, 2H), 4.51 (m, 2H).
^{13}C NMR (151 MHz, $CDCl_3$), δ: 166.01, 164.78, 142.50, 133.06, 131.77, 128.03, 116.55, 114.18, 63.50, 62.11.
HRMS (ESI+ve) m/z: 325.0643 ($[M+Na]^+$, $C_{12}H_{10}N_6O_4Na$ requires 325.0656)

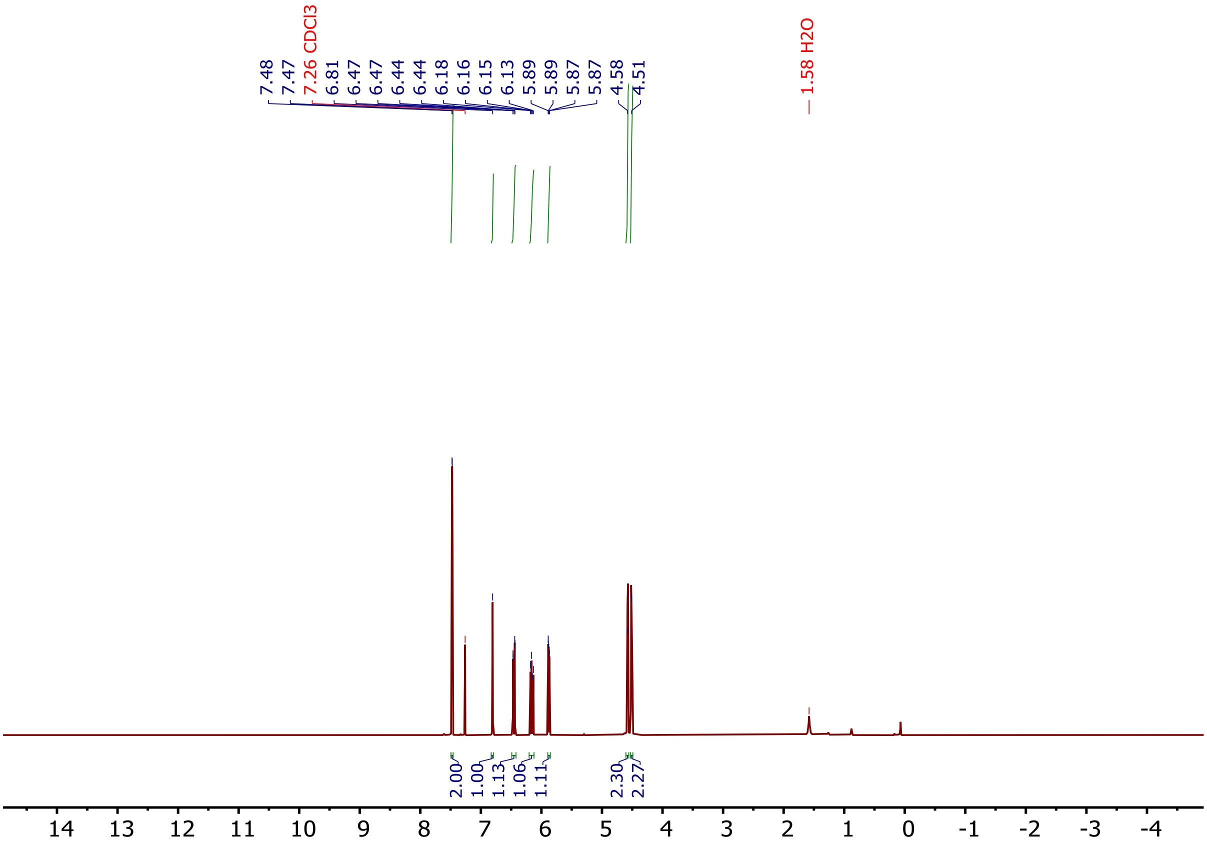

Figure S2.4. ^{1}H NMR spectrum of **2** ($CDCl_3$, 298K, 600 MHz).

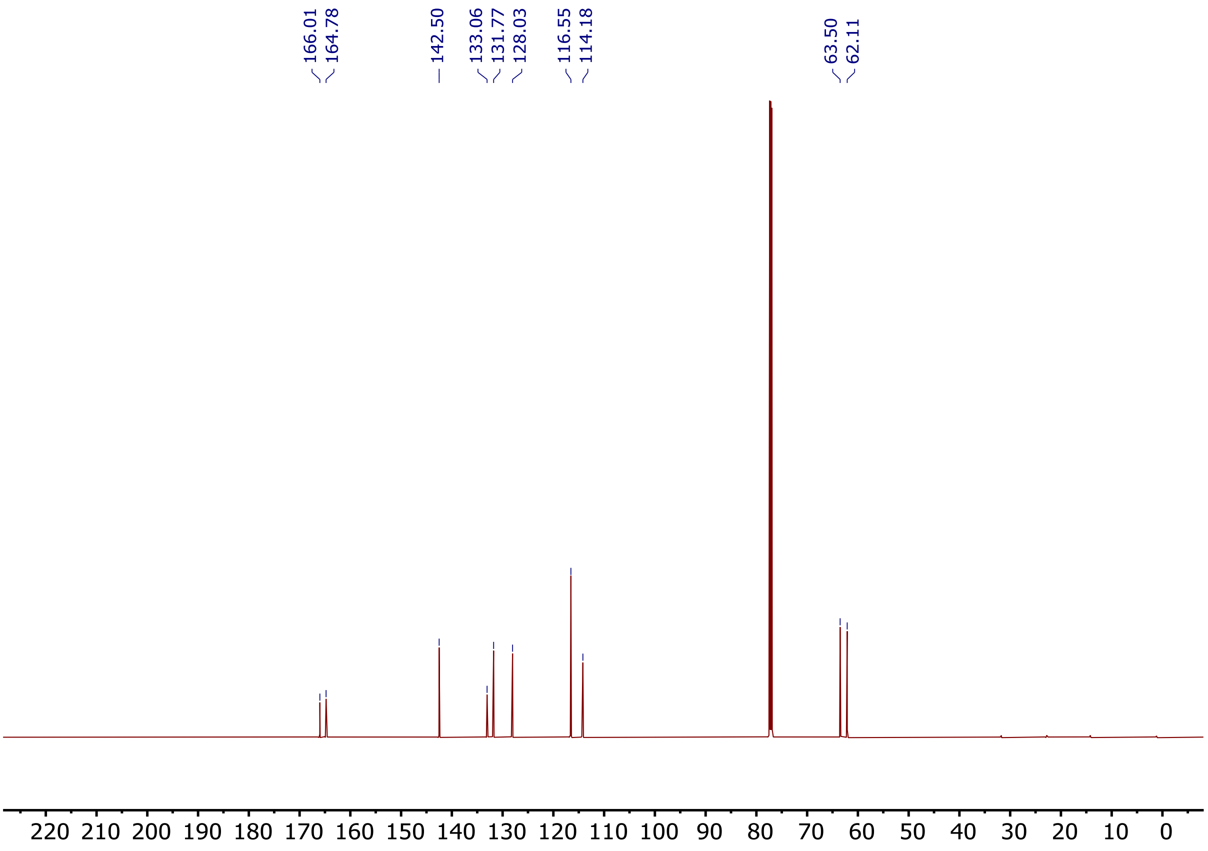

Figure S2.5. ^{13}C NMR spectrum of **2** in ($CDCl_3$, 298K, 151 MHz).

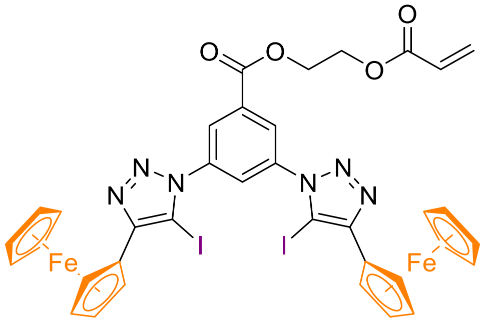

1·XB

$[Cu(CH_3CN)_4]PF_6$ (123 mg, 0.33 mmol) and tris((1-benzyl-4-triazolyl)methyl)amine (TBTA, 176 mg, 0.331 mmol) were dissolved in DCM (anhydrous, 2 mL). The mixture was stirred for 30 min under N_2. Then iodoethynylferrocene, **3·XB** (445 mg, 1.33 mmol) and **2** (200 mg, 0.66 mmol) were added. The reaction mixture was protected from light and stirred overnight under N_2. Next, it was washed with aqueous EDTA (10%)/NH_3 (2%) solution (3 x 20 mL), followed by brine (20 mL). The organic fractions were combined and dried over $MgSO_4$. The solvent was removed in vacuo to give the crude product. Purification by flash chromatography (SiO_2, DCM/MeOH 99:1) afforded product **1·XB** as a crystalline orange solid (420 mg, 65% yield).

^{1}H NMR (400 MHz, $CDCl_3$), δ: 8.50 (d, J = 2.03 Hz, 2H), 8.14 (t, J = 2.03 Hz, 1H), 6.46 (dd, 1H), 6.17 (dd, 1H) 5.88 (dd, 1H), 5.10 (t, J = 1.85 Hz, 4H) 4.68 (m, 2H), 4.56 (m, 2H), 4.41 (t, J = 1.85 Hz, 4H), 4.20 (s, 10H).

^{13}C NMR (151 MHz, $CDCl_3$), δ: 165.98, 163.81, 151.52, 137.91, 132.56, 131.98, 128.41, 128.19, 127.99, 75.08, 74.51, 70.10, 69.62, 67.86, 64.07, 62.10.

HRMS (ESI+ve) m/z: 974.9033 ($[M+H]^+$, $C_{36}H_{29}Fe_2I_2N_6O_4$ requires 974.9033)

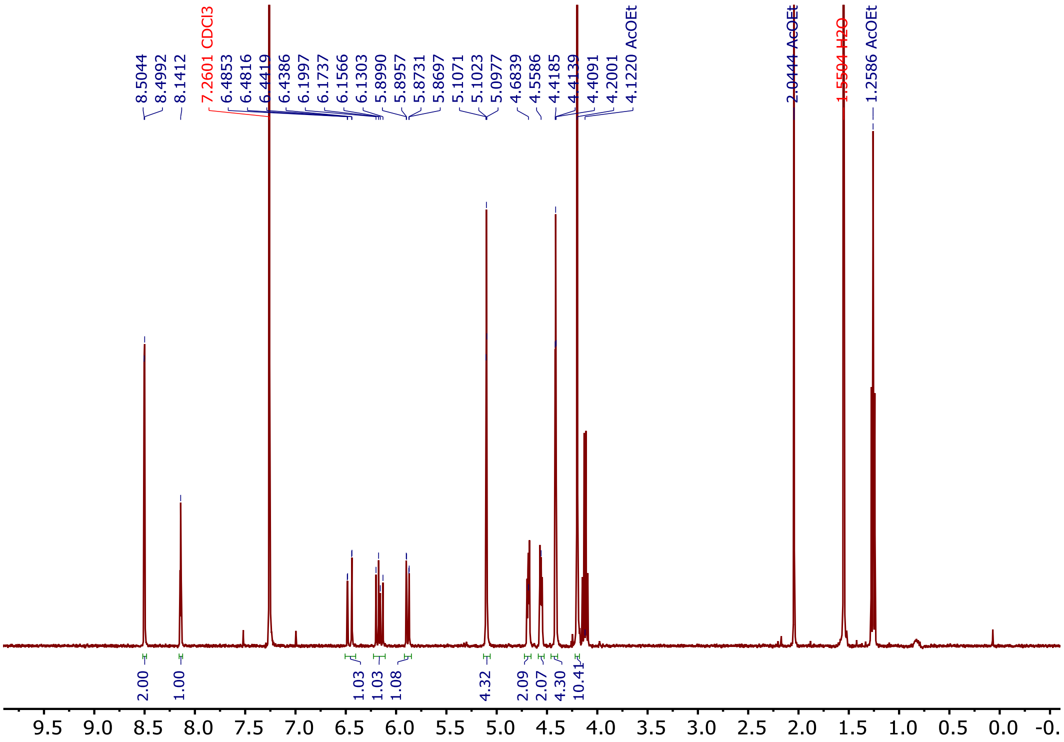

Figure S2.6. ^{1}H NMR spectrum of **1·XB** ($CDCl_3$, 298K, 400 MHz).

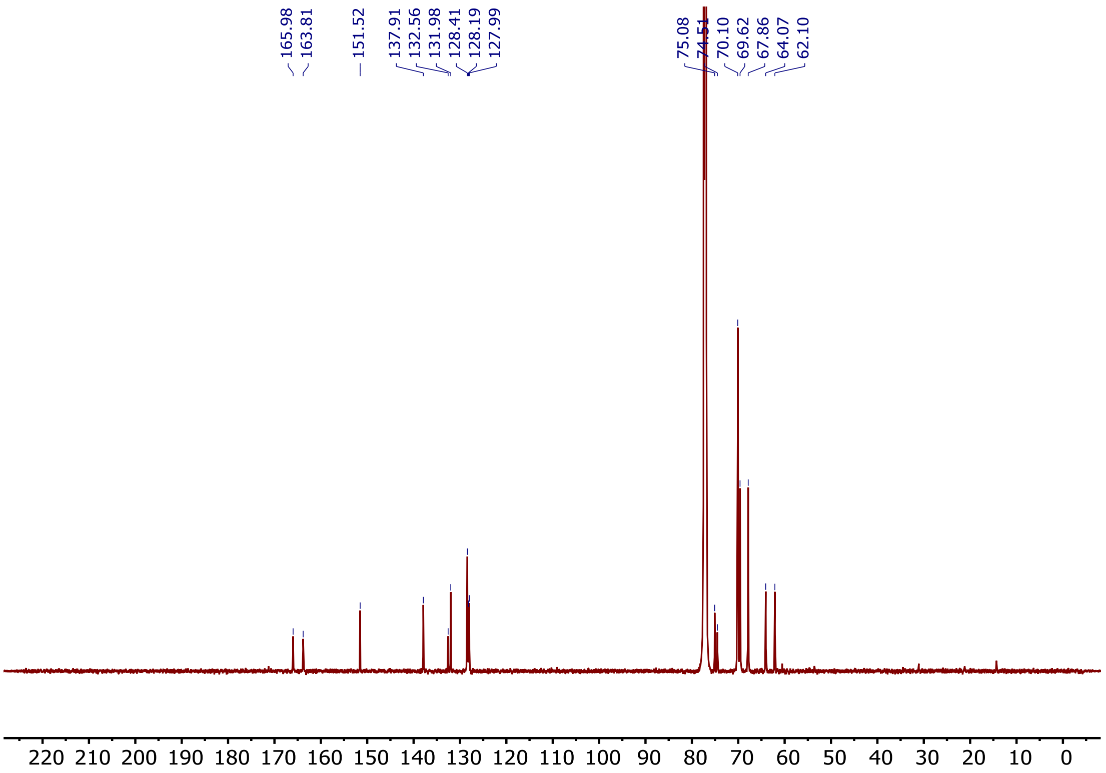

Figure S2.7. ^{13}C NMR spectrum of **1·XB** in ($CDCl_3$, 298K, 151 MHz).

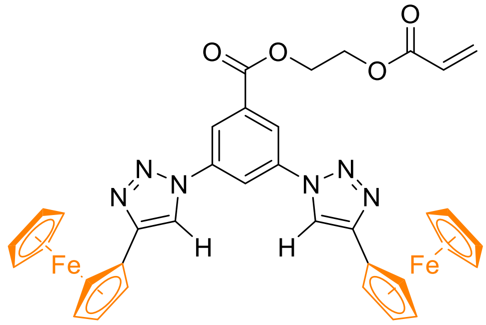

1·HB

$[Cu(CH_3CN)_4]PF_6$ (99 mg, 0.266 mmol) and tris((1-benzyl-4-triazolyl)methyl)amine (TBTA, 132 mg, 0.266 mmol) were dissolved in DCM (anhydrous, 2 mL). The mixture was stirred for 30 min under N_2. Then ethynylferrocene, **3·HB** (209 mg, 1.00 mmol) and **2** (150 mg, 0.500 mmol) were added. The reaction mixture was protected from light and stirred overnight under N_2. Next, it was washed with aqueous EDTA (10%)/NH_3 (2%) solution (3 x 20 mL), followed by brine (20 mL). The organic fractions were combined and dried over $MgSO_4$. The solvent was removed *in vacuo* to give the crude product. Purification by flash chromatography (SiO_2, DCM/MeOH 99:1) afforded product **1·HB** as a crystalline orange solid (320 mg, 56% yield).

^{1}H NMR (400 MHz, $CDCl_3$), δ: 8.64 (t, J = 2.02 Hz, 1H), 8.48 (d, J = 2.02 Hz, 2H), 8.10 (s, 2H), 6.49 (dd, 1H), 6.20 (dd, 1H) 5.90 (dd, 1H), 4.84 (t, J = 1.83 Hz, 4H) 4.69 (m, 2H), 4.61 (m, 2H), 4.39 (t, J = 1.83 Hz, 4H), 4.14 (s, 10H).

^{13}C NMR (151 MHz, $CDCl_3$), δ: 166.02, 164.15, 148.86, 138.27, 133.20, 131.86, 127.95, 119.99, 117.40, 116.12, 71.35, 70.44, 68.08, 64.05, 61.99.

HRMS (ESI+ve) m/z: 723.1077 ($[M+H]^+$, $C_{36}H_{31}Fe_2N_6O_4$ requires 723.1100)

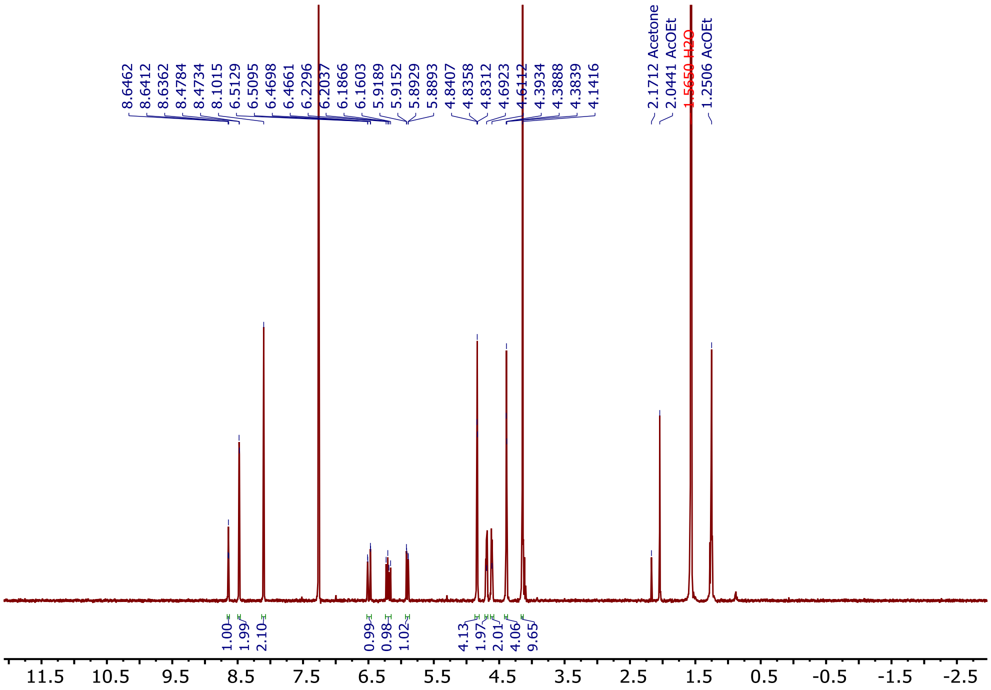

Figure S2.8. ^{1}H NMR spectrum of **1·HB** ($CDCl_3$, 298K, 400 MHz).

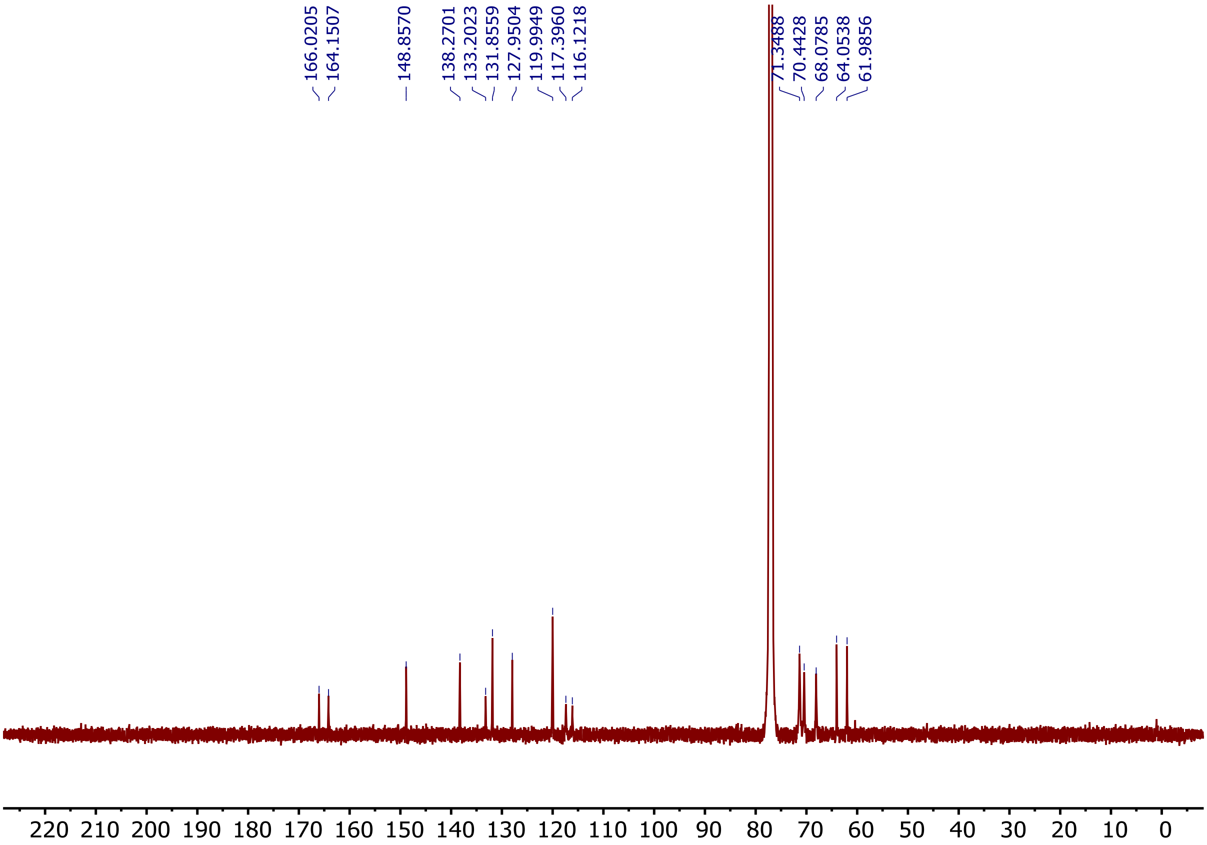

Figure S2.9. ^{13}C NMR spectrum of **1·HB** in ($CDCl_3$, 298K, 151 MHz).

S2.2 Synthesis of Polymeric Hosts

Polymerisation Procedure

All RAFT polymerisation reactions were performed in microwave vials which were filled with 0.5 mL of the appropriate polymerisation solution (molar ratio of 200:1:0.2 [M_{total}]:[DDMAT]:[AIBN] in DMF), and subsequently sealed with aluminium seals with PTFE septa to ensure airtight conditions. Each vial was then thoroughly degassed with Ar through the polymerisation solution for 15 min, then placed in an oil bath at 70 °C to initiate polymerisation. Polymerisation reactions were left to continue for at least 24 h, and reaction progress was monitored with aliquots taken periodically with a degassed (Ar) syringe and needle. Unless stated otherwise, all polymers were purified by size exclusion with a column filled with Bio-Beads™ S-X1 Support (styrene divinylbenzene beads, 1% crosslinkage, 40-80 µm bead size, 600-14,000 MW exclusion range) and $CHCl_3$ as an eluent.

Homopolymer pDEGA was purified by removing the residual DMF *in vacuo* to afford the crude polymer, which was resolubilised in a small amount (≈0.5 mL) THF, and precipitated in cold hexane (≈5 mL). The resulting suspension was then centrifuged at 3900 RPM for 12 min, from which the supernatant was removed and the precipitate resolubilised in a small amount of THF. This washing procedure was repeated three times in total, before removing residual solvent *in vacuo* to obtain the pure pDEGA polymer.

pDEGA-1·XB vs. pDEGA-1·HB Polymerisation Comparisons

Any discrepancies between the physical characteristics (e.g. M_n) of the polymeric hosts were attributed to the slightly higher difficulty of copolymerising **1·XB** over **1·HB**, which was hypothesised to be due to the weak C-I bond interfering with the radical polymerisation mechanism. It was observed that copolymerisation of **1·XB** required significantly longer reaction times than **1·HB** (≈3 d vs. 1 d, respectively) to obtain similar chain lengths.

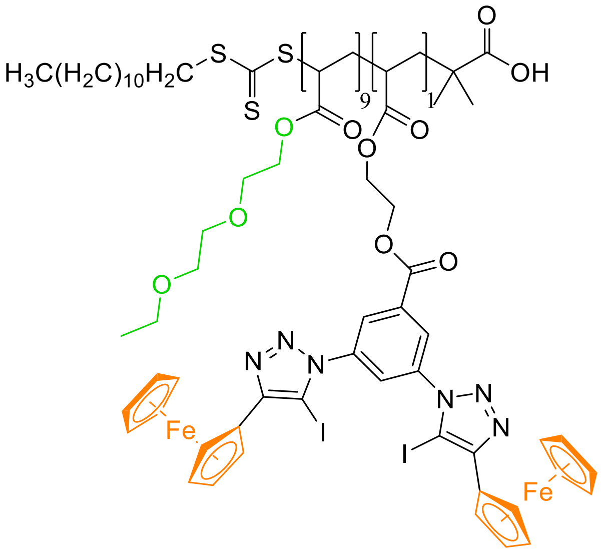

pDEGA-1·XB

^{1}H NMR (400 MHz, $CDCl_3$), δ: 8.52 (bs, 2H), 8.13 (bs, 1H), 5.10 (bs, 4H) 4.62 (bs, 2H), 4.39 (bs, 6H), 4.19 (bs, 28H), 3.72-3.44 (bs, 72H), 2.32 (bs, polymer backbone), 1.91 (bs, polymer backbone), 1,62 (bs, polymer backbone), 1.20 (bt, 27H)

^{13}C NMR (151 MHz, $CDCl_3$), δ: 174.31, 70.65, 69.90, 68.98, 66.70, 63.57, 41.28, 15.27.

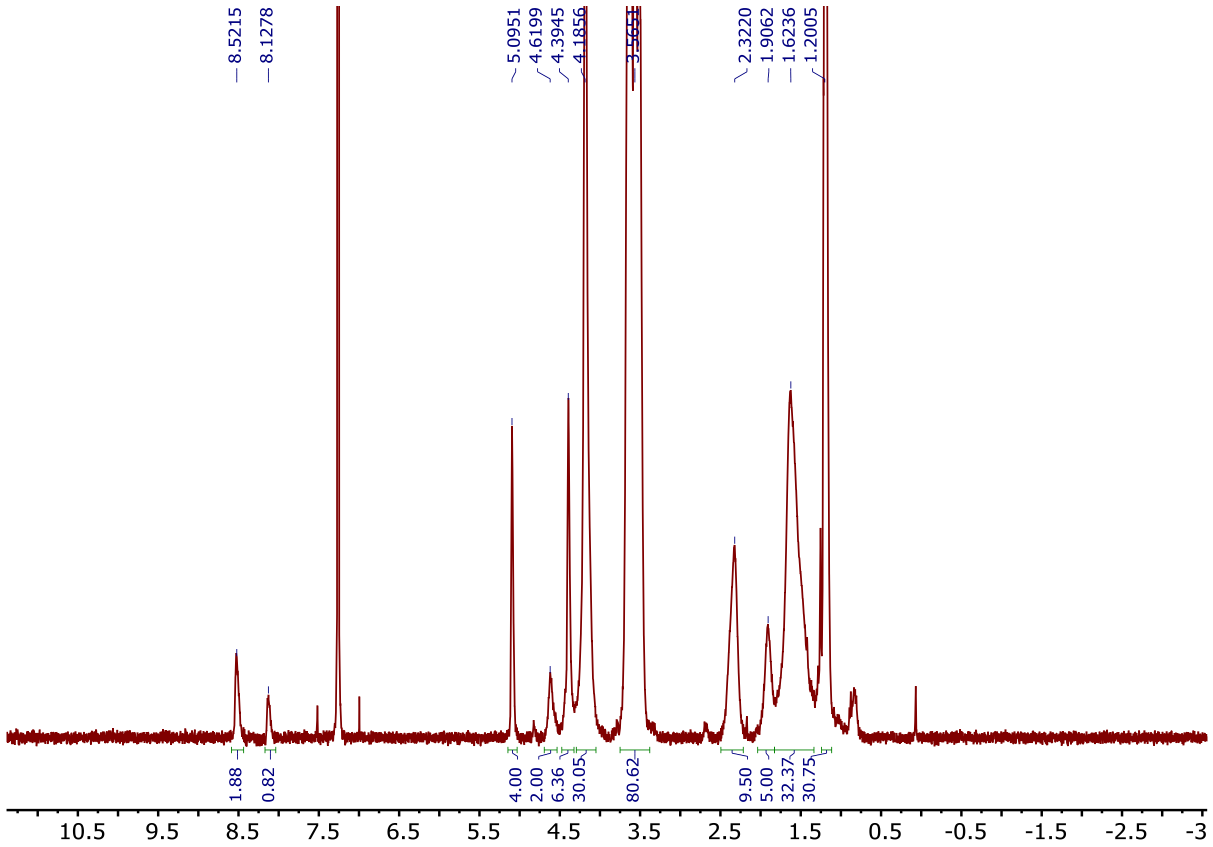

Figure S2.10. ^{1}H NMR spectrum of **pDEGA-1·XB** ($CDCl_3$, 298K, 400 MHz).

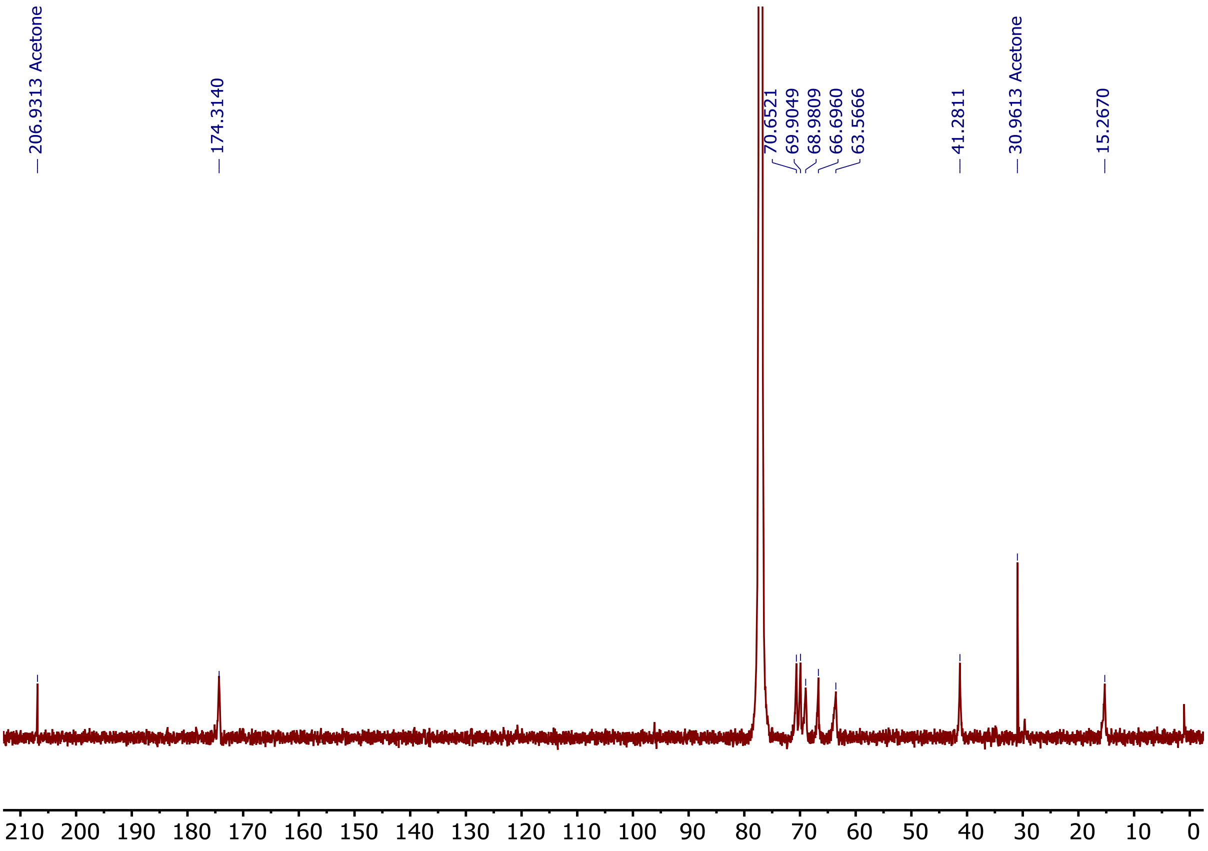

Figure S2.11. ^{13}C NMR spectrum of **pDEGA-1·XB** ($CDCl_3$, 298K, 151 MHz).

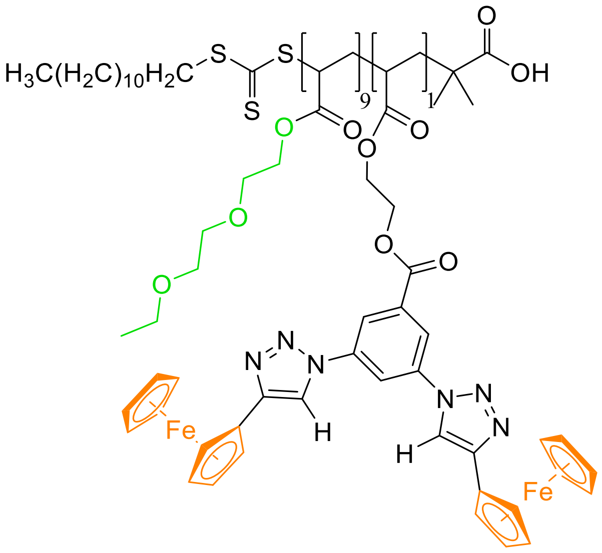

pDEGA-1·HB

^1H NMR (400 MHz, $CDCl_3$), δ: 8.72 (bs, 1H), 8.50 (bs, 2H), 8.30 (bs, 2H), 4.82 (bs, 4H) 4.64 (bs, 2H), 4.46 (bs, 2H), 4.35 (bs, 4H), 4.18 (bs, 10H), 4.11 (bs, 18H) 3.73-3.43 (bs, 72H), 2.32 (bs, polymer backbone), 1.89 (bs, polymer backbone), 1.19 (bt, 27H)
^{13}C NMR (151 MHz, $CDCl_3$), δ: 174.25, 70.57, 69.87, 68.88, 66.62, 63.49, 41.28, 15.25.

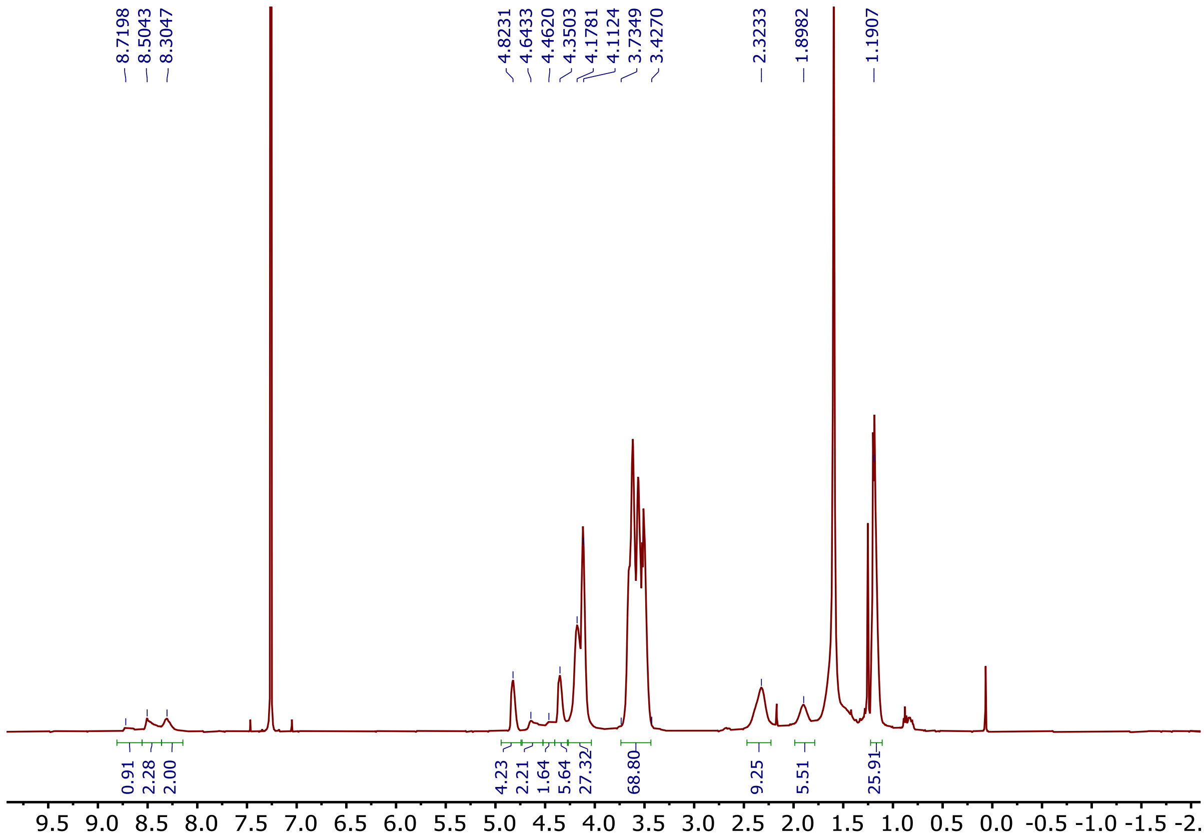

Figure S2.12. ^{1}H NMR spectrum of **pDEGA-1·HB** ($CDCl_3$, 298K, 400 MHz).

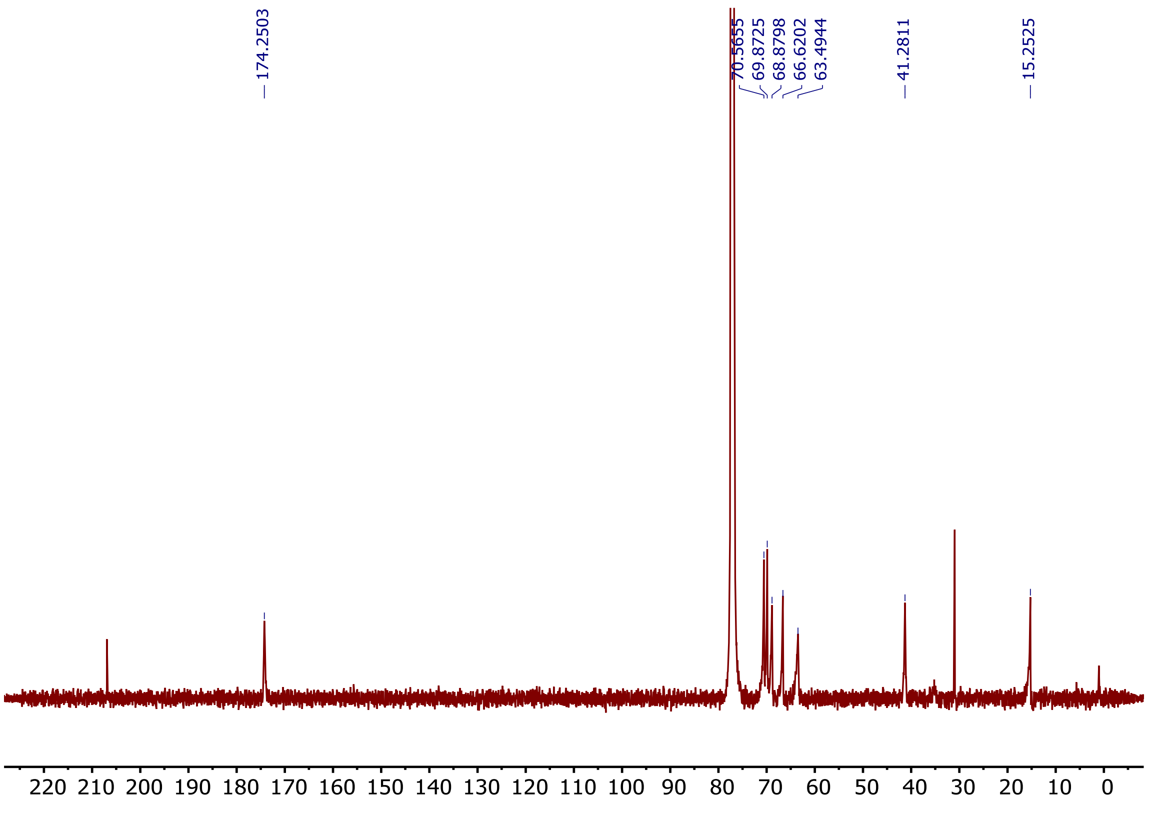

Figure S2.13. ^{13}C NMR spectrum of **pDEGA-1·HB** ($CDCl_3$, 298K, 151 MHz).

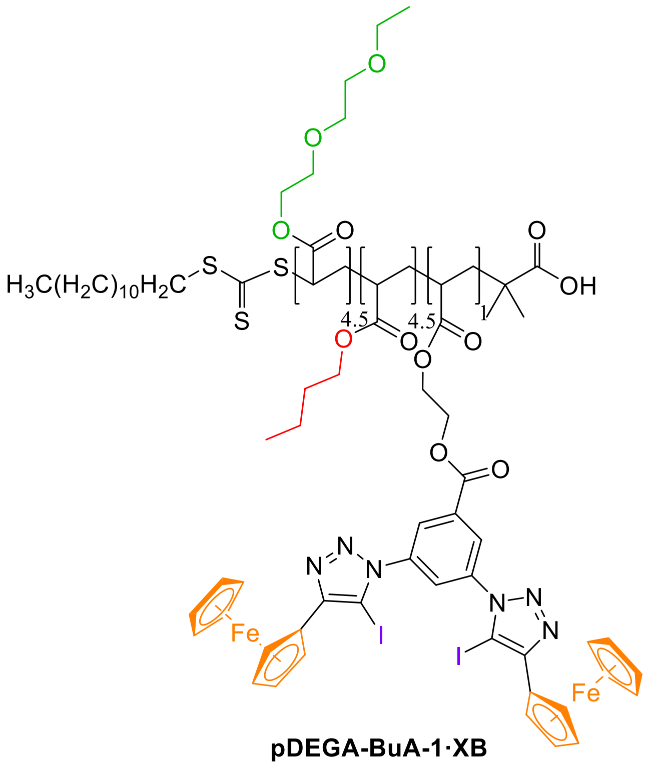

pDEGA-BuA-1·XB

^{1}H NMR (400 MHz, $CDCl_3$), δ: 8.52 (bs, 2H), 8.14 (bs, 1H), 5.09 (bs, 4H) 4.62 (bs, 2H), 4.39 (bs, 6H), 4.24 (bt, 9H) 4.18 (bs, 10H), 4.03 (bt, 9H) 3.71-3.44 (bs, 36H), 2.32 (bs, polymer backbone), 1.90 (bs, polymer backbone), 1.36 (bs, polymer backbone), 1.20 (bs, polymer backbone), 0.93 (bs, 13.5H), 0.83 (bs, 9H)

^{13}C NMR (151 MHz, $CDCl_3$), δ: 174.32, 70.64, 69.90, 68.92, 66.71, 64.50, 63.65, 41.36, 30.65, 19.14, 15.29, 13.81.

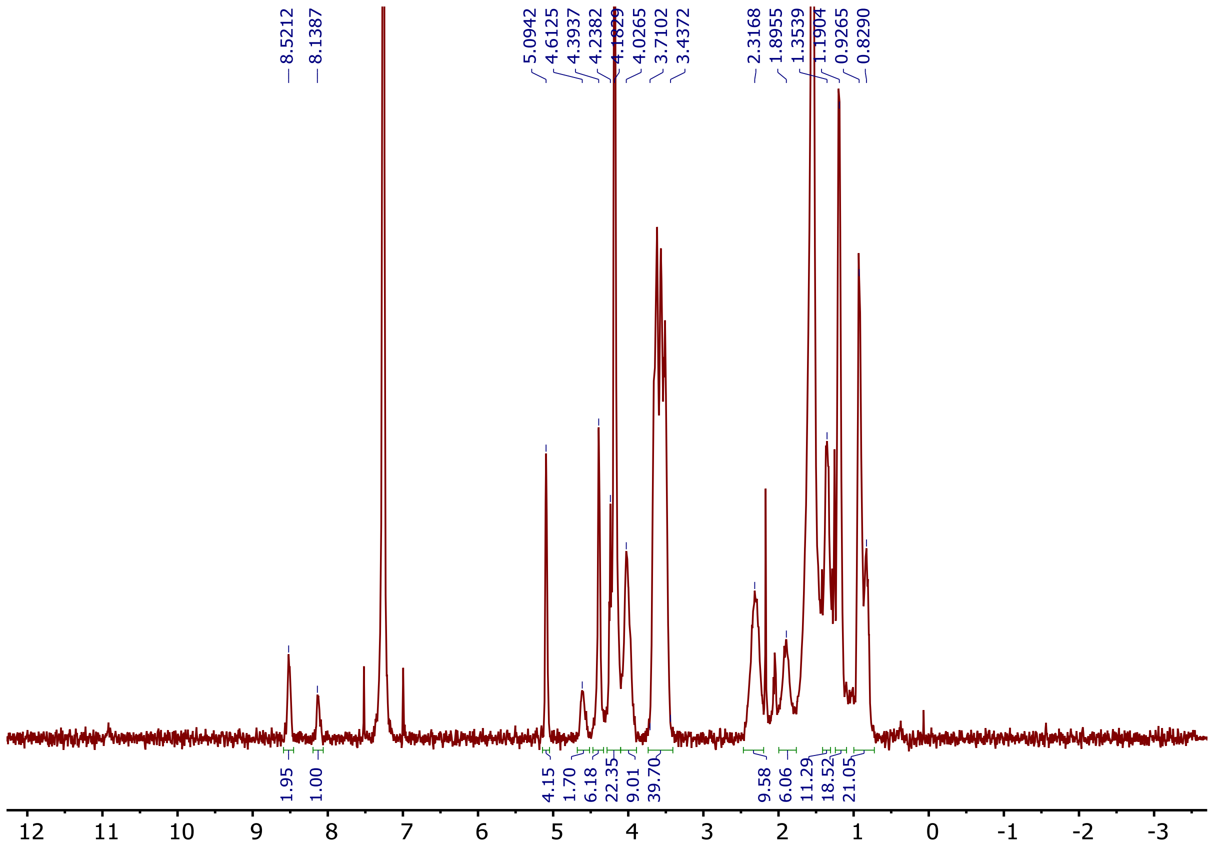

Figure S2.14. ^{1}H NMR spectrum of **pDEGA-BuA-1·XB** ($CDCl_3$, 298K, 400 MHz).

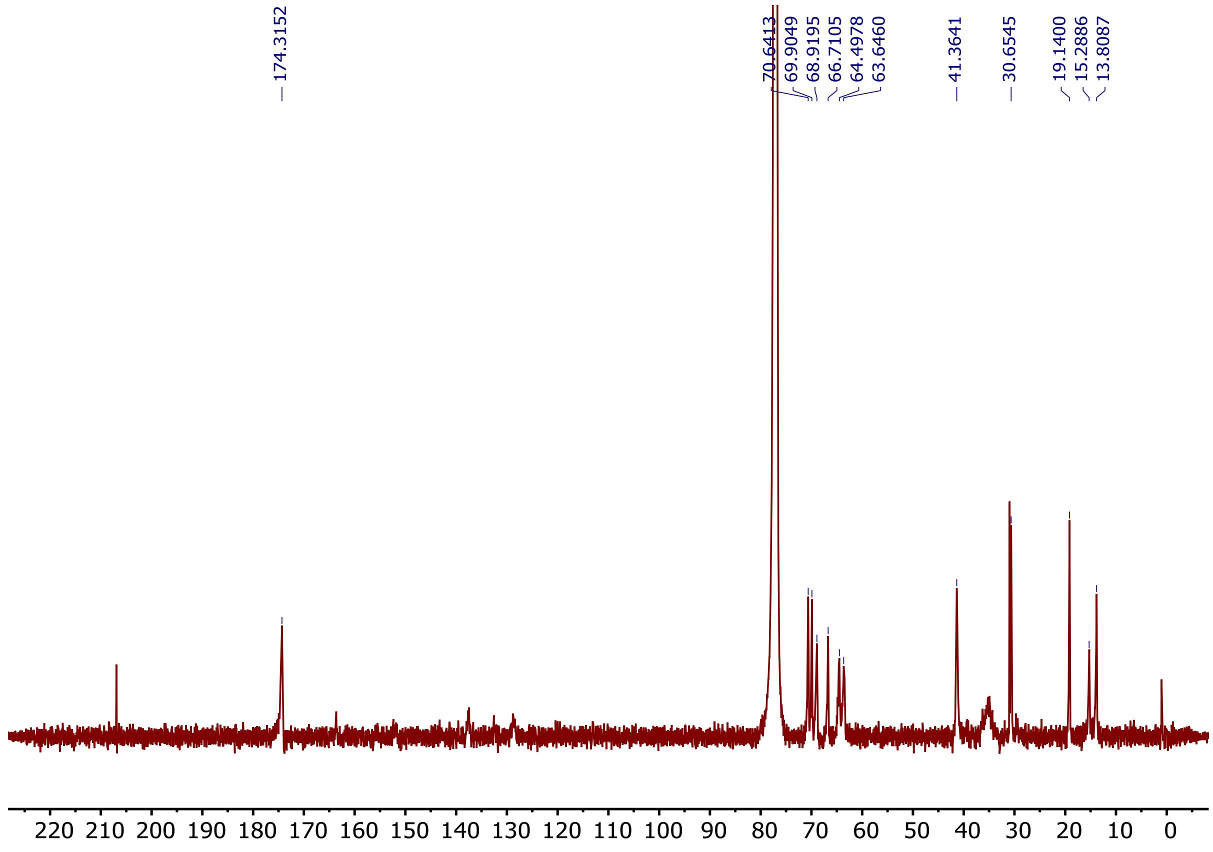

Figure S2.15. ^{13}C NMR spectrum of **pDEGA-BuA-1·XB** ($CDCl_3$, 298K, 151 MHz).

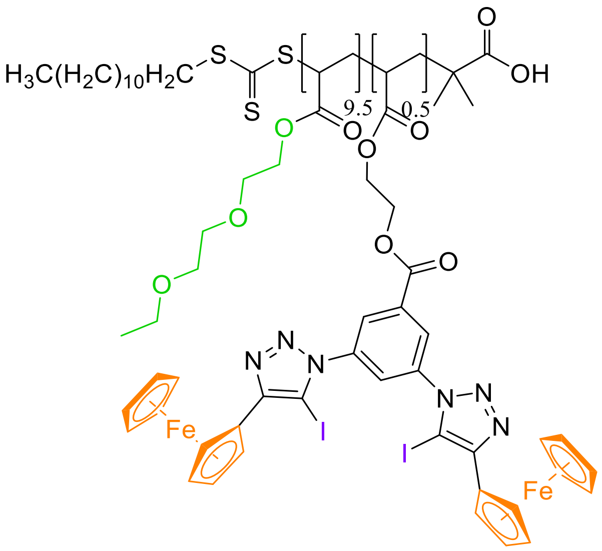

pDEGA-1·XB$_{0.5}$

^{1}H NMR (400 MHz, CDCl$_3$), δ: 8.53 (bs, 2H), 8.14 (bs, 1H), 5.10 (bs, 4H) 4.62 (bs, 2H), 4.34 (bs, 6H), 4.19 (bs, 38H), 3.72-3.44 (bs, 152H), 2.33 (bs, polymer backbone), 1.91 (bs, polymer backbone), 1,64 (bs, polymer backbone), 1.20 (bt, 57H)

^{13}C NMR (151 MHz, CDCl$_3$), δ: 174.32, 70.61, 69.91, 68.94, 66.67, 63.54, 41.31, 15.28.

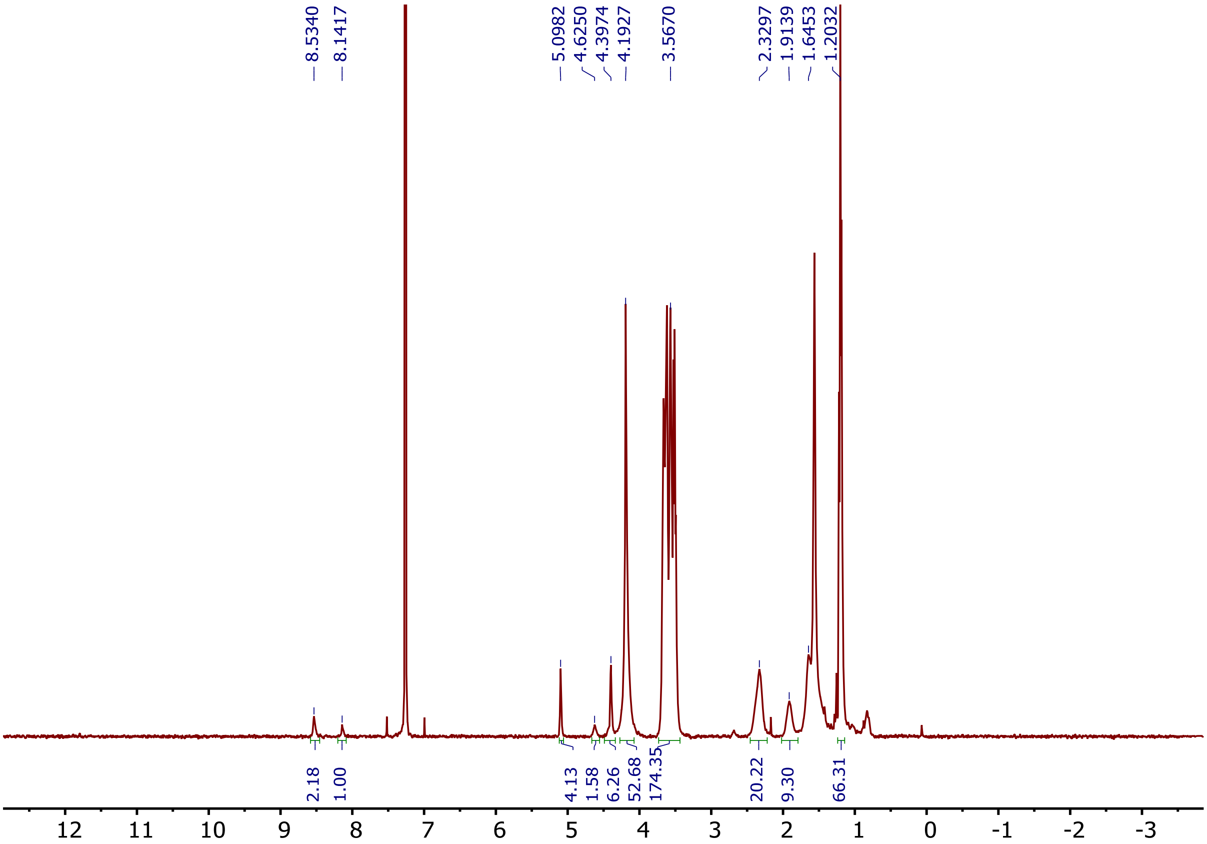

Figure S2.16. ^{1}H NMR spectrum of **pDEGA-1·XB$_{0.5}$** (CDCl$_3$, 298K, 400 MHz).

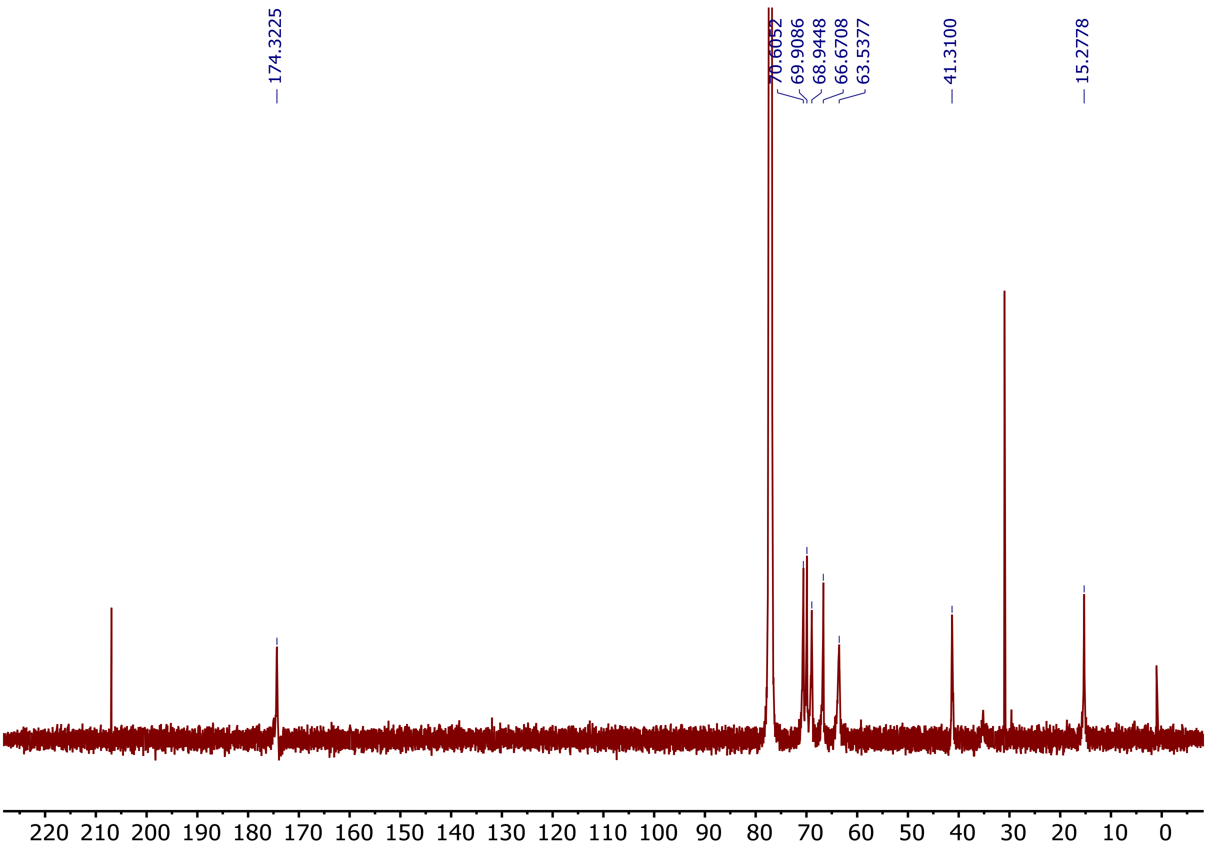

Figure S2.17. ^{13}C NMR spectrum of **pDEGA-1·XB** ($CDCl_3$, 298K, 151 MHz).

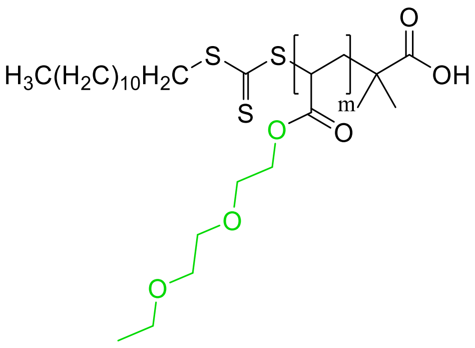

pDEGA

^{1}H NMR (600 MHz, $CDCl_3$), δ: 4.20 (bs, 2H), 3.67 (bs, 2H), 3.63 (bs, 2H), 3.57 (bs, 2H), 3.52 (q, 2H) 2.33 (bs, polymer backbone), 1.92 (bs, polymer backbone), 1.70 (bs, polymer backbone), 1.65 (bs, polymer backbone), 1.5 (bs), 1.20 (t, 3H)
^{13}C NMR (151 MHz, $CDCl_3$), δ: 174.39, 70.68, 69.99, 69.00, 66.74, 63.73, 63.56, 41.41, 15.3.

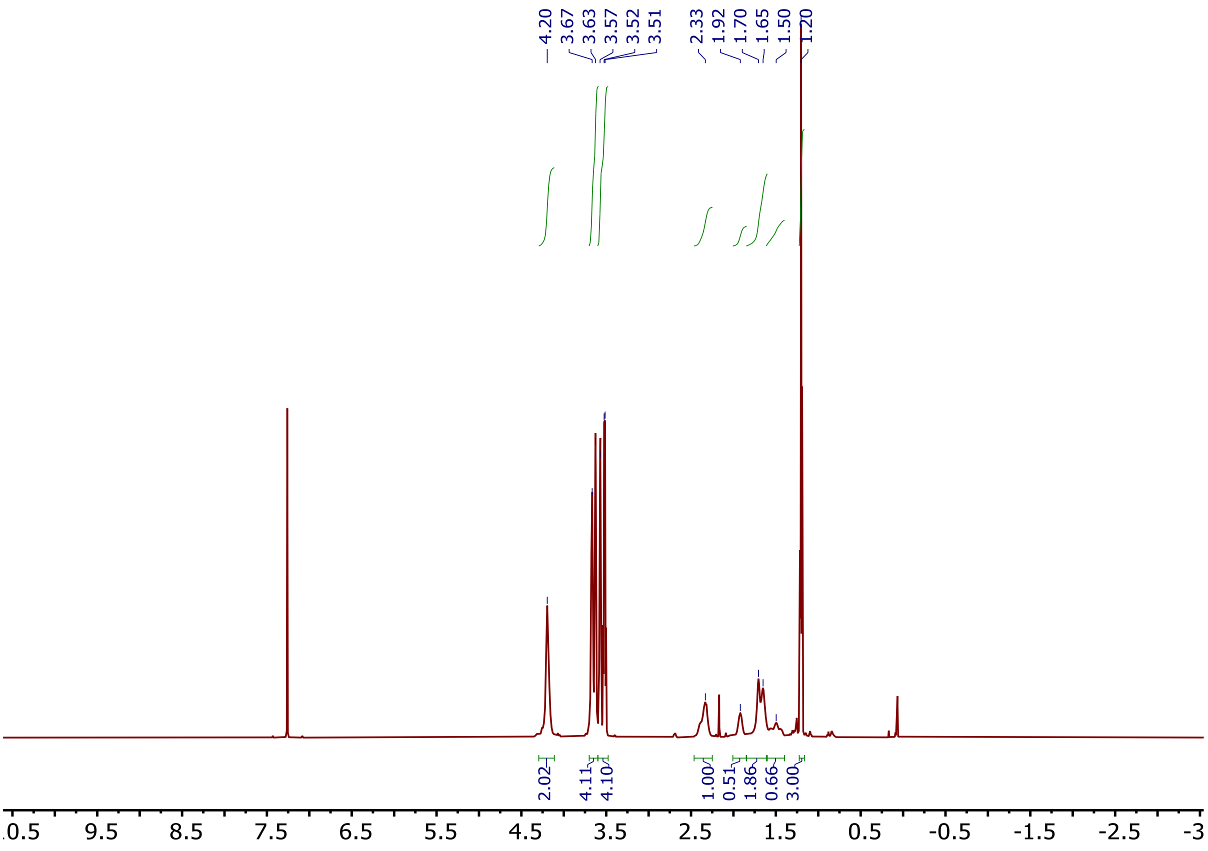

Figure S2.18. ^{1}H NMR spectrum of **pDEGA** ($CDCl_3$, 298K, 600 MHz).

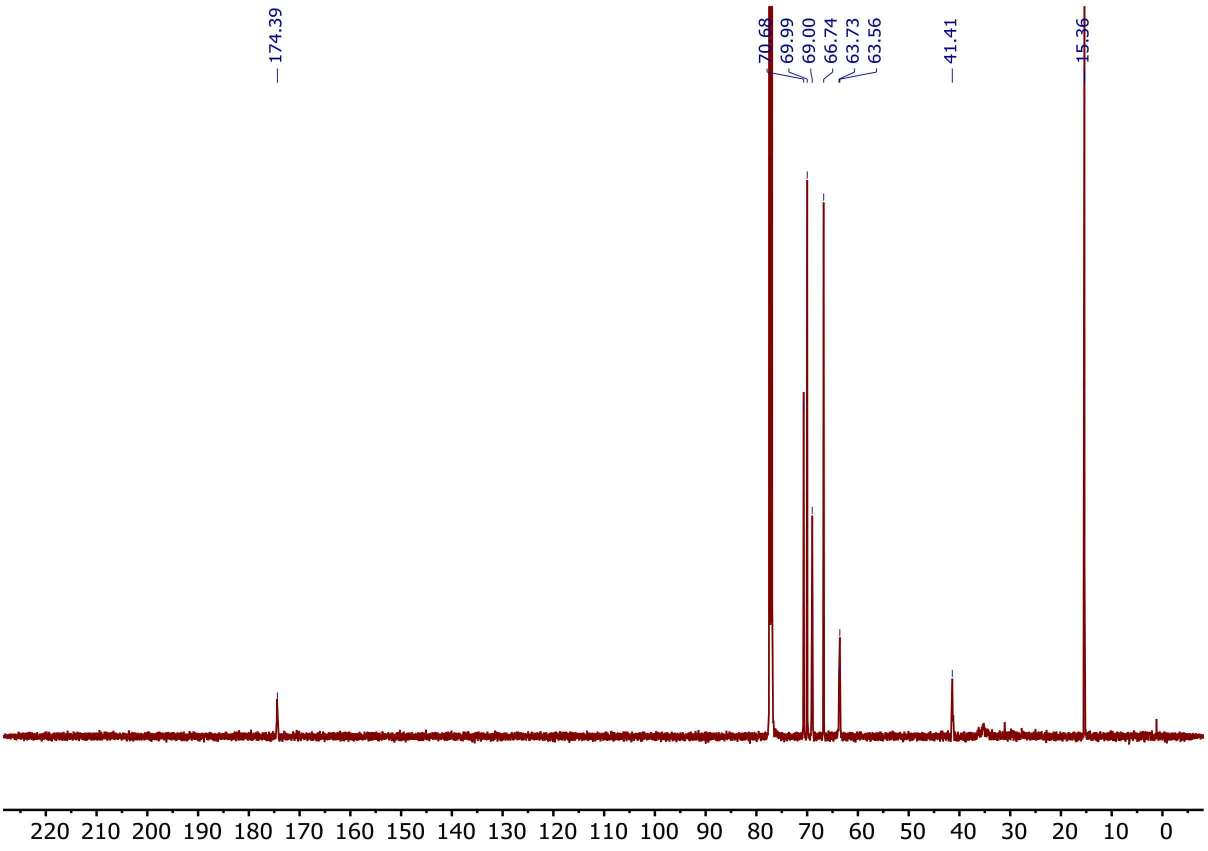

Figure S2.19. ^{13}C NMR spectrum of **pDEGA** ($CDCl_3$, 298K, 151 MHz).

S3 Polymer Characterisation

S3.1 ATR-FTIR Characterisation

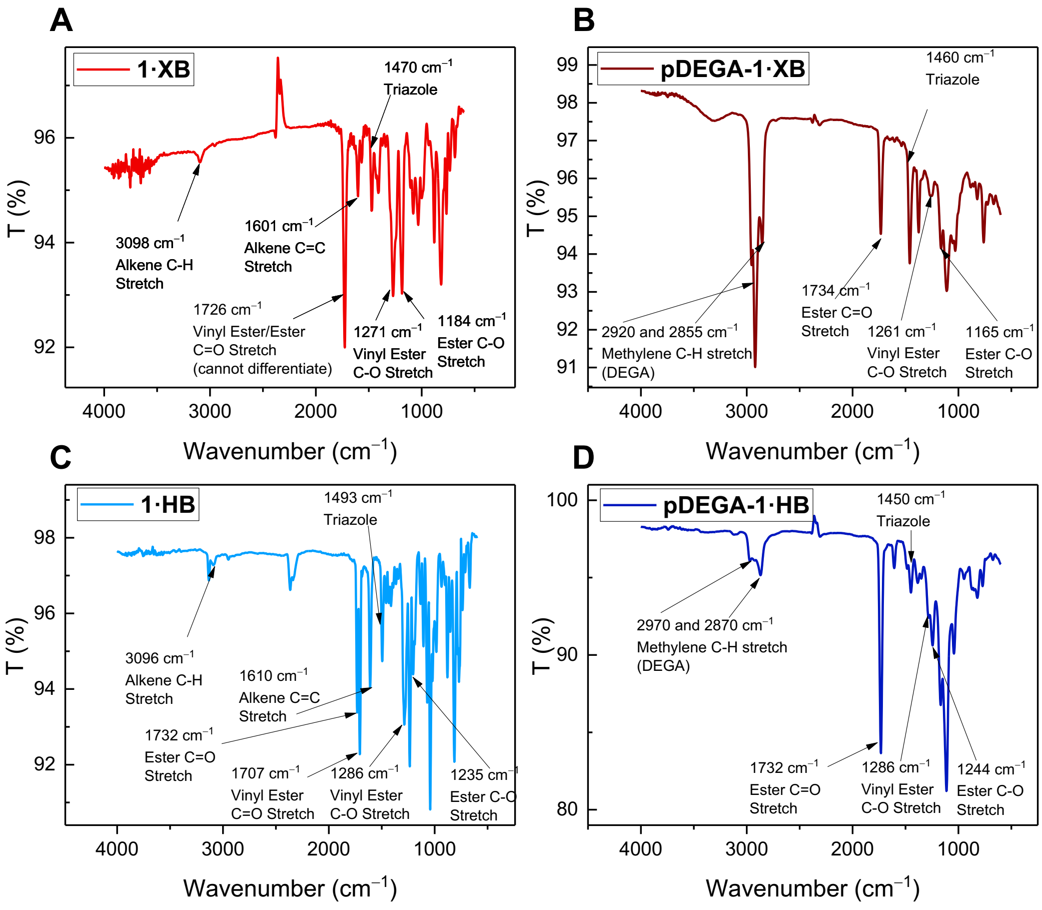

Figure S3.1. ATR-FTIR spectra of **1·XB/HB** and **pDEGA-1·XB/HB**.

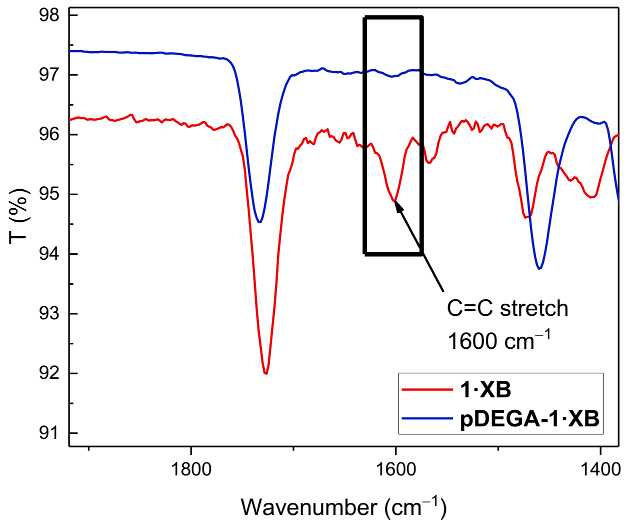

Figure S3.2. ATR-FTIR spectra of **1·XB** and **pDEGA-1·XB**, highlighting the loss of the C=C stretch signal at 1600 cm^{-1}, attributed to polymerisation of the acrylate functionality from **1·XB** into the **pDEGA-1·XB** polymer backbone.

S3.2 GPC Data

Table S3.1. Physical characterisation data of all polymeric hosts and control non-receptive polymer pDEGA including M_n and PDI obtained by GPC, and associated information about the composition of each polymeric host.

Host	M_n (kDa)	PDI	Average Total no. Units	Average No. Receptive Units	Yield
**pDEGA-1·XB**	15.7	1.37	57	5.7	47%
**pDEGA-1·HB**	27.7	1.38	115	11.5	53%
**pDEGA-1·XB$_{0.5}$**	12.2	1.40	52	2.6	68%
**pDEGA-BuA-1·XB**	16.7	1.40	70	7.0	48%
**pDEGA**	32.0	1.15	168	N.A.	67%

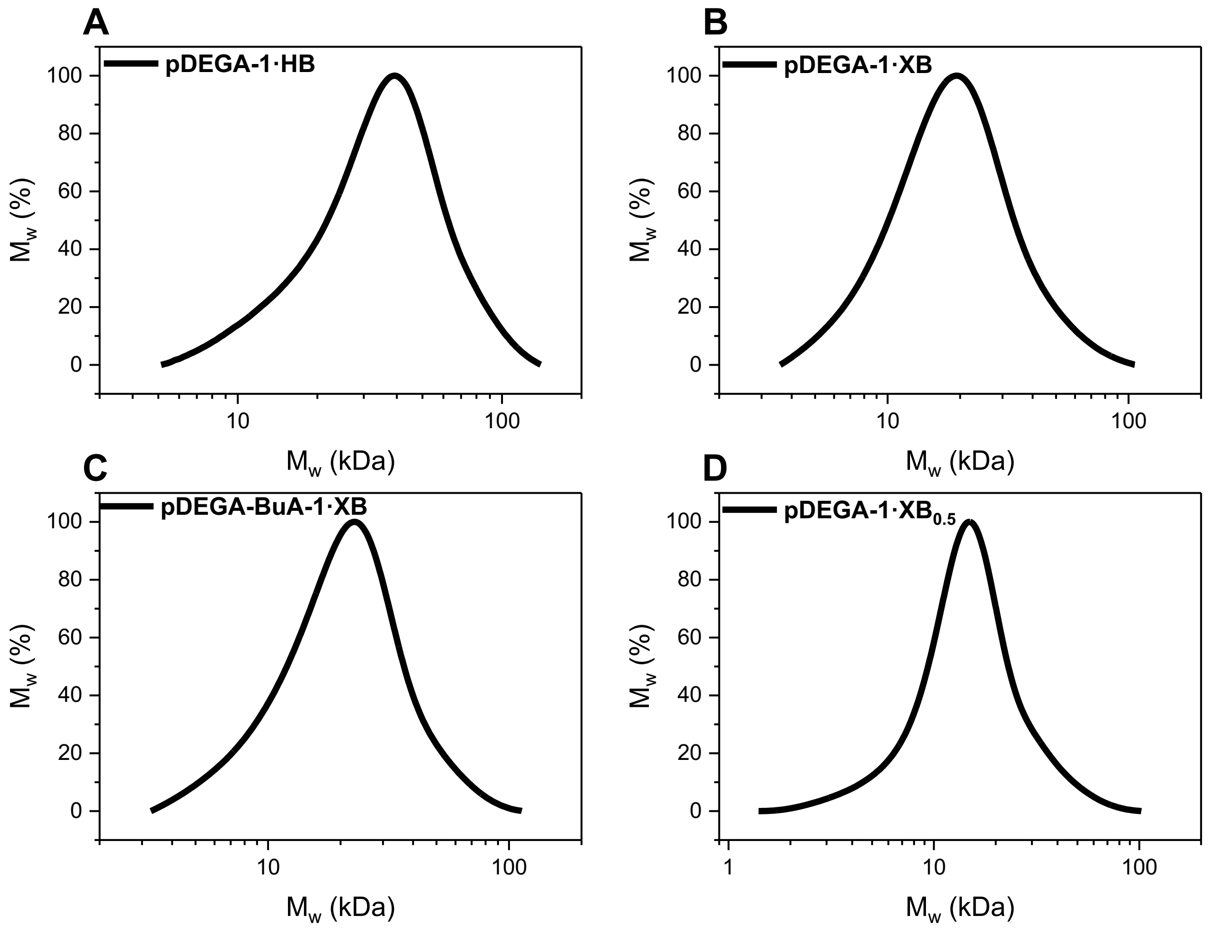

Figure S3.3. GPC data for all receptive co-polymers A) **pDEGA-1·HB,** B) **pDEGA-1·XB,** C) **pDEGA-BuA-1·XB** and D) **pDEGA-1·XB$_{0.5}$**.

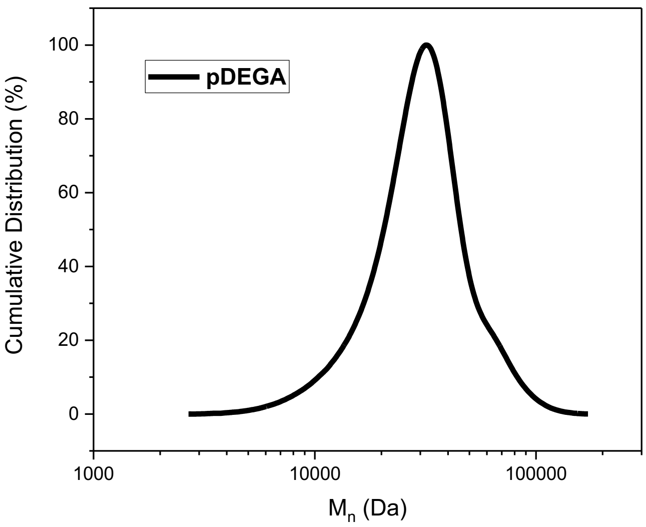

Figure S3.4. GPC data for non-receptive homopolymer **pDEGA**.

S4 Anion Binding Studies of Monomeric Hosts 1·XB/HB by ^{1}H NMR

A 50 mM solution of the chosen TBA salt of an anion (TBAX, X = I^-, Br^-, Cl^- or $H_2PO_4^-$) was added to 0.5 mL solution containing 1 mM of receptor **1·XB**/**HB** in the chosen solvent system:

a) acetone-d_6:D_2O 97.5:2.5, v/v;
b) acetone-d_6:acetonitrile-d_3:D_2O 48.75:48.75:2.5, v/v/v;
c) or acetonitrile-d_3:D_2O 97.5:2.5, v/v.

Each titration isotherm comprises 17 data points, corresponding to 0.0, 0.2, 0.4, 0.6, 0.8, 1.0, 1.2, 1.4, 1.6, 1.8, 2, 2.5, 3.0, 4.0, 5.0, 7.0, 10.0 equivalents of added guest anion (100 µl of guest solution added by the last point). Addition of the solution containing the guest caused dilution of the host, which was accounted for in calculations. Binding constants were determined using BindFit, applying a 1:1 host-guest binding model in all cases. At least two signals were simultaneously used to determine binding constants (global fitting approach – binding constant used a shared parameter). Fitting using 2:1 host-guest model failed in all cases. Only signals with high perturbation were used for determining binding constants: protons *a* & *b* in the case of **1·XB** and *b* & *d* in the case of **1·HB**.

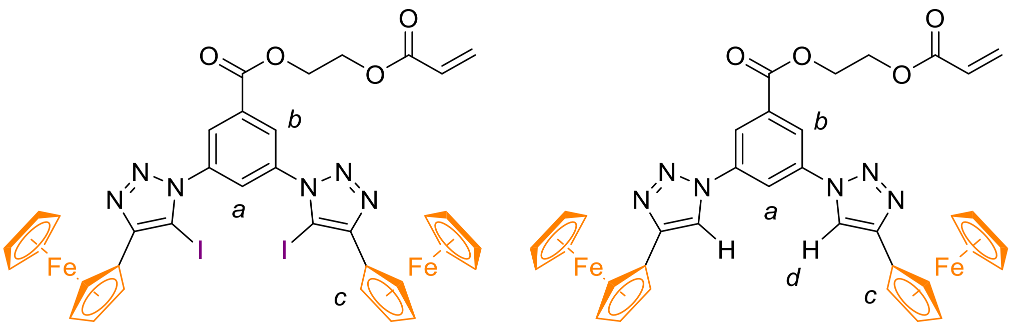

1·XB **1·HB**

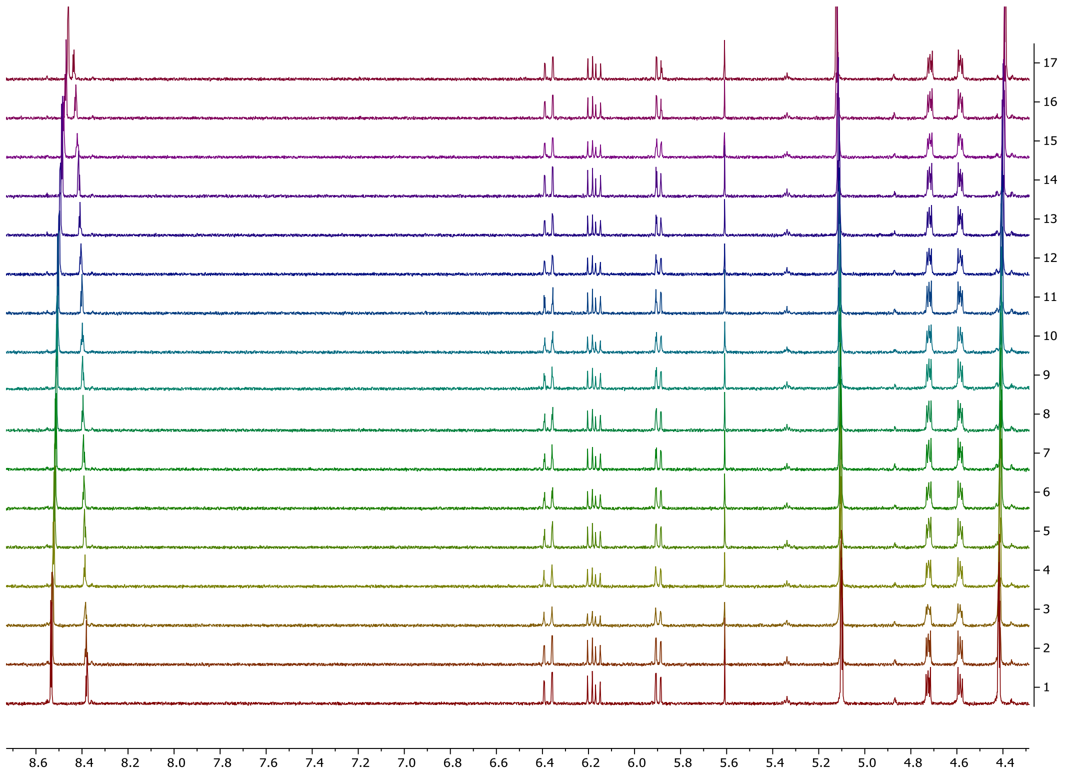

Figure S4.1. Stacked ^{1}H NMR spectra from titration of **1·XB** with TBACl in acetone-d_6:D_2O (97.5:2.5, v/v), 500 MHz, 298 K. Spectrum no. 1 – 0 equivalents of the guest; spectrum no. 17 – 10 equivalents of the guest.

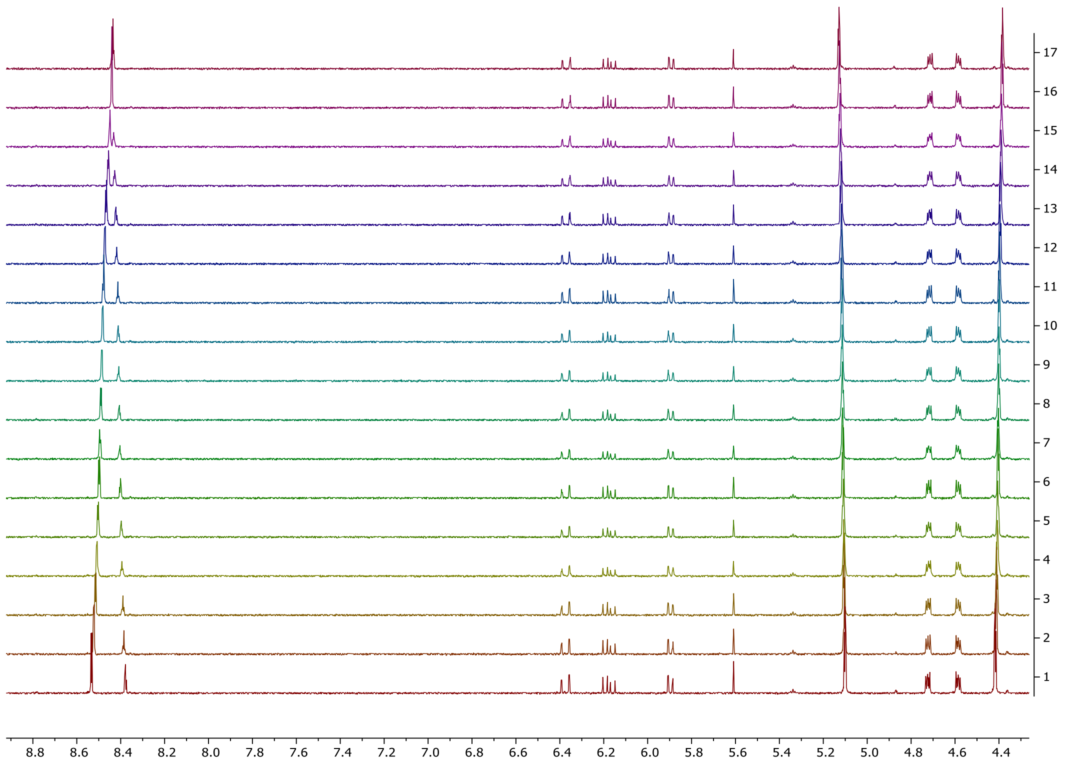

Figure S4.2. Stacked ^{1}H NMR spectra from titration of **1·XB** with TBABr in acetone-d_6:D_2O (97.5:2.5, v/v), 500 MHz, 298 K. Spectrum no. 1 – 0 equivalents of the guest; spectrum no. 17 – 10 equivalents of the guest.

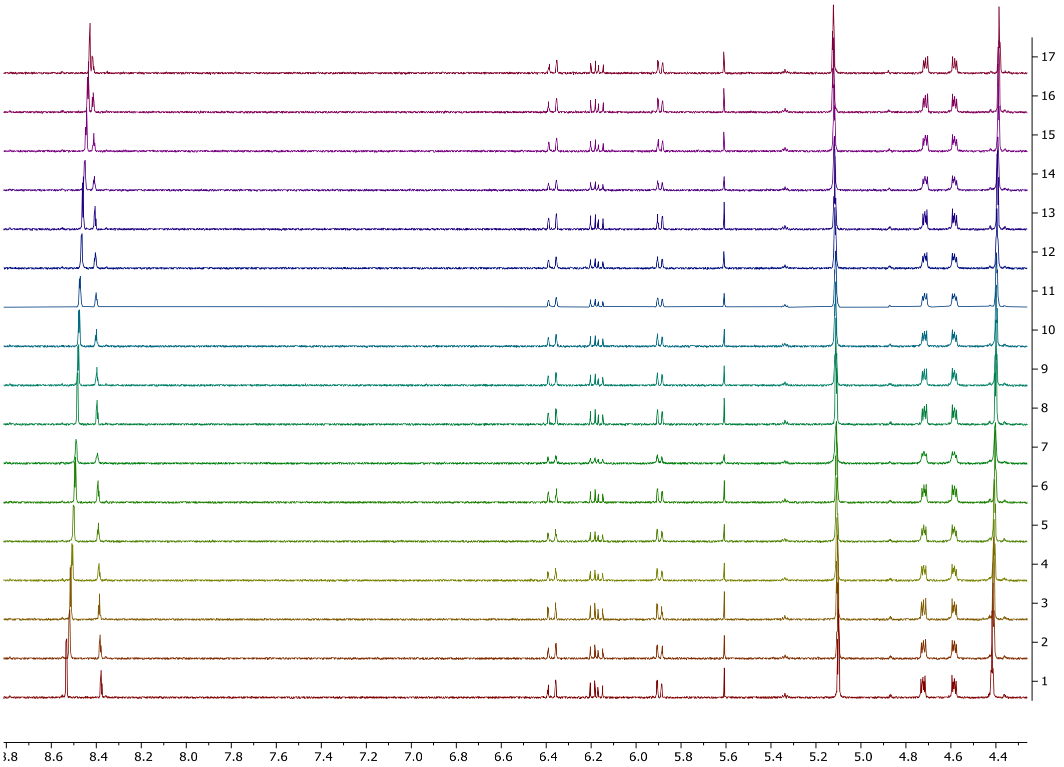

Figure S4.3. Stacked ^{1}H NMR spectra from titration of **1·XB** with TBAI in acetone-d_6:D_2O (97.5:2.5, v/v), 500 MHz, 298 K. Spectrum no. 1 – 0 equivalents of the guest; spectrum no. 17 – 10 equivalents of the guest.

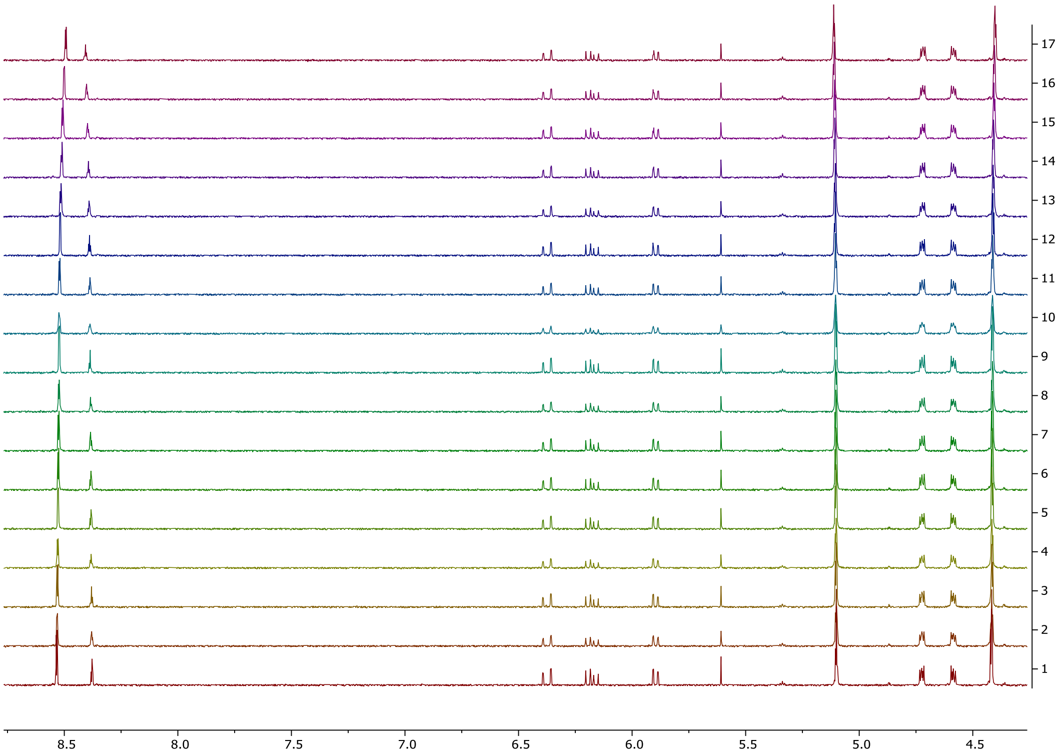

Figure S4.4. Stacked ^{1}H NMR spectra from titration of **1·XB** with $TBAH_2PO_4$ in acetone-d_6:D_2O (97.5:2.5, v/v), 500 MHz, 298 K. Spectrum no. 1 – 0 equivalents of the guest; spectrum no. 17 – 10 equivalents of the guest.

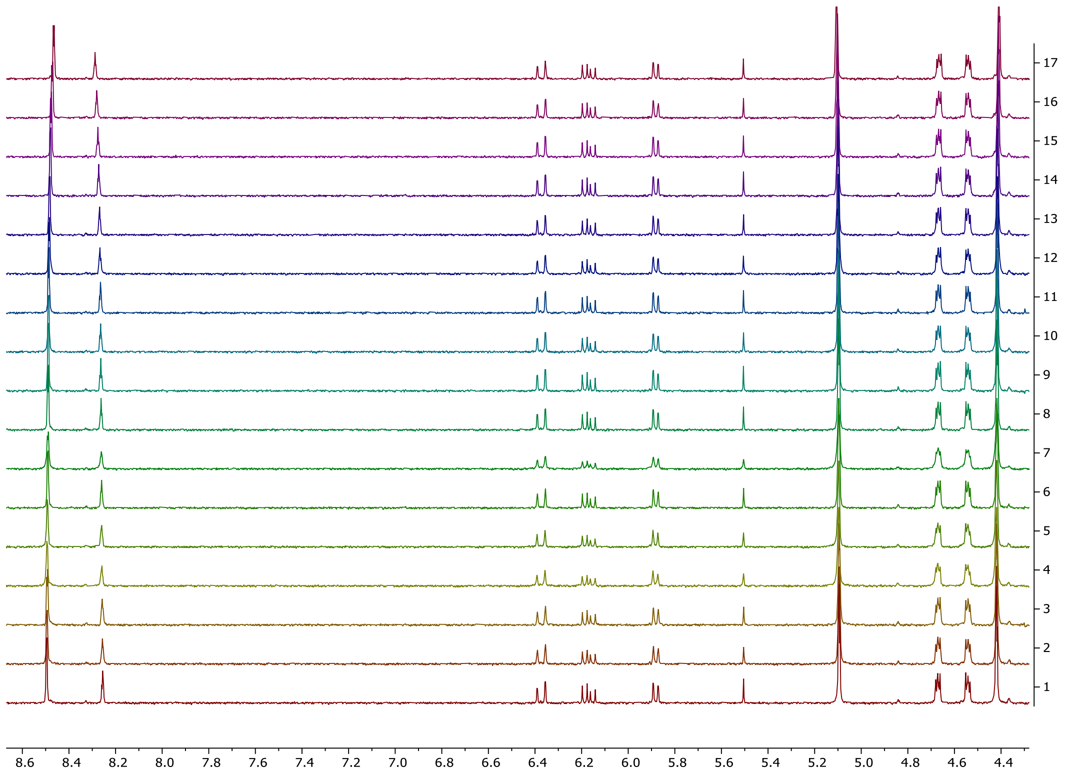

Figure S4.5. Stacked ^{1}H NMR spectra from titration of **1·XB** with TBACl in acetone-d_6:acetonitrile-d_3:D_2O (48.75:48.75:2.5, v/v/v), 500 MHz, 298 K. Spectrum no. 1 – 0 equivalents of the guest; spectrum no. 17 – 10 equivalents of the guest.

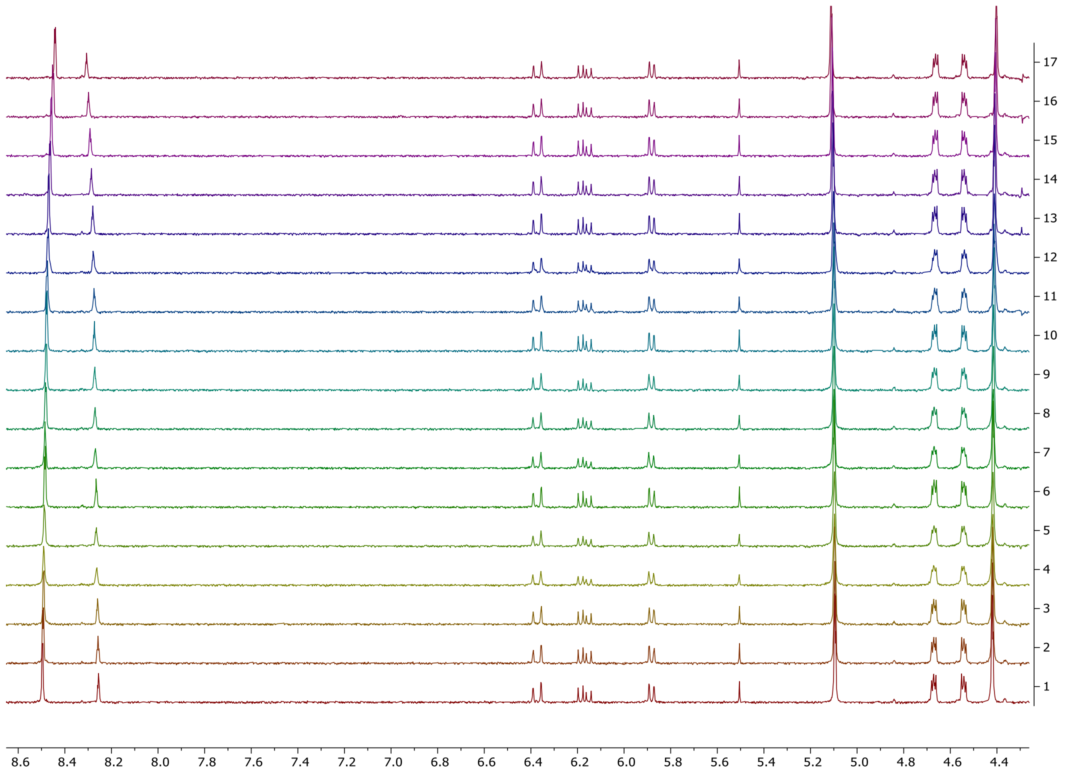

Figure S4.6. Stacked ^{1}H NMR spectra from titration of **1·XB** with TBABr in acetone-d_6:acetonitrile-d_3:D_2O (48.75:48.75:2.5, v/v/v), 500 MHz, 298 K. Spectrum no. 1 – 0 equivalents of the guest; spectrum no. 17 – 10 equivalents of the guest.

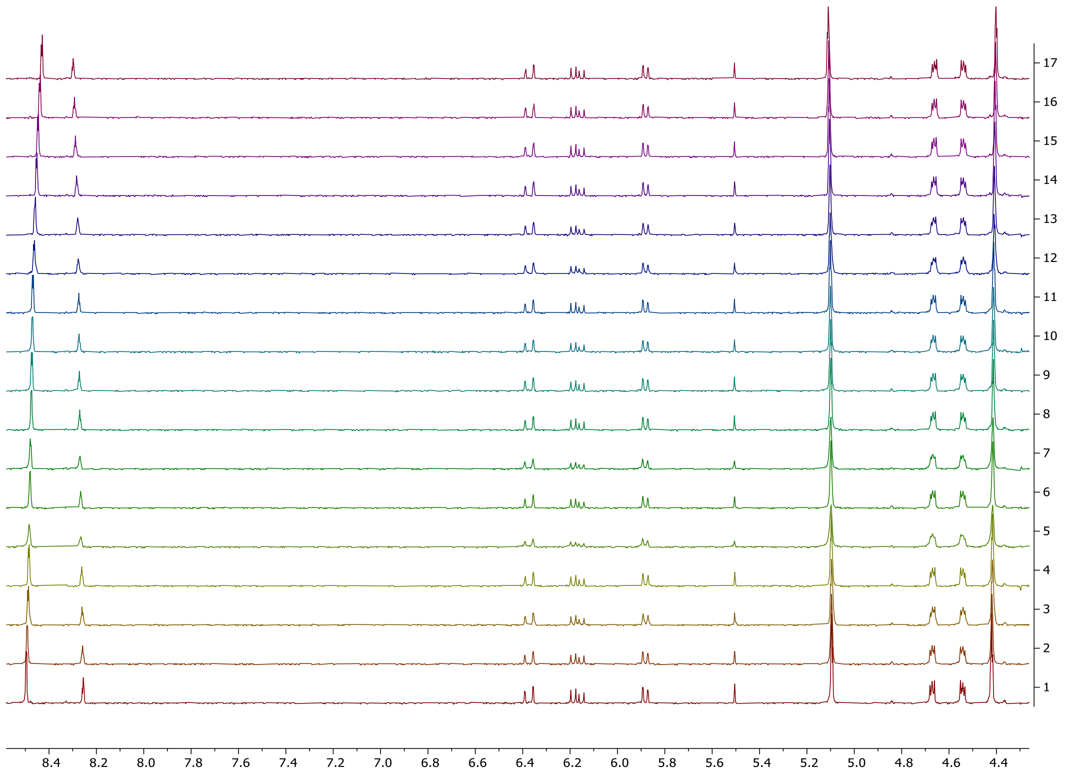

Figure S4.7. Stacked ^{1}H NMR spectra from titration of **1·XB** with TBAI in acetone-d_6:acetonitrile-d_3:D_2O (48.75:48.75:2.5, v/v/v), 500 MHz, 298 K. Spectrum no. 1 – 0 equivalents of the guest; spectrum no. 17 – 10 equivalents of the guest.

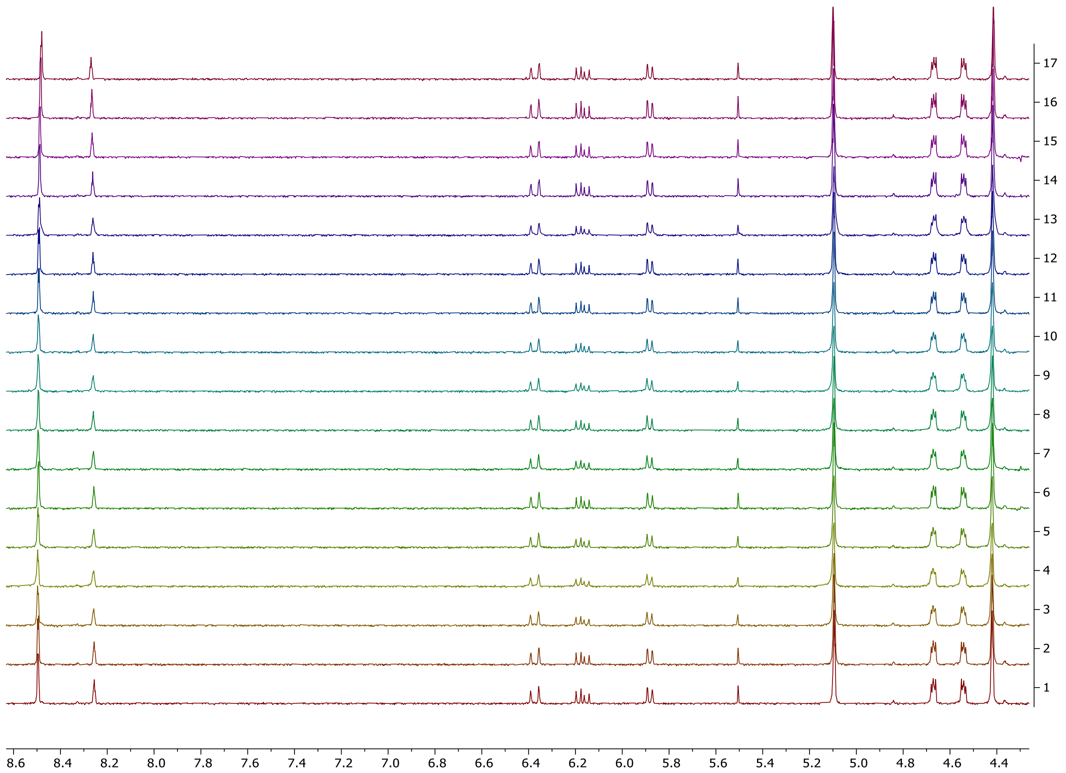

Figure S4.8. Stacked ^{1}H NMR spectra from titration of **1·XB** with $TBAH_2PO_4$ in acetone-d_6:acetonitrile-d_3:D_2O (48.75:48.75:2.5, v/v/v), 500 MHz, 298 K. Spectrum no. 1 – 0 equivalents of the guest; spectrum no. 17 – 10 equivalents of the guest.

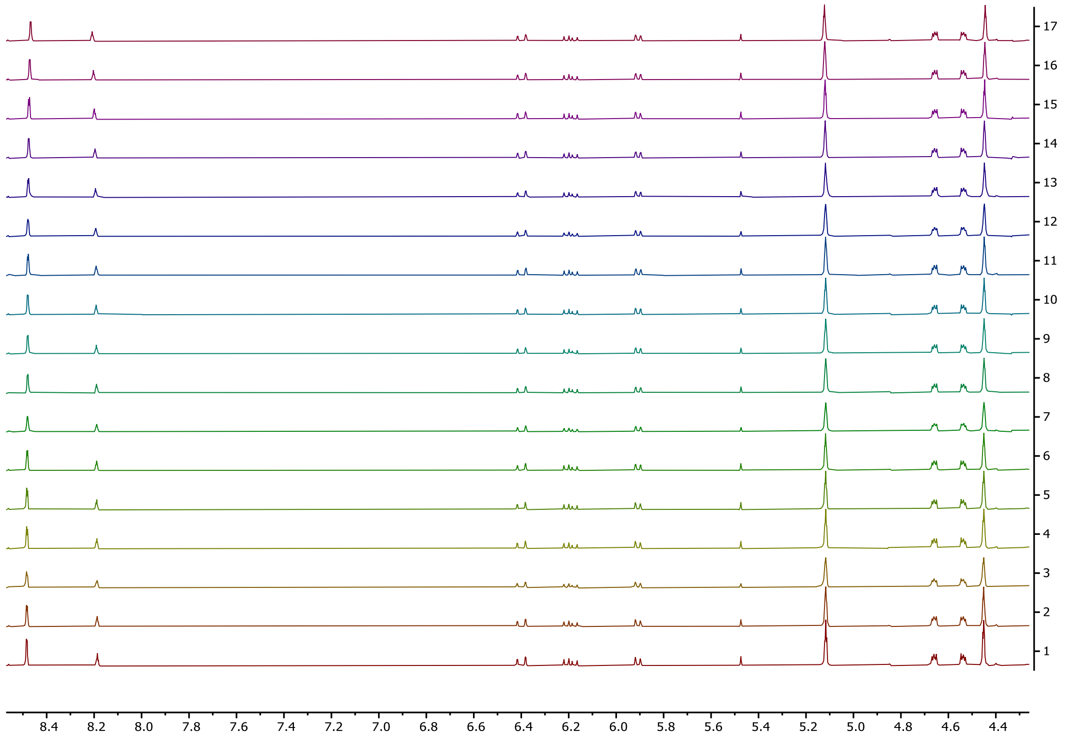

Figure S4.9. Stacked ^{1}H NMR spectra from titration of **1·XB** with TBACl in acetonitrile-d_3:D_2O (97.5:2.5, v/v), 500 MHz, 298 K. Spectrum no. 1 – 0 equivalents of the guest; spectrum no. 17 – 10 equivalents of the guest.

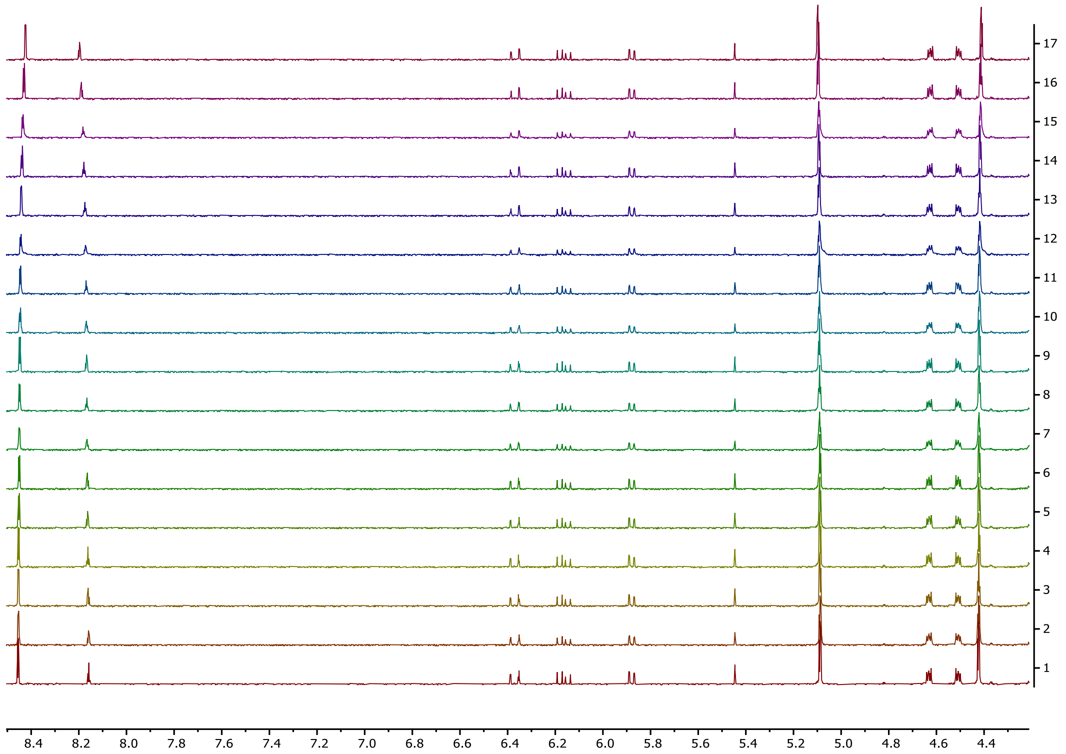

Figure S4.10. Stacked ^{1}H NMR spectra from titration of **1·XB** with TBABr in acetonitrile-d_3:D_2O (97.5:2.5, v/v), 500 MHz, 298 K. Spectrum no. 1 – 0 equivalents of the guest; spectrum no. 17 – 10 equivalents of the guest.

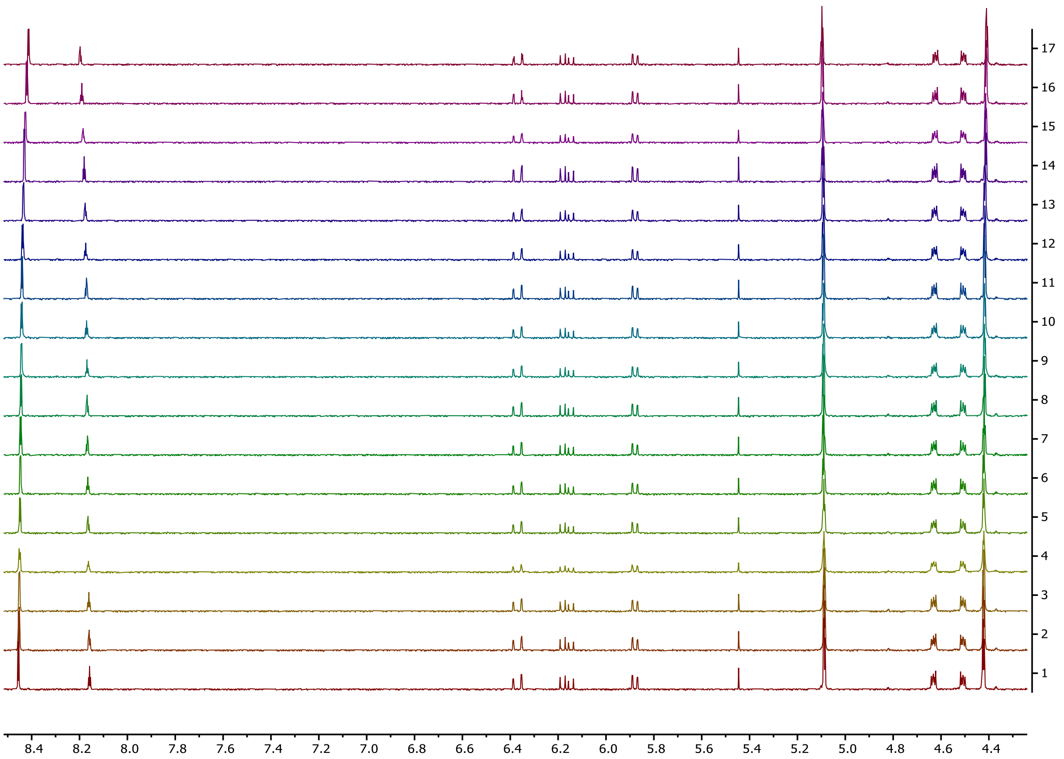

Figure S4.11. Stacked ^{1}H NMR spectra from titration of **1·XB** with TBAI in acetonitrile-d$_3$:D_2O (97.5:2.5, v/v), 500 MHz, 298 K. Spectrum no. 1 – 0 equivalents of the guest; spectrum no. 17 – 10 equivalents of the guest.

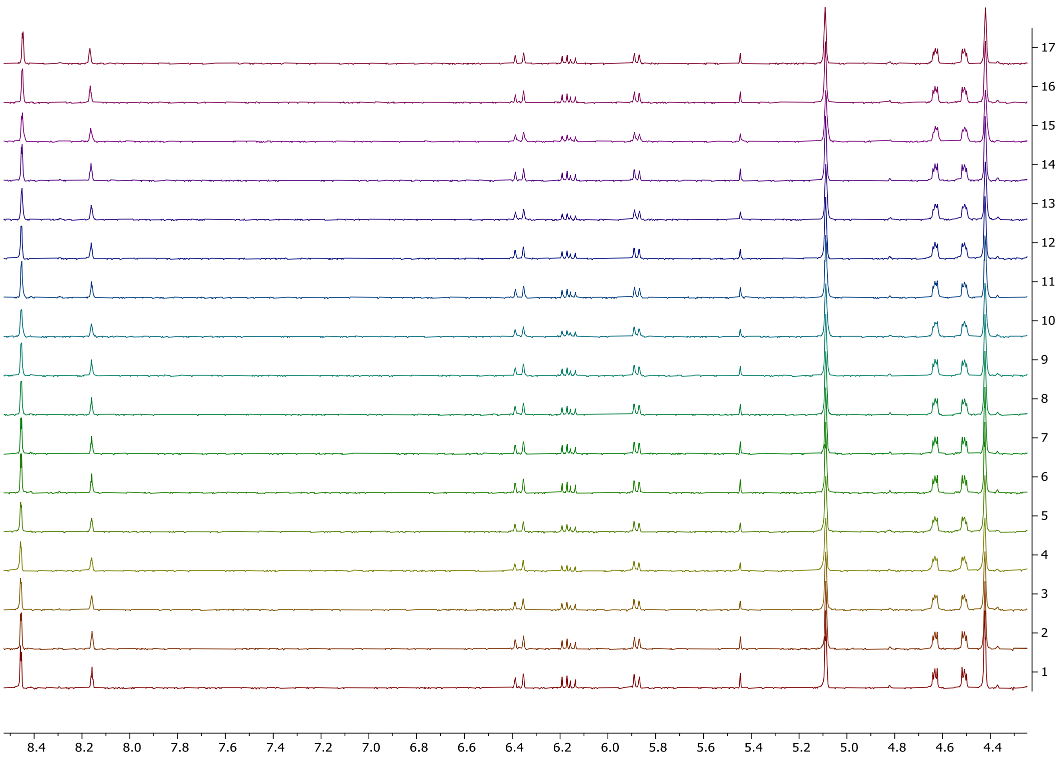

Figure S4.12. Stacked ^{1}H NMR spectra from titration of **1·XB** with $TBAH_2PO_4$ in acetonitrile-d$_3$:D_2O (97.5:2.5, v/v), 500 MHz, 298 K. Spectrum no. 1 – 0 equivalents of the guest; spectrum no. 17 – 10 equivalents of the guest.

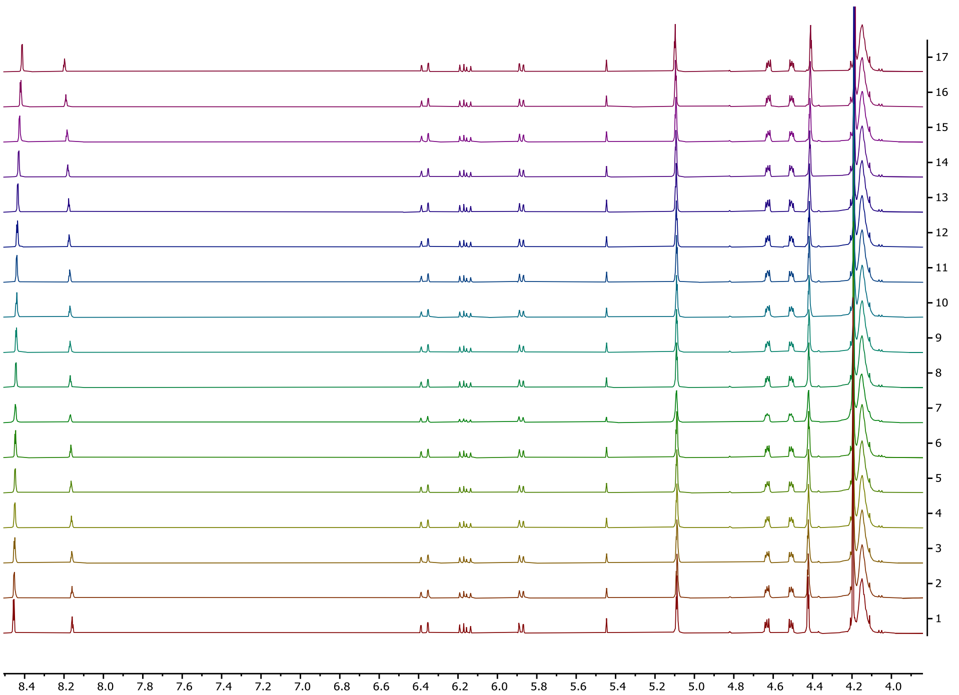

Figure S4.13. Stacked ^{1}H NMR spectra from titration of **1·XB** with TBAI in presence of homopolymer **pDEGA** (concentration of DEGA units 0.009 mM, or 1:9 ratio of **1·XB** to DEGA) in acetonitrile-d_3:D_2O (97.5:2.5, v/v), 500 MHz, 298 K. Spectrum no. 1 – 0 equivalents of the guest; spectrum no. 17 – 10 equivalents of the guest.

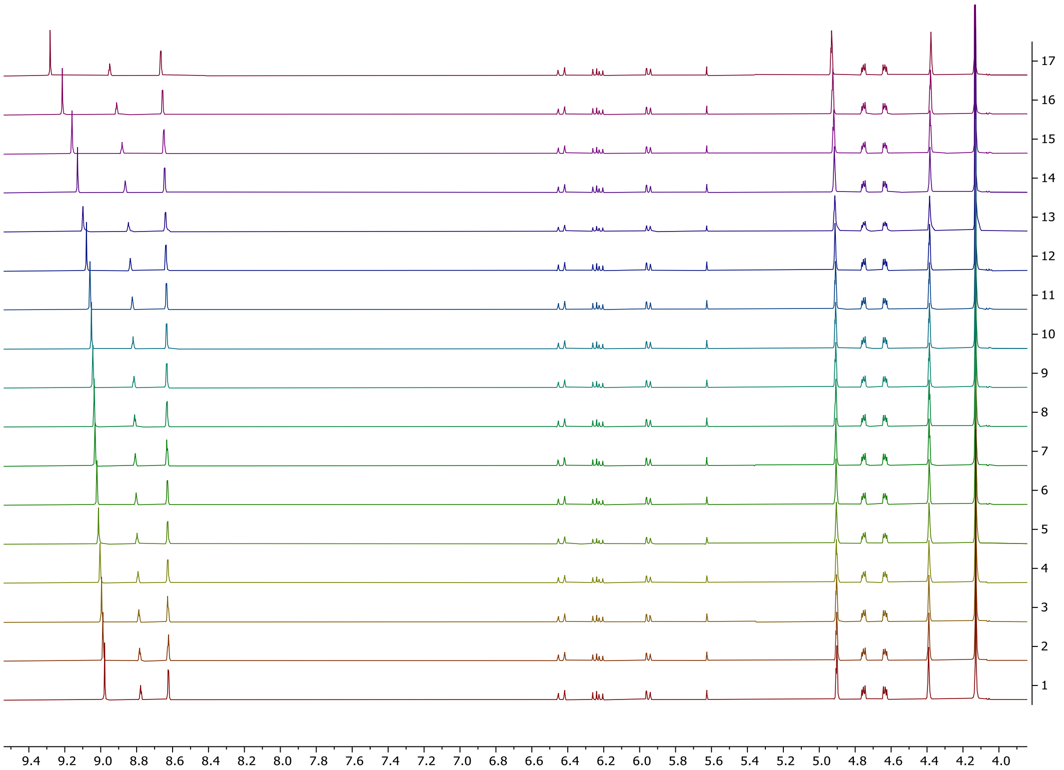

Figure S4.14. Stacked ^{1}H NMR spectra from titration of **1·HB** with TBACl in acetone-d_6:D_2O (97.5:2.5, v/v), 500 MHz, 298 K. Spectrum no. 1 – 0 equivalents of the guest; spectrum no. 17 – 10 equivalents of the guest.

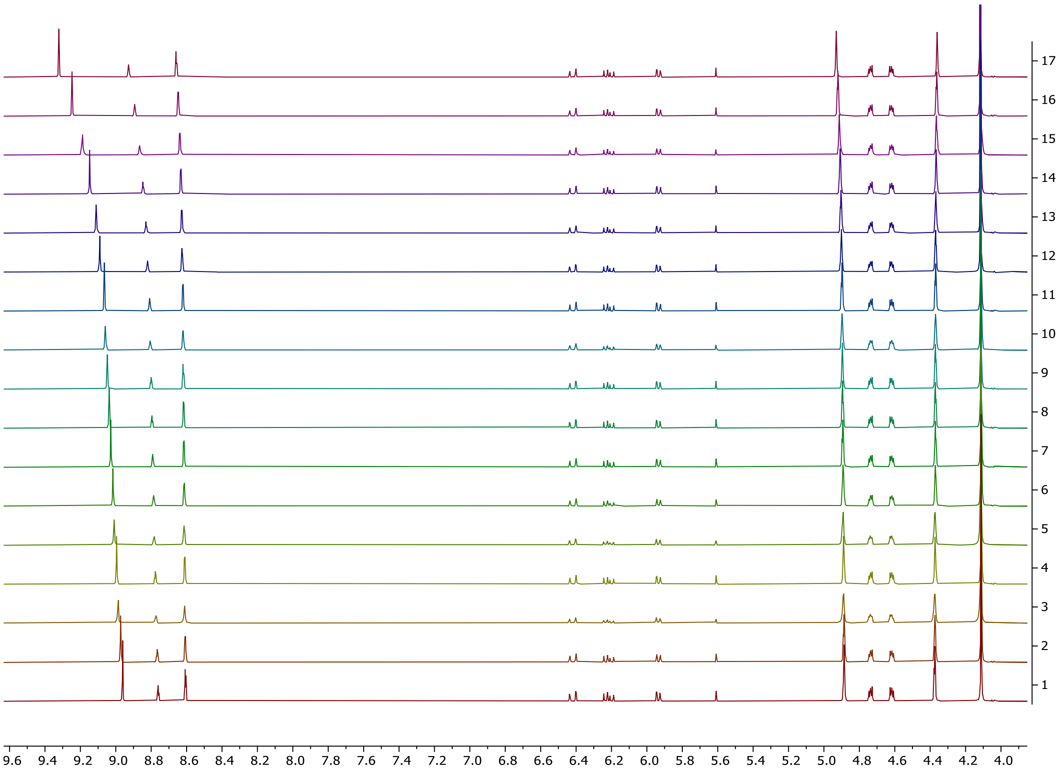

Figure S4.15. Stacked ^{1}H NMR spectra from titration of **1·HB** with TBABr in acetone-d$_6$:D_2O (97.5:2.5, v/v), 500 MHz, 298 K. Spectrum no. 1 – 0 equivalents of the guest; spectrum no. 17 – 10 equivalents of the guest.

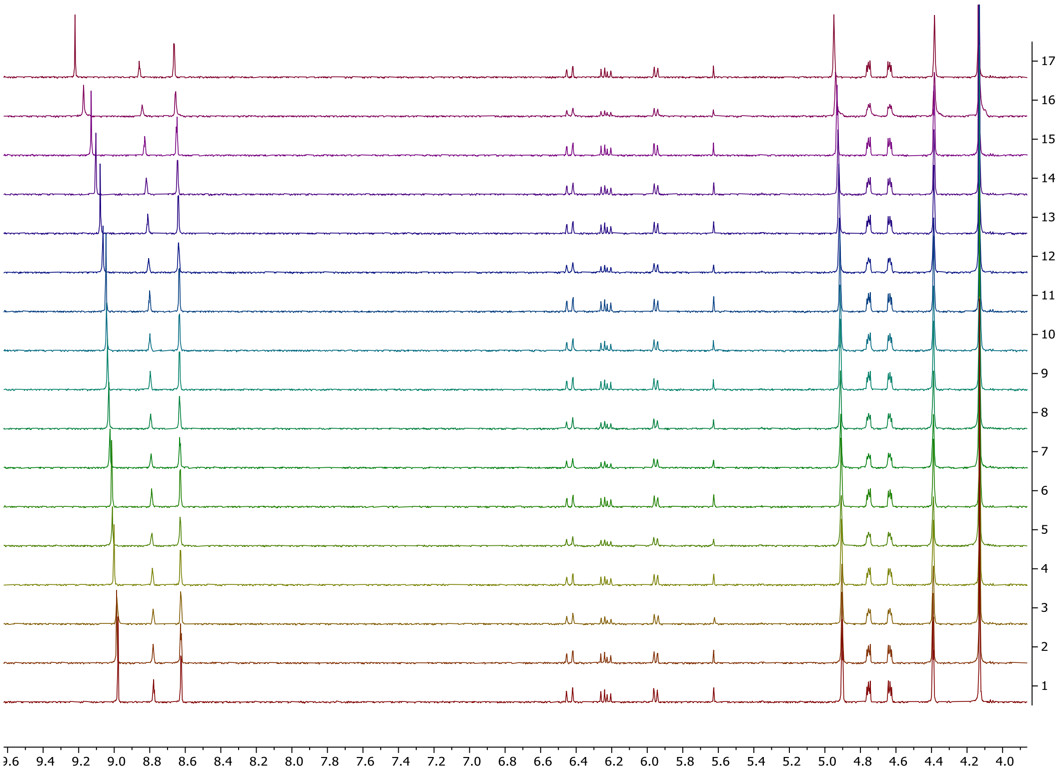

Figure S4.16. Stacked ^{1}H NMR spectra from titration of **1·HB** with TBAI in acetone-d$_6$:D_2O (97.5:2.5, v/v), 500 MHz, 298 K. Spectrum no. 1 – 0 equivalents of the guest; spectrum no. 17 – 10 equivalents of the guest.

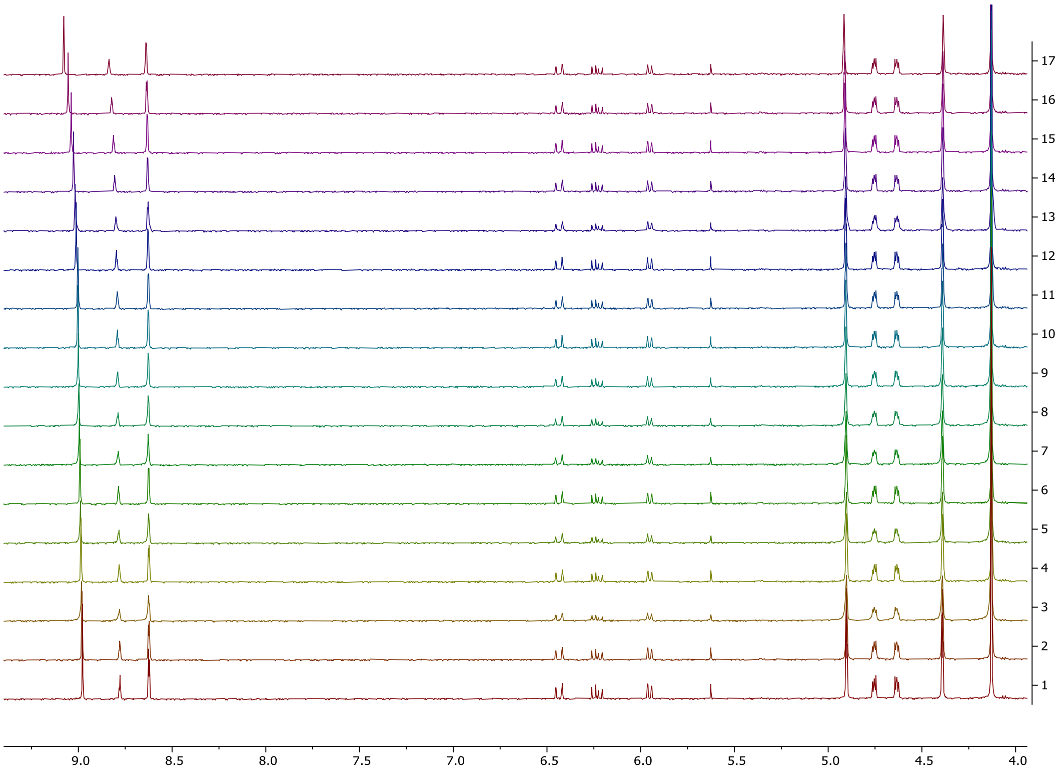

Figure S4.17. Stacked ^{1}H NMR spectra from titration of **1·HB** with $TBAH_2PO_4$ in acetone-d_6:D_2O (97.5:2.5, v/v), 500 MHz, 298 K. Spectrum no. 1 – 0 equivalents of the guest; spectrum no. 17 – 10 equivalents of the guest.

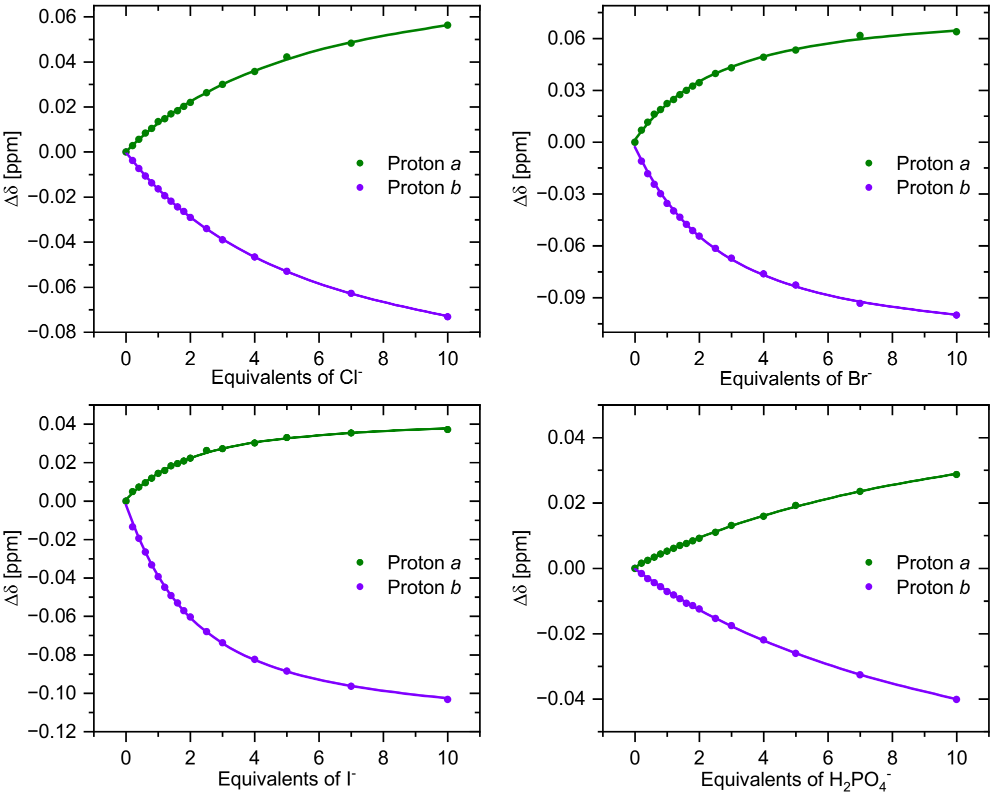

Figure S4.18. Anion binding isotherms for **1·XB** in acetone-d_6:D_2O (97.5:2.5, v/v), where circles represent experimental data and the lines represent the fitted isotherm.

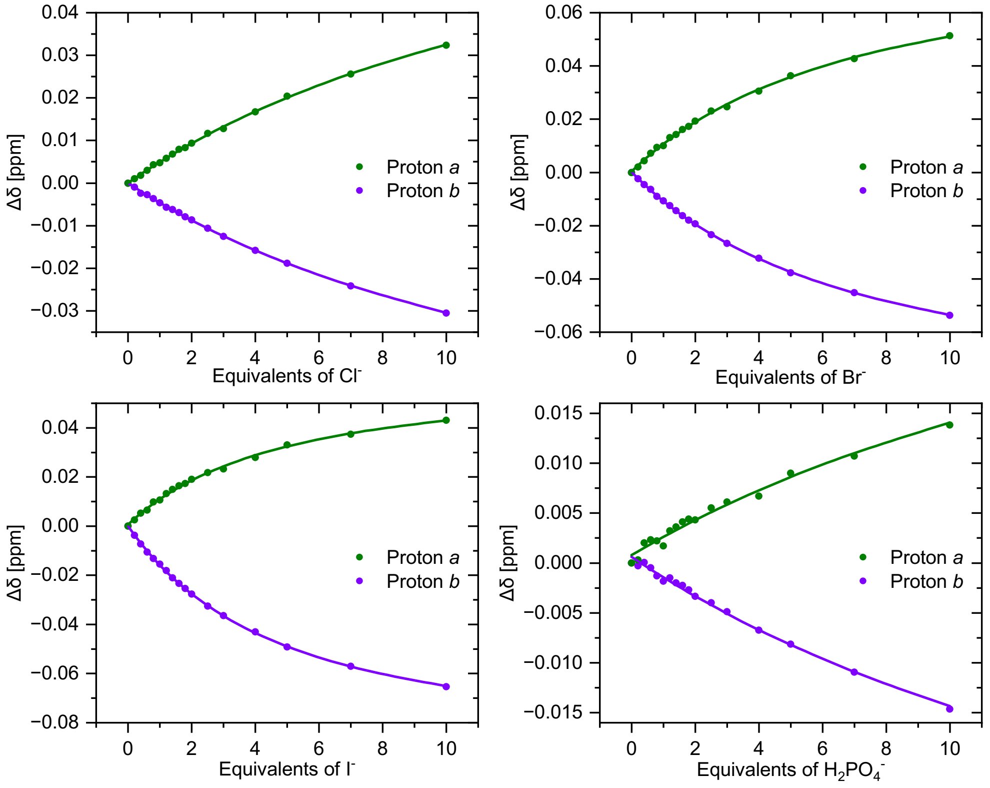

Figure S4.19. Anion binding isotherms for **1·XB** in acetone-d$_6$:acetonitrile-d$_3$:D_2O (48.75:48.75:2.5, v/v/v), where circles represent experimental data and the lines represent the fitted isotherm.

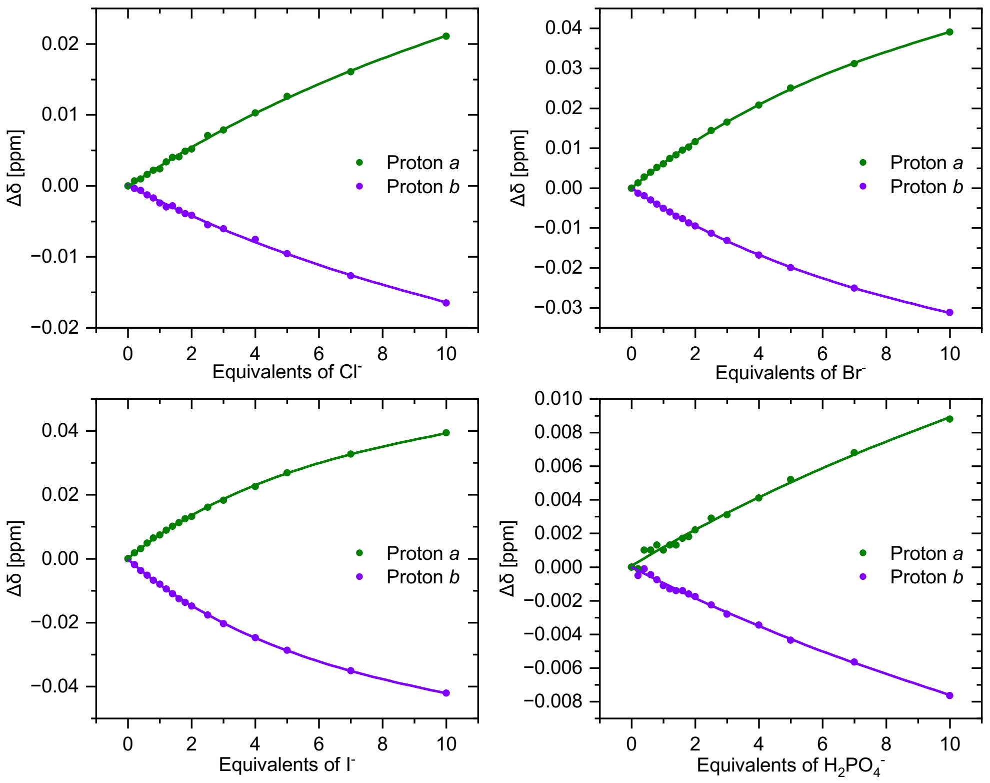

Figure S4.20. Anion binding isotherms for **1·XB** in acetonitrile-d$_3$:D_2O (97.5:2.5, v/v), where circles represent experimental data and the lines represent the fitted isotherm.

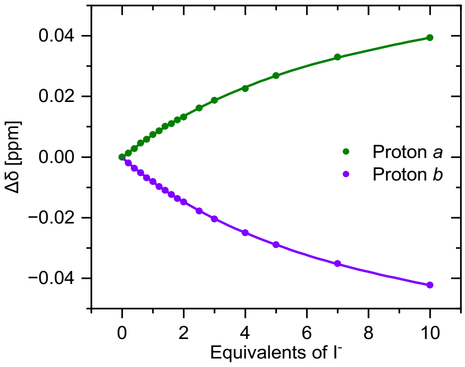

Figure S4.21. Anion binding isotherms for **1·XB** in presence of homopolymer **pDEGA** (concentration of DEGA units 0.009 mM, or 1:9 ratio of **1·XB** to DEGA) in acetonitrile-d_3:D_2O (97.5:2.5, v/v), where circles represent experimental data and the lines represent the fitted isotherm.

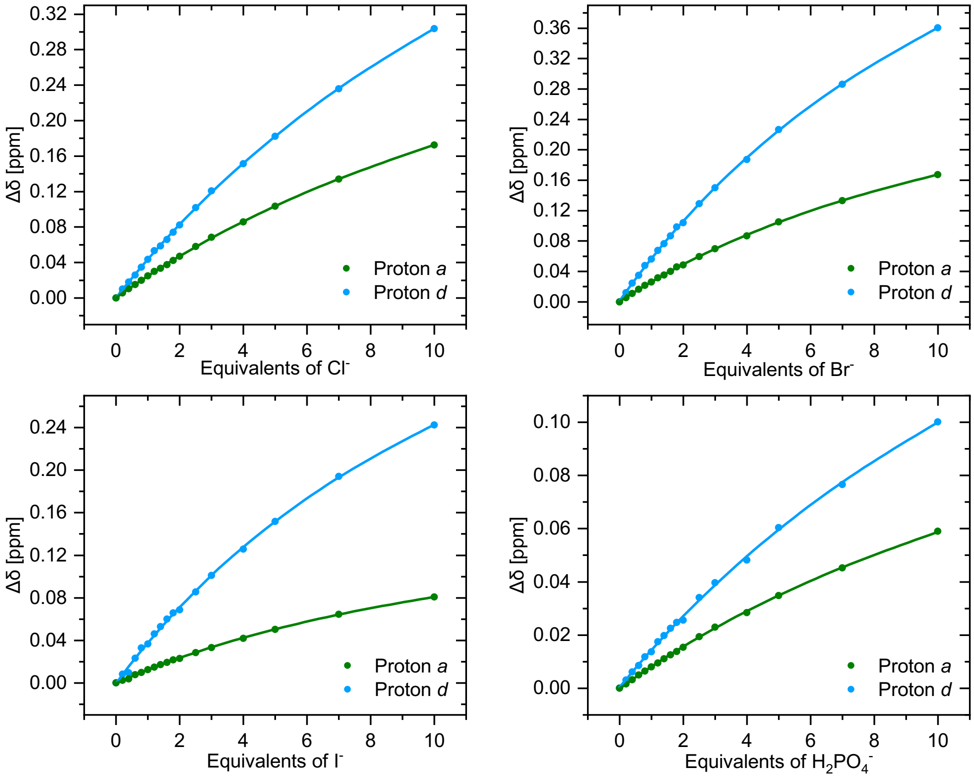

Figure S4.22. Anion binding isotherms for **1·HB** in acetone-d_6:D_2O (97.5:2.5, v/v), where circles represent experimental data and the lines represent the fitted isotherm.

Table S4.1. Anion association constants of monomeric receptors **1·XB** and **1·HB** determined by ^{1}H NMR titrations in 97.5:2.5 acetone-d_6:D_2O, 48.75:48.75:2.5acetone-d_6/ACN-d_3/D_2O and 97.5:2.5 ACN-d_3/D_2O. Errors estimated to be 10%. ND – not determined.

	K (M^{-1})					
	97.5:2.5 acetone-d_6/D_2O		48.75:48.75:2.5 acetone-d_6/ACN-d_3/D_2O		97.5:2.5 ACN-d_3/H_2O	
	**1·HB**	**1·XB**	**1·HB**	**1·XB**	**1·HB**	**1·XB**
Cl^-	30	170	ND	40	ND	20
Br^-	50	470	ND	130	ND	60
I^-	50	640	ND	220	ND	110
$H_2PO_4^-$	30	60	ND	25	ND	< 10

Table S4.2. Ratio of anion association constants of monomeric receptors **1·XB** and **1·HB**, representing the halogen bonding enhancement factor (BEF_{XB} = $K_{\mathbf{1\cdot XB}}/K_{\mathbf{1\cdot HB}}$) determined by ^{1}H NMR titrations in 97.5:2.5 acetone-d_6/D_2O. Errors estimated to be 15%.

	$K_{\mathbf{1\cdot XB}}/K_{\mathbf{1\cdot HB}}$
Cl^-	5.7
Br^-	9.4
I^-	12.8
$H_2PO_4^-$	2.0

S5 Anion Binding Studies of Polymeric Hosts by ^{1}H NMR

A 50 mM solution of the chosen TBA salt of an anion (TBAX, X = I^-, Br^-, Cl^- or $H_2PO_4^-$) was added to 0.5 mL solution containing 1 mM of binding unit **1·XB**/**HB** within the polymeric hosts in the chosen solvent system:

a) acetone-d_6:D_2O 97.5:2.5, v/v;
b) acetone-d_6:acetonitrile-d_3:D_2O 48.75:48.75:2.5, v/v/v;
c) or acetonitrile-d_3:D_2O 97.5:2.5, v/v.

Each titration isotherm comprises 17 data points, corresponding to 0.0, 0.2, 0.4, 0.6, 0.8, 1.0, 1.2, 1.4, 1.6, 1.8, 2, 2.5, 3.0, 4.0, 5.0, 7.0, 10.0 equivalents of added guest anion (100 µl of guest solution added by the last point). Addition of the solution containing the guest caused dilution of the host, which was accounted for in calculations. Binding constants were determined using BindFit, applying a 1:1 host-guest binding model in all cases. At least two signals were simultaneously used to determine binding constants (global fitting approach – binding constant used a shared parameter). Fitting using 2:1 host-guest model failed in all cases. Only signals with high perturbation were used for determining binding constants. Concentrations of polymers are presented in Table S5.1.

Table S5.1. Concentration of polymers used during titrations with TBAX (X = I^-, Br^-, Cl^- or $H_2PO_4^-$) and list of signals used for fitting

	Concentration of polymer (mM)	Signals used for fitting
**pDEGA-1·XB**	0.174	*b, c*
**pDEGA-0.5·XB**	0.246	*b, c*
**pDEGA-BuA-1·XB**	0.143	*b, c*
**pDEGA-1·HB**	0.0871	*a, d*

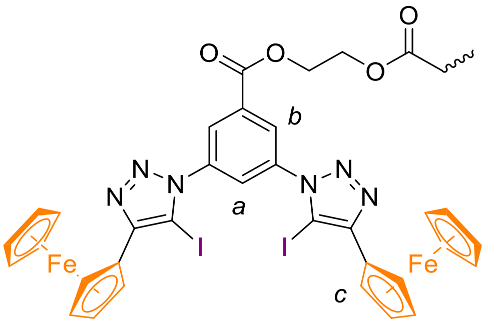

1·XB

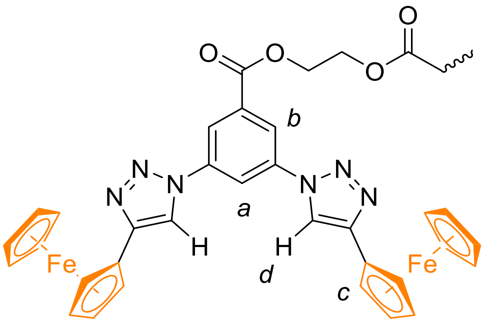

1·HB

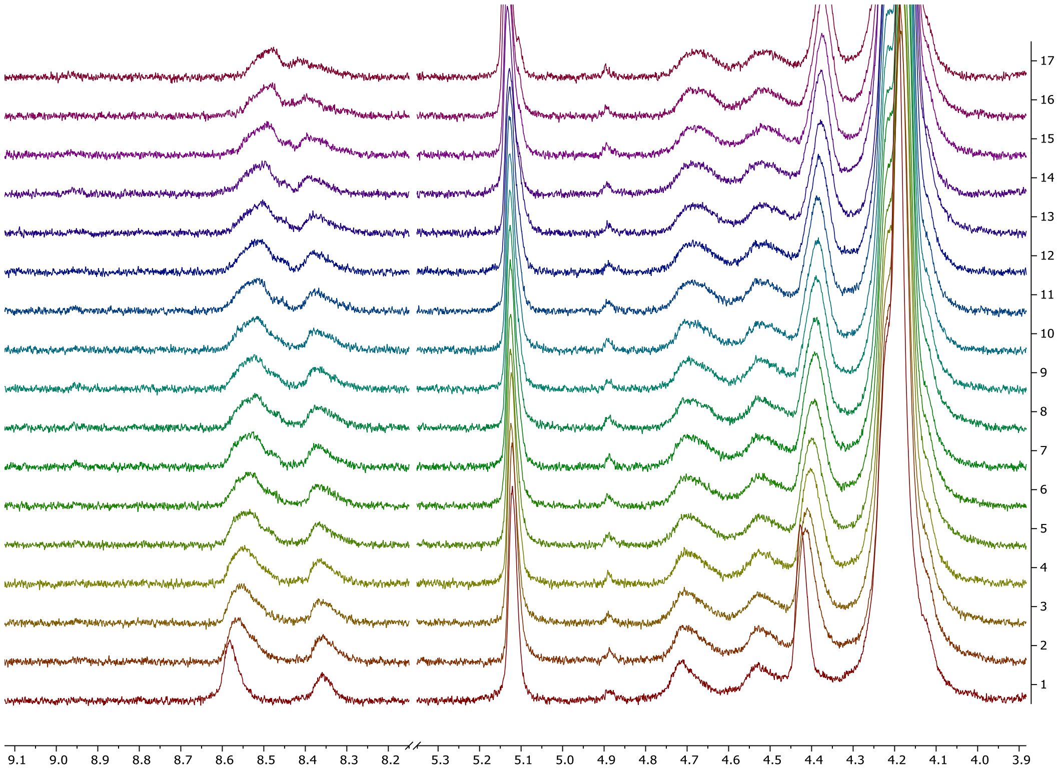

Figure S5.1. Stacked ^{1}H NMR spectra from titration of **pDEGA-1·XB** with TBACl in acetone-d_6:D_2O (97.5:2.5, v/v), 500 MHz, 298 K. Spectrum no. 1 – 0 equivalents of the guest; spectrum no. 17 – 10 equivalents of the guest.

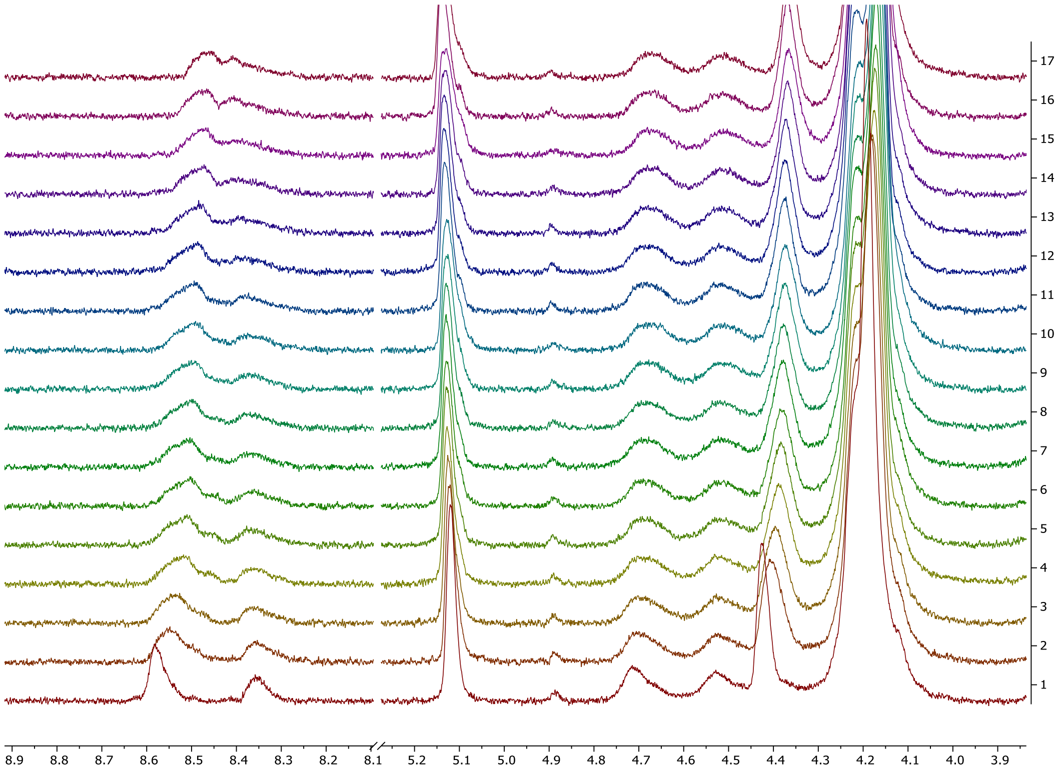

Figure S5.2. Stacked ^{1}H NMR spectra from titration of **pDEGA-1·XB** with TBABr in acetone-d_6:D_2O (97.5:2.5, v/v), 500 MHz, 298 K. Spectrum no. 1 – 0 equivalents of the guest; spectrum no. 17 – 10 equivalents of the guest.

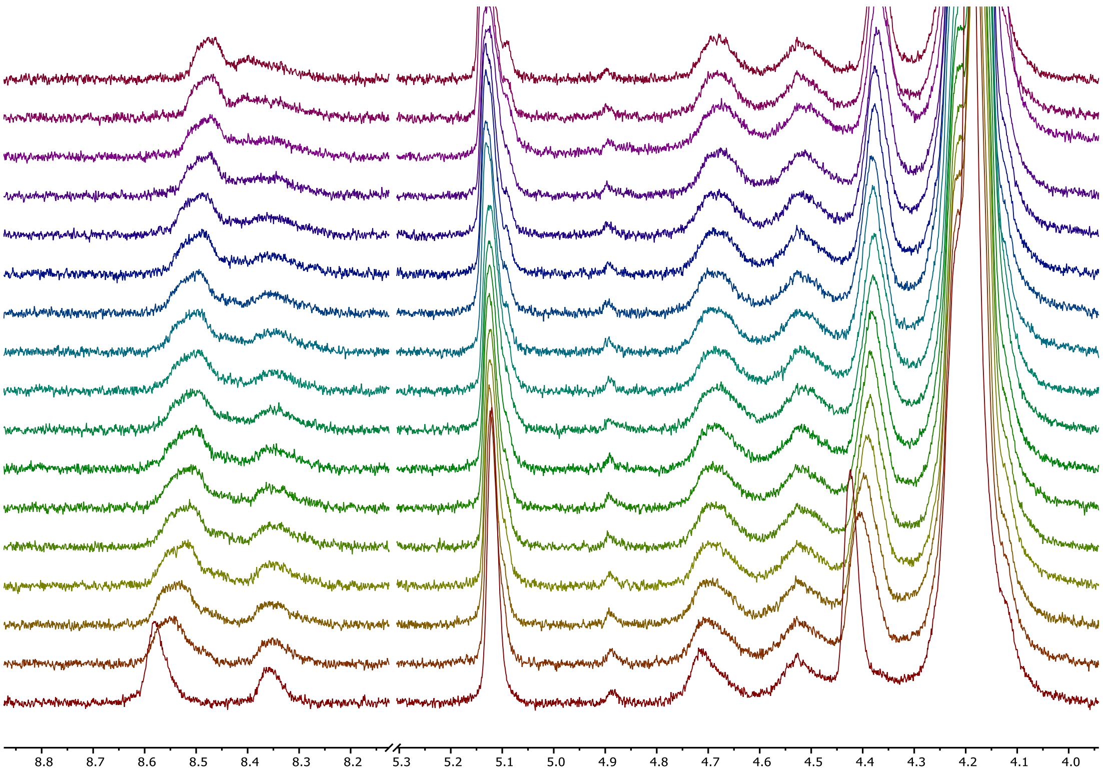

Figure S5.3. Stacked ^{1}H NMR spectra from titration of **pDEGA-1·XB** with TBAI in acetone-d_6:D_2O (97.5:2.5, v/v), 500 MHz, 298 K. Spectrum no. 1 – 0 equivalents of the guest; spectrum no. 17 – 10 equivalents of the guest.

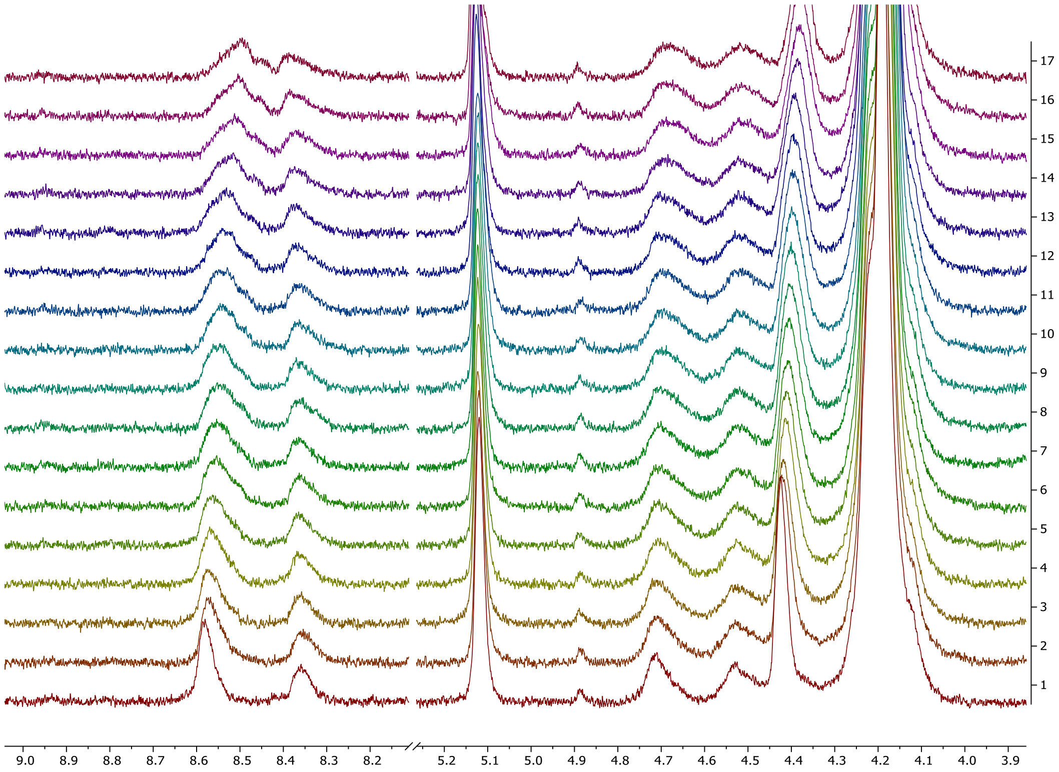

Figure S5.4. Stacked ^{1}H NMR spectra from titration of **pDEGA-1·XB** with $TBAH_2PO_4$ in acetone-d_6:D_2O (97.5:2.5, v/v), 500 MHz, 298 K. Spectrum no. 1 – 0 equivalents of the guest; spectrum no. 17 – 10 equivalents of the guest.

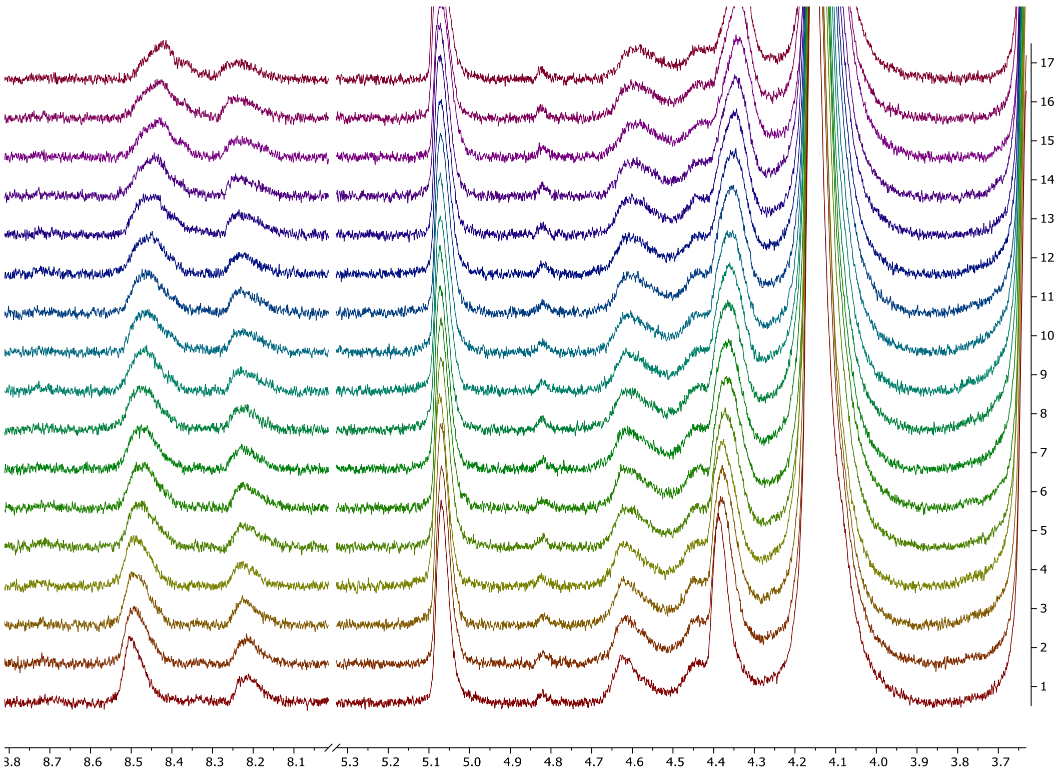

Figure S5.5. Stacked ^{1}H NMR spectra from titration of **pDEGA-1·XB** with TBACl in acetone-d_6:acetonitrile-d_3:D_2O (48.75:48.75:2.5, v/v/v), 500 MHz, 298 K. Spectrum no. 1 – 0 equivalents of the guest; spectrum no. 17 – 10 equivalents of the guest.

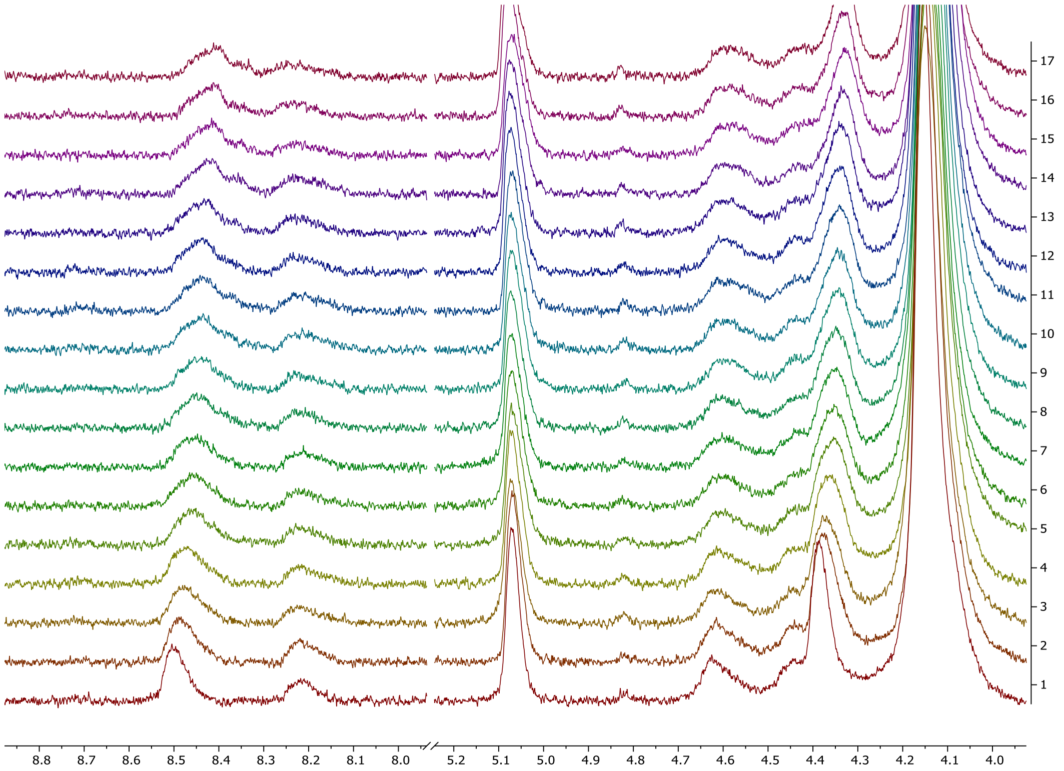

Figure S5.6. Stacked ^{1}H NMR spectra from titration of **pDEGA-1·XB** with TBABr in acetone-d_6:acetonitrile-d_3:D_2O (48.75:48.75:2.5, v/v/v), 500 MHz, 298 K. Spectrum no. 1 – 0 equivalents of the guest; spectrum no. 17 – 10 equivalents of the guest.

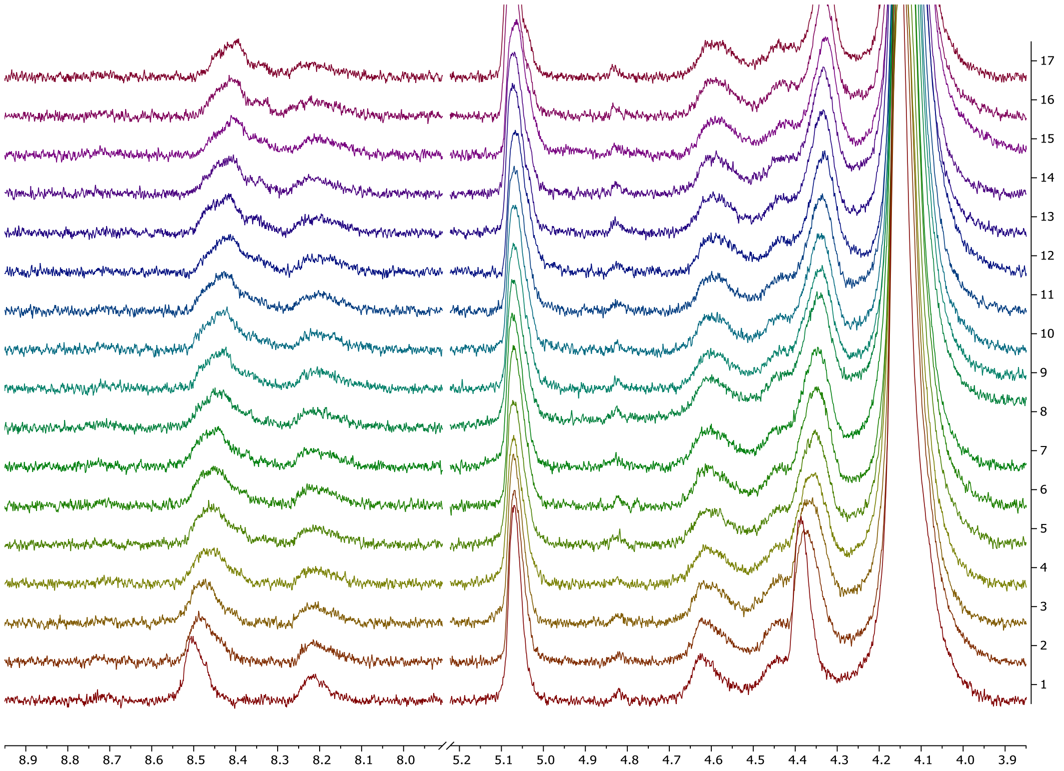

Figure S5.7. Stacked ^{1}H NMR spectra from titration of **pDEGA-1·XB** with TBAI in acetone-d$_6$:acetonitrile-d$_3$:D_2O (48.75:48.75:2.5, v/v/v), 500 MHz, 298 K. Spectrum no. 1 – 0 equivalents of the guest; spectrum no. 17 – 10 equivalents of the guest.

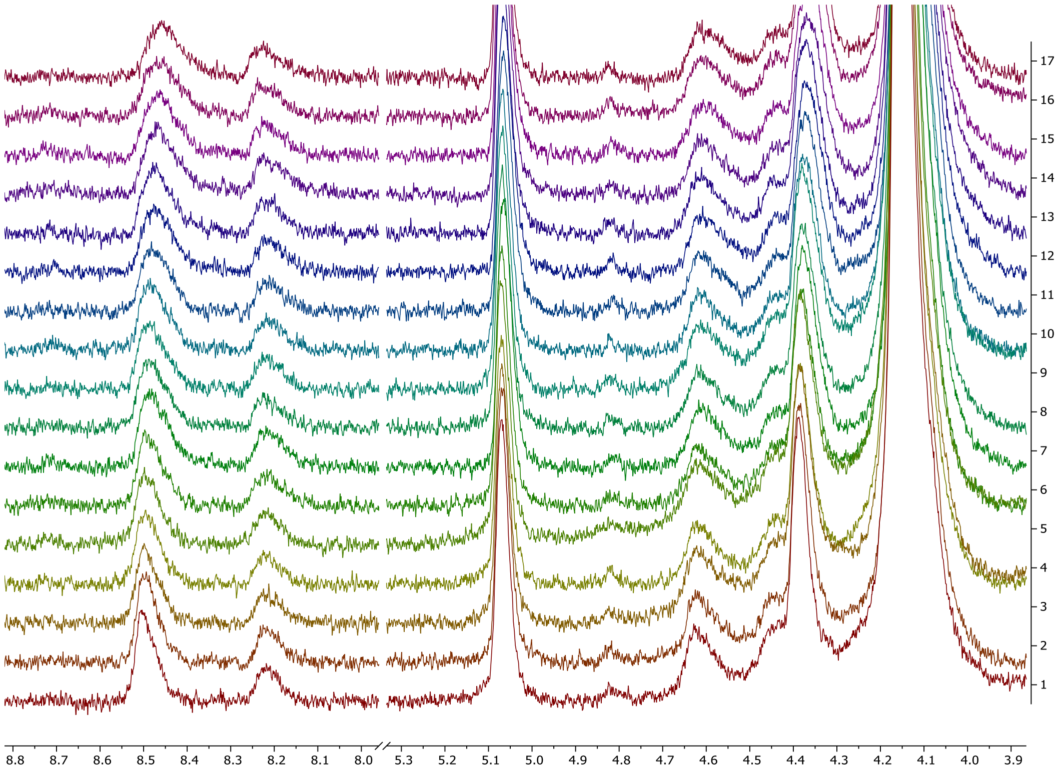

Figure S5.8. Stacked ^{1}H NMR spectra from titration of **pDEGA-1·XB** with $TBAH_2PO_4$ in acetone-d$_6$:acetonitrile-d$_3$:D_2O (48.75:48.75:2.5, v/v/v), 500 MHz, 298 K. Spectrum no. 1 – 0 equivalents of the guest; spectrum no. 17 – 10 equivalents of the guest.

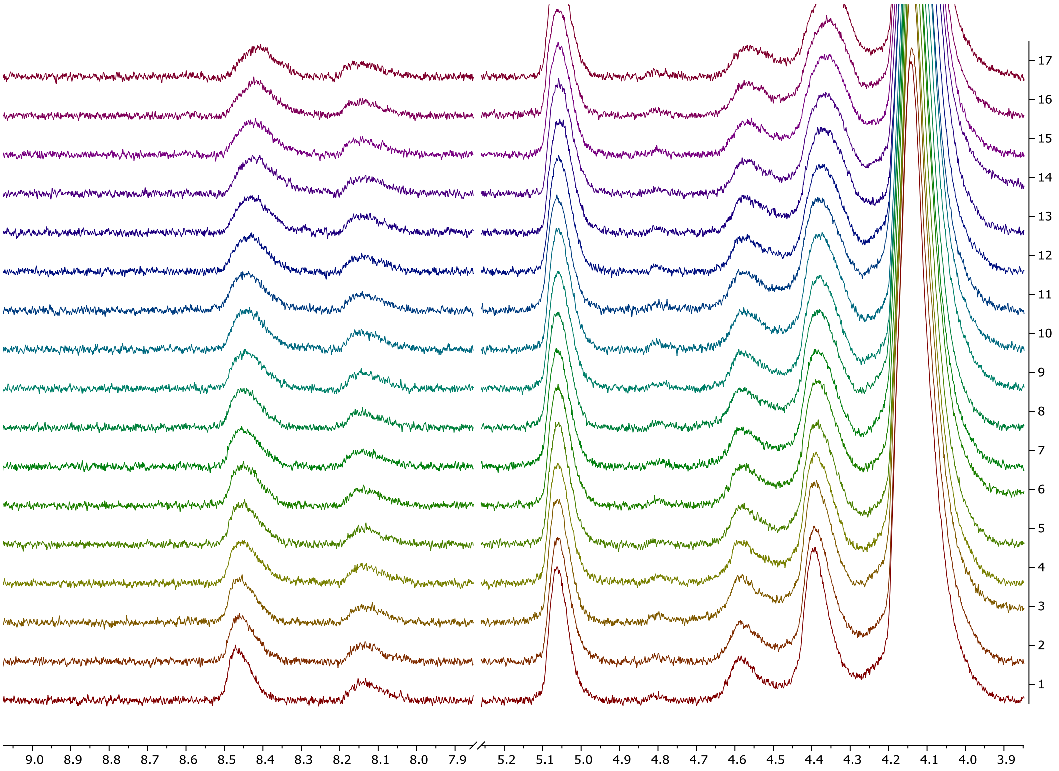

Figure S5.9. Stacked ^{1}H NMR spectra from titration of **pDEGA-1·XB** with TBACl in acetonitrile-d_3:D_2O (97.5:2.5, v/v), 500 MHz, 298 K. Spectrum no. 1 – 0 equivalents of the guest; spectrum no. 17 – 10 equivalents of the guest.

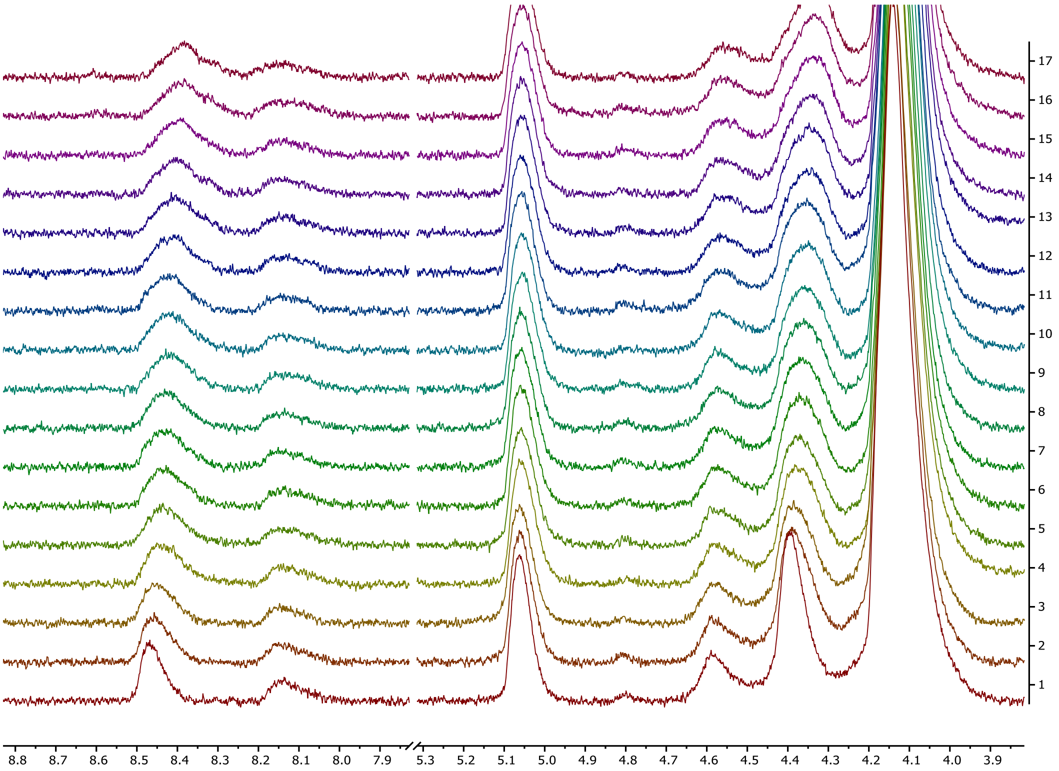

Figure S5.10. Stacked ^{1}H NMR spectra from titration of **pDEGA-1·XB** with TBABr in acetonitrile-d_3:D_2O (97.5:2.5, v/v), 500 MHz, 298 K. Spectrum no. 1 – 0 equivalents of the guest; spectrum no. 17 – 10 equivalents of the guest.

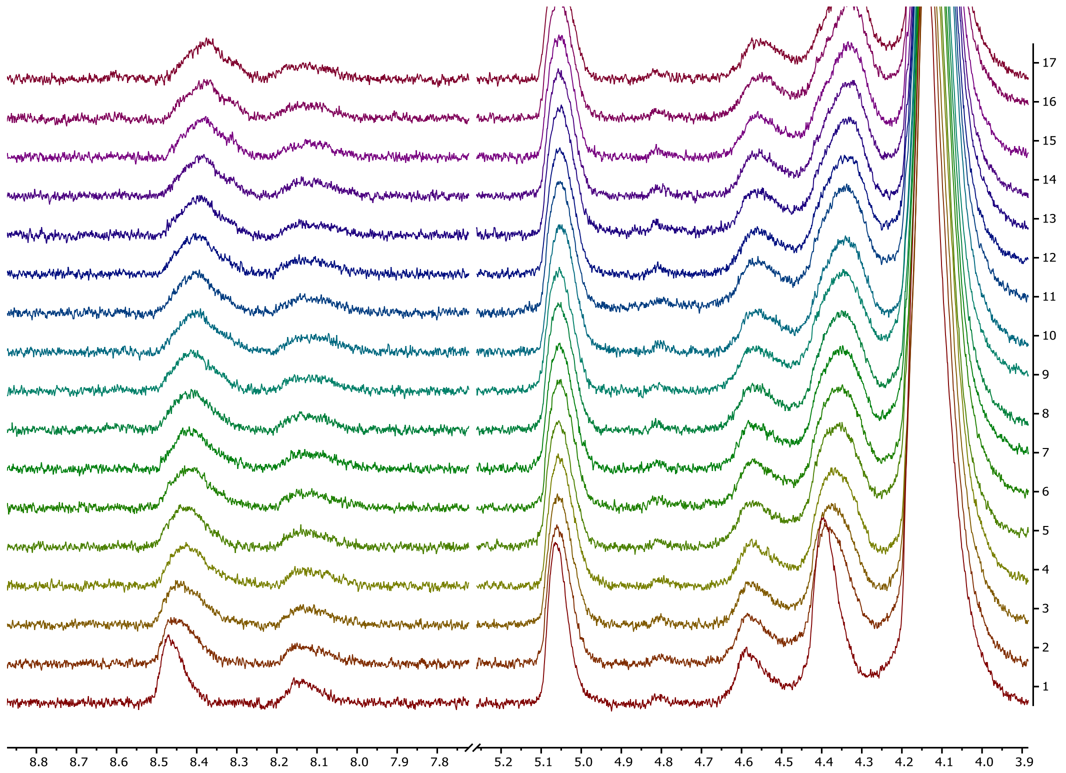

Figure S5.11. Stacked ^1H NMR spectra from titration of **pDEGA-1·XB** with TBAI in acetonitrile-d_3:D_2O (97.5:2.5, v/v), 500 MHz, 298 K. Spectrum no. 1 – 0 equivalents of the guest; spectrum no. 17 – 10 equivalents of the guest.

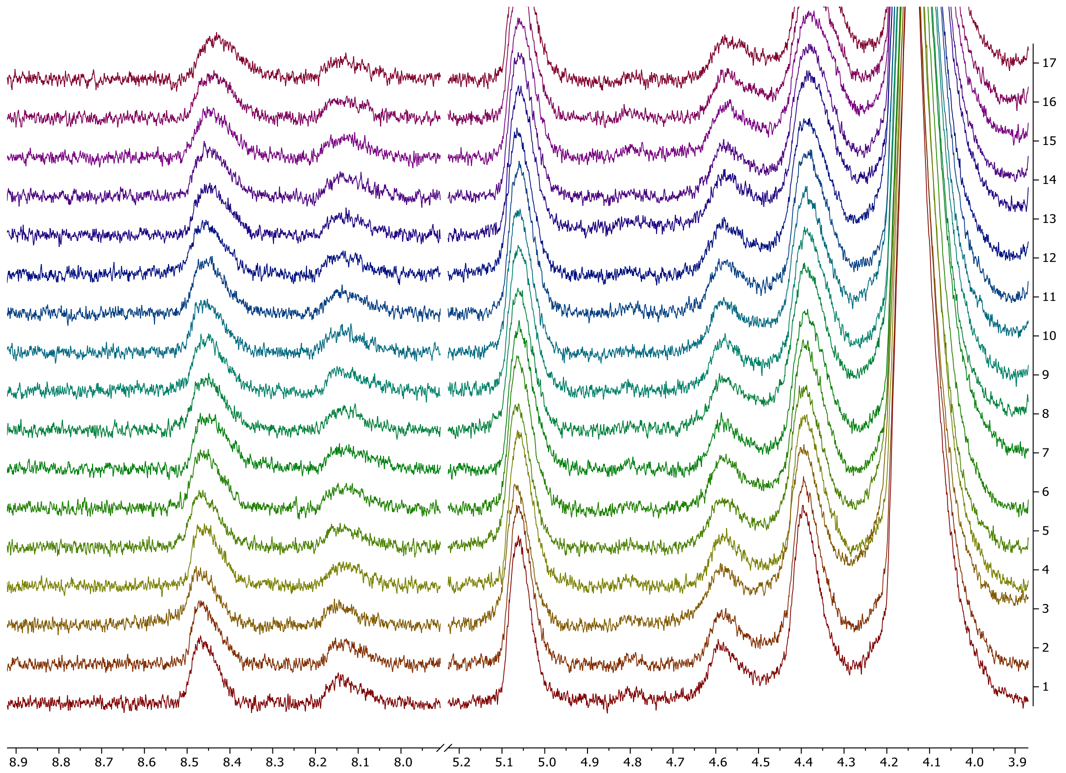

Figure S5.12. Stacked ^1H NMR spectra from titration of **pDEGA-1·XB** with $TBAH_2PO_4$ in acetonitrile-d_3:D_2O (97.5:2.5, v/v), 500 MHz, 298 K. Spectrum no. 1 – 0 equivalents of the guest; spectrum no. 17 – 10 equivalents of the guest.

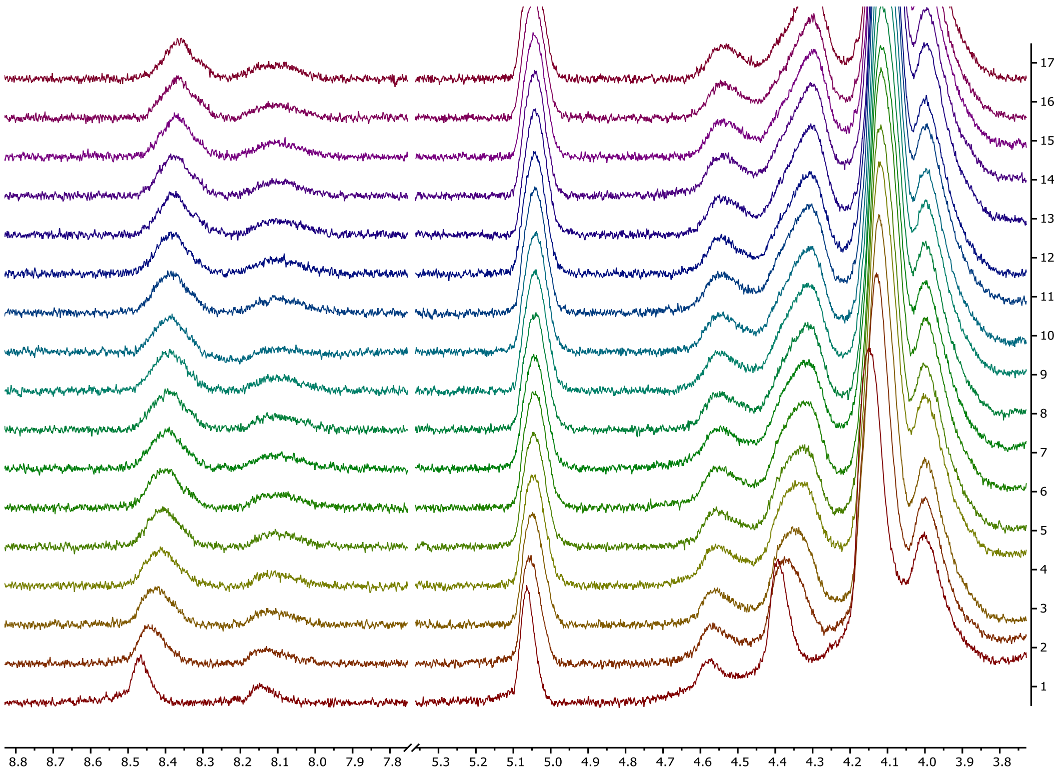

Figure S5.13. Stacked ^{1}H NMR spectra from titration of **pDEGA-BuA-1·XB** with TBAI in acetonitrile-d$_3$:D_2O (97.5:2.5, v/v), 500 MHz, 298 K. Spectrum no. 1 – 0 equivalents of the guest; spectrum no. 17 – 10 equivalents of the guest.

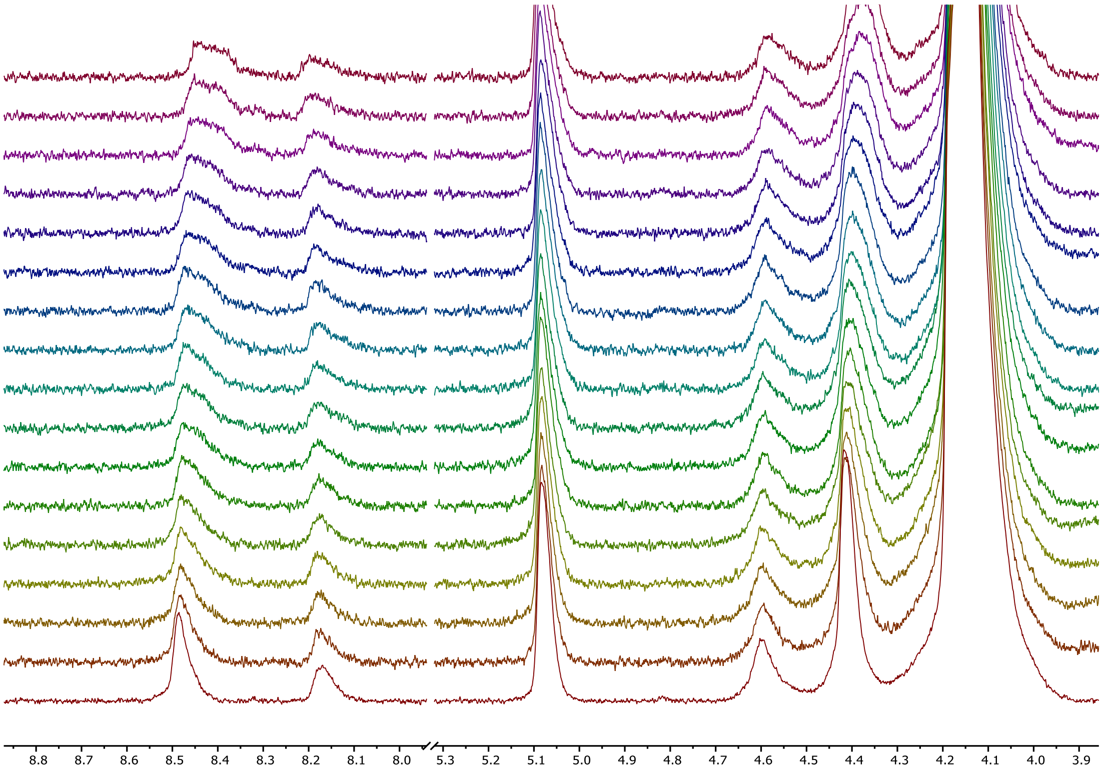

Figure S5.14. Stacked ^{1}H NMR spectra from titration of **pDEGA-1·XB$_{0.5}$** with TBAI in acetonitrile-d$_3$:D_2O (97.5:2.5, v/v), 500 MHz, 298 K. Spectrum no. 1 – 0 equivalents of the guest; spectrum no. 17 – 10 equivalents of the guest.

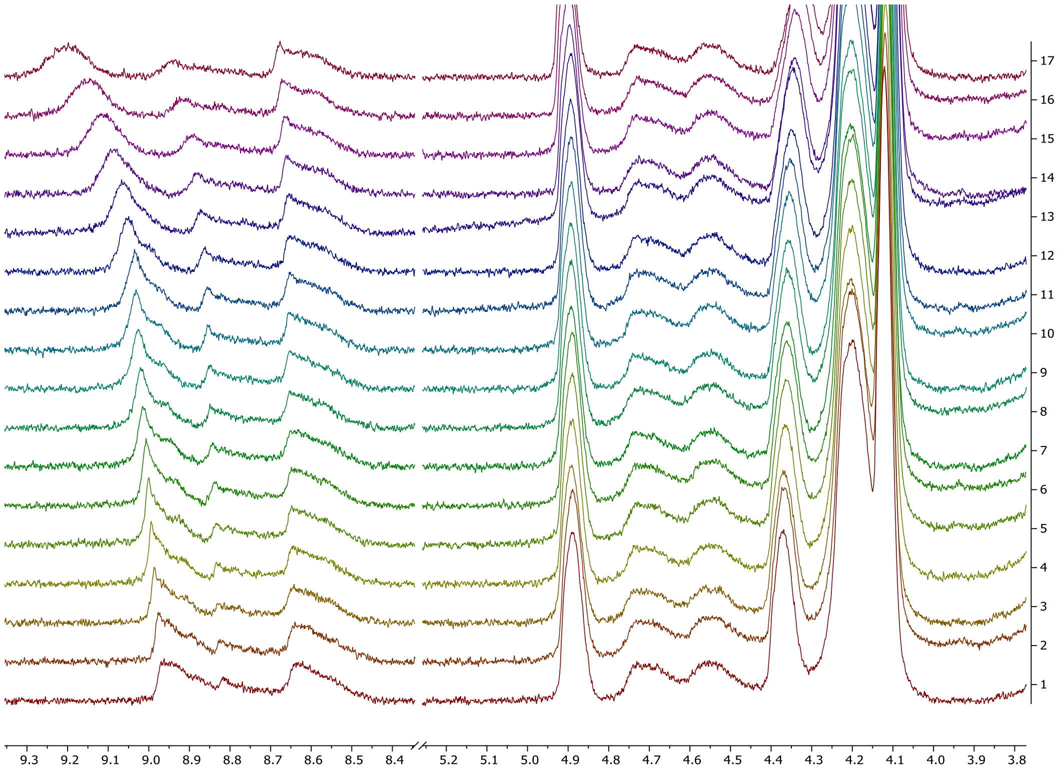

Figure S5.15. Stacked ^{1}H NMR spectra from titration of **pDEGA-1·HB** with TBACl in acetone-d_6:D_2O (97.5:2.5, v/v), 500 MHz, 298 K. Spectrum no. 1 – 0 equivalents of the guest; spectrum no. 17 – 10 equivalents of the guest.

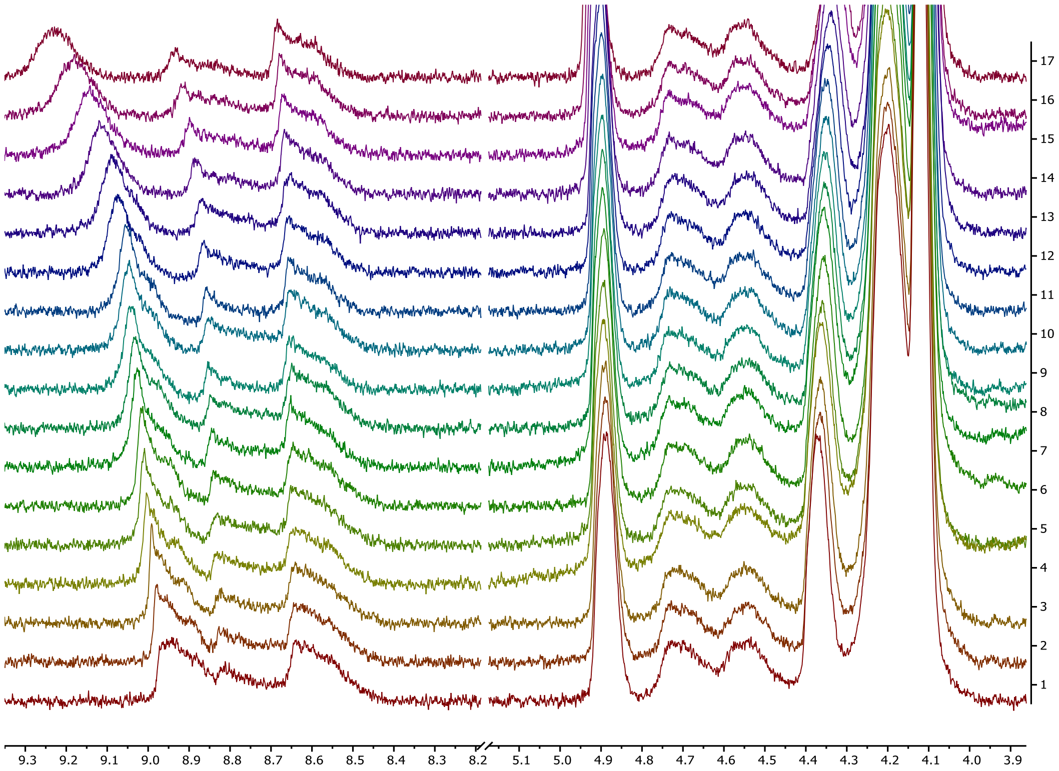

Figure S5.16. Stacked ^{1}H NMR spectra from titration of **pDEGA-1·HB** with TBABr in acetone-d_6:D_2O (97.5:2.5, v/v), 500 MHz, 298 K. Spectrum no. 1 – 0 equivalents of the guest; spectrum no. 17 – 10 equivalents of the guest.

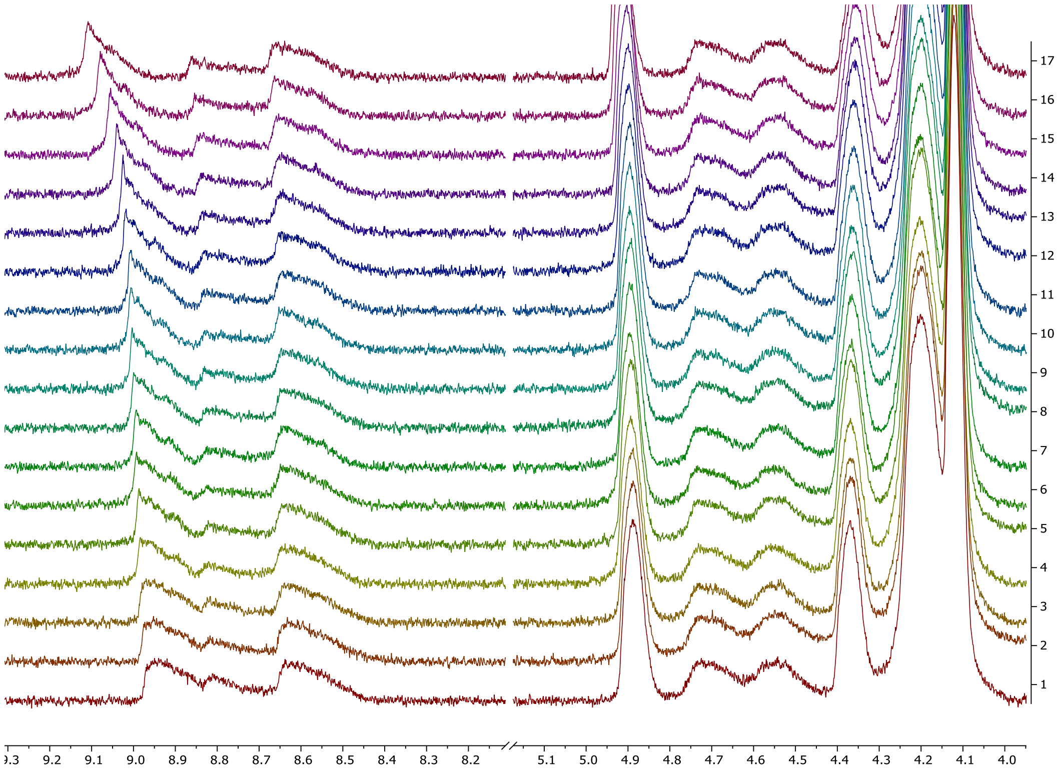

Figure S5.17. Stacked ^{1}H NMR spectra from titration of **pDEGA-1·XB** with TBAI in acetone-d_6:D_2O (97.5:2.5, v/v), 500 MHz, 298 K. Spectrum no. 1 – 0 equivalents of the guest; spectrum no. 17 – 10 equivalents of the guest.

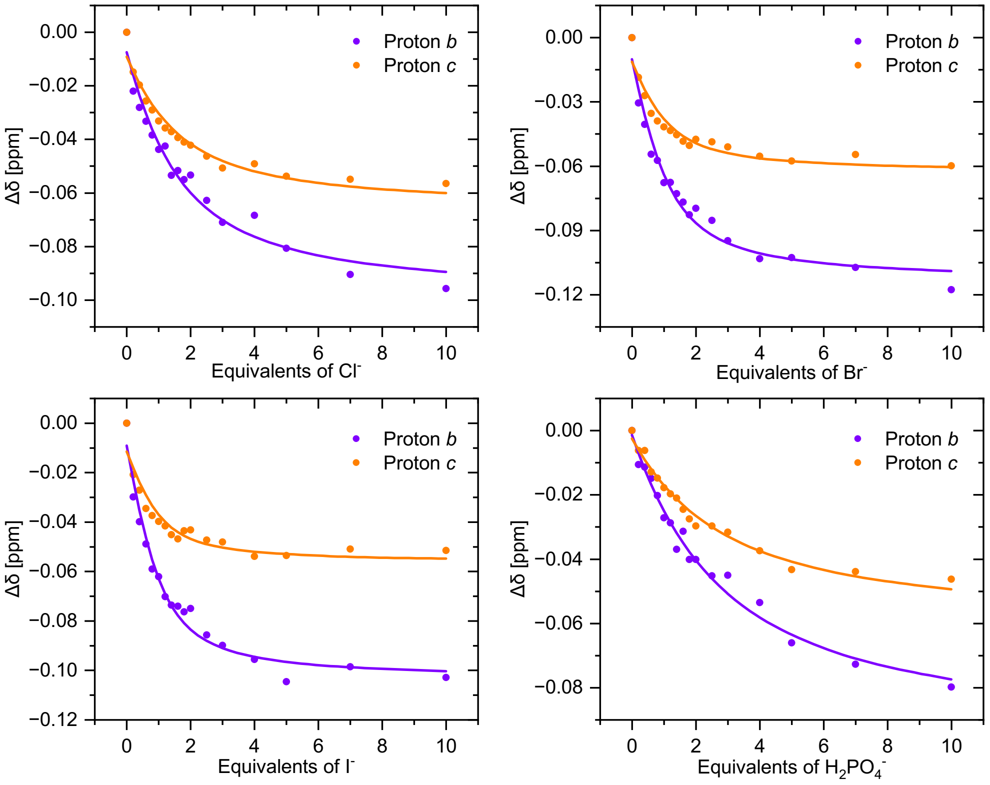

Figure S5.18. Examples of anion binding isotherms for **pDEGA-1·XB** in acetone-d_6:D_2O (97.5:2.5, v/v), where circles represent experimental data and the lines represent the fitted isotherm.

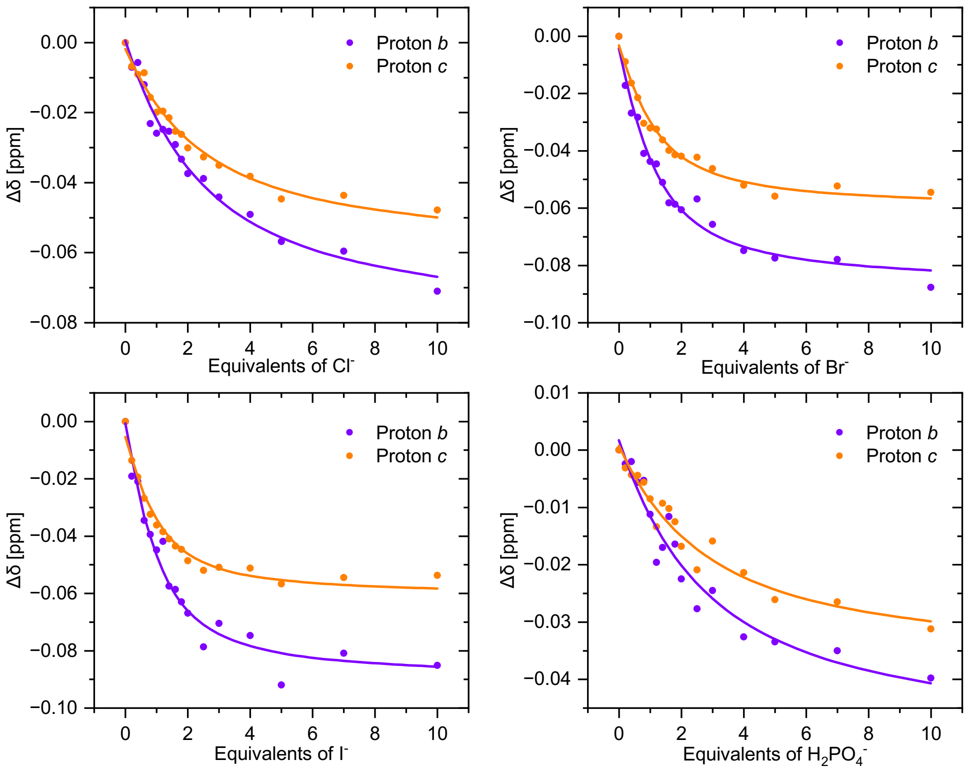

Figure S5.19. Examples of anion binding isotherms for **pDEGA-1·XB** in acetone-d_6:acetonitrile-d_3:D_2O (48.75:48.75:2.5, v/v/v), where circles represent experimental data and the lines represent the fitted isotherm.

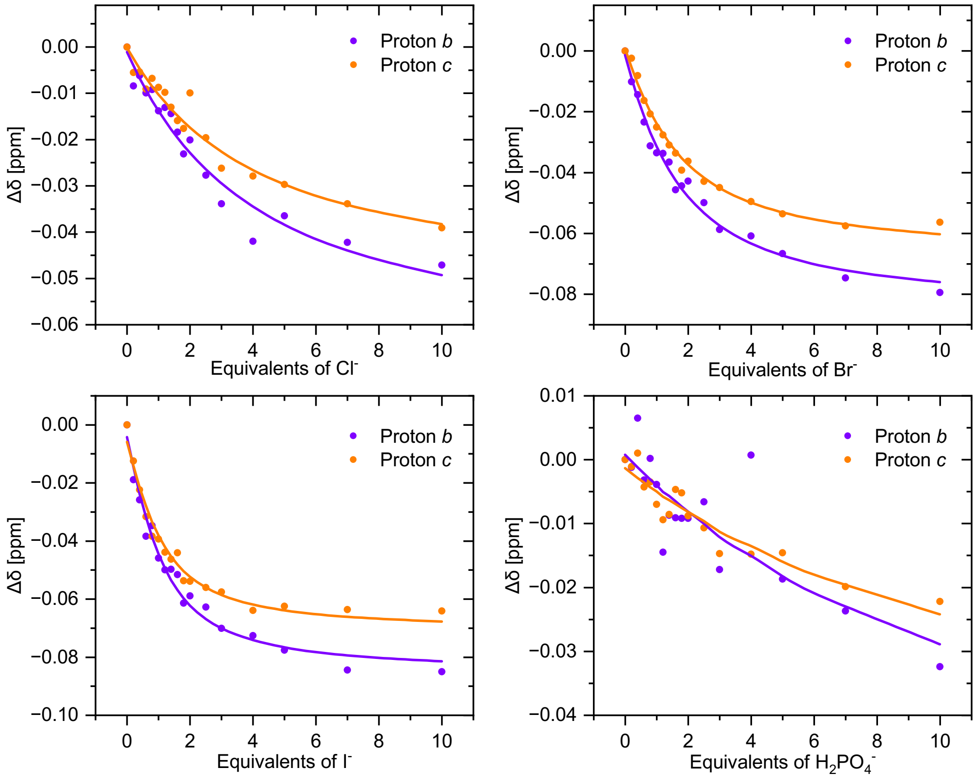

Figure S5.20. Examples of anion binding isotherms for **pDEGA-1·XB** in acetonitrile-d_3:D_2O (97.5:2.5, v/v), where circles represent experimental data and the lines represent the fitted isotherm.

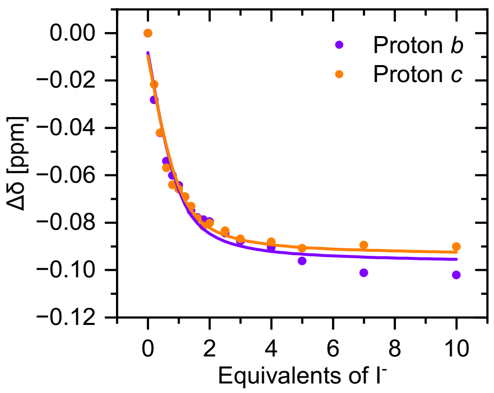

Figure S5.21. Examples of anion binding isotherms for **pDEGA-BuA-1·XB** in acetonitrile-d_3:D_2O (97.5:2.5, v/v), where circles represent experimental data and the lines represent the fitted isotherm.

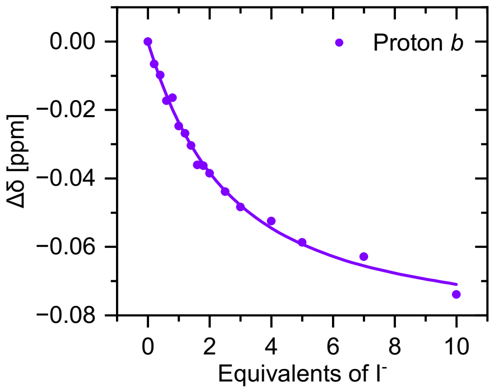

Figure S5.22. Example of anion binding isotherm for **pDEGA-1·XB$_{0.5}$** in acetonitrile-d_3:D_2O (97.5:2.5, v/v), where circles represent experimental data and the lines represent the fitted isotherm.

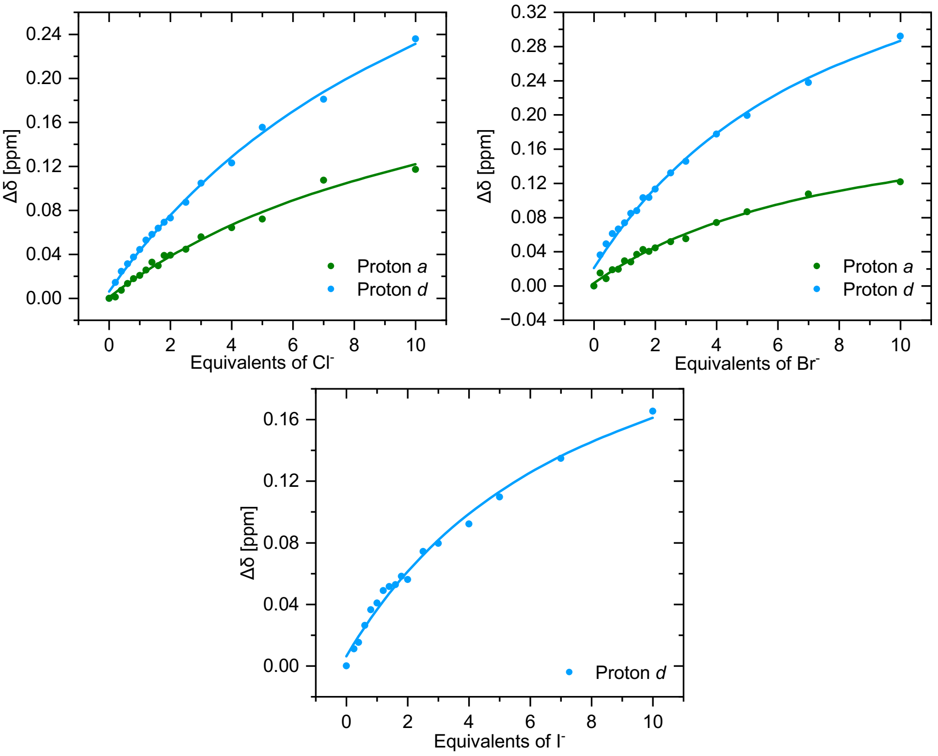

Figure S5.23. Examples of anion binding isotherms for **pDEGA-1·HB** in acetone-d_6:D_2O (97.5:2.5, v/v), where circles represent experimental data and the lines represent the fitted isotherm.

Polymeric enhancements for the HB hosts were comparatively smaller than those exhibited by the XB hosts. This can be rationalised by comparison of the inherently higher hydrophilicity of HB vs. XB motifs, as well as the predictably stronger HB-solvent interactions with the HB acceptor solvents that are used throughout.

Table S5.2. Anion association constants of monomeric receptor **1·HB** and polymeric host **pDEGA-1·HB** determined by ^{1}H NMR titrations in 97.5:2.5 acetone-d_6/D_2O. [a] Errors estimated to be 10%. [b] Errors estimated to be 20%. [c] Errors estimated to be 25%. ND – not determined.

	K (M^{-1})		$K_{pDEGA\text{-}1\cdot HB}/K_{1\cdot HB}$ [c]
	**1·HB**[a]	**pDEGA-1·HB**[b]	
Cl^-	30	65	2.2
Br^-	50	110	2.2
I^-	50	120	2.4
$H_2PO_4^-$	30	ND	ND

Table S5.3. Ratio of anion association constants of polymeric receptors **pDEGA-1·XB** and **pDEGA-1·HB**, representing the halogen bonding enhancement factor ($BEF_{polyXB} = K_{pDEGA\text{-}1\cdot XB}/K_{pDEGA\text{-}1\cdot HB}$) determined by ^{1}H NMR titrations in 97.5:2.5 acetone-d_6/D_2O. Errors estimated to be 30%.

	**BEF_{polyXB} ($K_{pDEGA\text{-}1\cdot XB}/K_{pDEGA\text{-}1\cdot HB}$)**
Cl^-	14
Br^-	20
I^-	25

S6 Discussion of Errors Associated with K by ^{1}H NMR Titrations

Additional analysis was conducted to estimate the error associated with the determined binding constants, and corroborate the numerical results obtained by fitting using Bindfit.

S6.1 Concentration of the Binding Units

The concentration of binding units in each polymer was calculated according to the molecular weight of the polymer and the ratio of binding unit:co-monomer. Molecular weights of the polymers were determined by GPC (see Section S3.2). The ratio of binding unit to co-monomer was fixed by choosing a proper stoichiometry of substrates during polymerisation (i.e. 1:9 [binding unit]:[co-monomer]), and was confirmed by ^{1}H NMR of products (see Section S2.2).

Additionally, the ratio of integrals from polymer and TBA^+ signals (i.e. host:guest) during titrations was used to verify the binding unit concentration, with TBA^+ serving as an internal standard of known concentration. Three well-resolved signals of the polymeric hosts were used for integrations: protons *a*, *b*, and *c′* (the integral of proton *b* was used for normalisation, 2H; protons *a* and *c′* served as a control). At least two signals of TBA^+ were used for comparison with the polymer signals, typically two terminal signals: $-CH_2CH_3$ and/or NCH_2-. To limit the influence of any overlap of the TBA^+ signals with polymer (backbone) signals, all TBA^+ signals were normalised by subtracting the integral of the same region prior to the addition of any TBA^+ salts.

Both the polymer and TBA^+ signals were integrated after each successive TBA^+ salt addition, allowing calculation of the host:TBA^+ ratio for multiple signals at each titration point. The calculated host:TBA^+ ratio value from each signal was then compared to the expected ratio at each titration point (based on initial concentrations), and averaged over all followed signals and titration points to obtain a parameter describing the average deviation from the expected host:guest ratio in a titration (e.g. 1 represents perfect agreement between the expected and determined values, and 1.1 represents an average deviation of 10% from expected). For example, in titrations of monomer **1·XB** (16 additions of TBA^+ salts) all four TBA^+ signals were well resolved, therefore 4·16 = 64 points were compared to polymer signal *b*, and the resulting ratio was averaged to obtain a single value with standard deviation, σ_{N-1}(average deviation from the mean of all signals averaged). The standard error of this average, α was then estimated according to: $\alpha = \frac{\sigma_{N-1}}{\sqrt{N}}$, where N is the sample size (N = 16, the number of titration points). The results for halide titrations of **1·XB** and **pDEGA-1·XB** were collected in Table S6.1.

Importantly, Table S6.1 shows that there is a negligible difference between the error calculated for the polymeric and monomeric host (i.e. both display average deviations of ca. 1.2). This indicates that no additional uncertainty in binding unit concentration of the polymeric hosts compared to the monomeric hosts. The systematic positive deviation of the guest-to-host ratio (value > 1 in all cases) is the result of the difference in relaxation times between aromatic signals of the host and aliphatic signals of positively charged TBA cation, but low concentrations (1 mM regime) likely contribute to the uncertainty of the determined integrals.

Table S6.1. Average deviations from the expected guest-to-host ratio in titrations of monomeric host **1·XB** and polymeric host **pDEGA-1·XB** with halides in different solvent systems. Random error estimated with 95% confidence level.

	Acetone-d_6:D_2O 97.5:2.5		Acetone-d_6: acetonitrile-d_3:D_2O 48.75:48.75:2.5		Acetonitrile-d_3:D_2O 97.5:2.5	
	**1·XB**	**pDEGA-1·XB**	**1·XB**	**pDEGA-1·XB**	**1·XB**	**pDEGA-1·XB**
Cl^-	1.18 ± 0.02	1.16 ± 0.04	1.20 ± 0.02	1.16 ± 0.02	1.17 ± 0.02	1.19 ± 0.04
Br^-	1.33 ± 0.02	1.22 ± 0.04	1.15 ± 0.02	1.23 ± 0.04	1.14 ± 0.02	1.24 ± 0.02
I^-	1.27 ± 0.02	1.18 ± 0.04	1.18 ± 0.02	1.12 ± 0.04	1.12 ± 0.02	1.16 ± 0.02

S6.2 Stability of the Determined Binding Constants

To verify the robustness of results from fittings of the binding isotherms, several tests were conducted which are presented below.

To investigate the error in binding constant, K associated with accurately following the centre of the relevant signals we first carried out a repeat data sampling procedure according to the data processing protocol below:

^{1}H NMR Data Processing Protocol:

1. Manual phase correction was applied to spectrum.
2. All peaks in raw spectrum were referenced to residual solvent peak.
3. Manual baseline correction (Whittaker smoother) was applied to spectrum.
4. Manual exponential apodization (values vary from lb = 0.5-3 Hz) was applied to spectrum.
5. An mNova peak picking protocol was used to determine the chemical shift of each relevant signal to be followed (see Table S5.1 for further details).
6. The obtained shifts were entered into Bindfit software to fit the resulting binding isotherm with a 1:1 host/guest model and determine the associated binding constant. It should be noted that global fitting with parameter sharing was used in each case, and at least two isotherms from the different relevant signals were fitted simultaneously. Within this approach, 5 parameters were fitted (K and chemical shifts of free receptor and host-guest complex) using 17 titration points per signal (degrees of freedom, df = 29).

Steps 1-6 were repeated four times (five independent repeats) for each titration data set, resulting in five binding constants which were then averaged. These results are displayed in Table S6.2 and the standard deviation represents an estimation for the error in K associated with accurately identifying the centre of the relevant signals during data processing, which was determined to be ca. 5% but certainly <10% in all cases. This source of error is unique for the polymeric hosts but resampling in this manner limits the associated uncertainty in processing and provides a better estimate of the binding constants.

Table S6.2. Binding constants of polymeric receptor **pDEGA-1·XB** in response to the halides in different solvent systems obtained from repeated independent processing (resampling five times, N = 1 to 5) of titration data. Standard error reported as a numerical value and percentage of the mean in parentheses.

**Acetonitrile-d$_3$:D$_2$O 97.5:2.5**			
	**I^-**	**Br^-**	**Cl^-**
1	1413	849	191
2	1658	1060	275
3	1614	803	202
4	1627	860	211
5	1436	912	173
Mean	1550	897	210
St. Dev (σ_{N-1})	116	99	39
Standard error of the mean (%)	52 (3.3%)	44 (4.9%)	17 (8.2%)
**Acetone-d$_6$:acetonitrile-d$_3$:D$_2$O 48.75:48.75:2.5**			
	**I^-**	**Br^-**	**Cl^-**
1	2164	1607	305
2	1866	1181	297
3	1576	1197	356
4	1946	1189	327
5	1354	1164	294
Mean	1781	1268	316
St. Dev (σ_{N-1})	318	190	26
Standard error of the mean (%)	140 (8.0%)	85 (6.7%)	12 (3.6%)
**Acetone-d$_6$:D$_2$O 97.5:2.5**			
	**I^-**	**Br^-**	**Cl^-**
1	3030	2204	911
2	2288	2880	1063
3	2808	2746	1137
4	3124	3594	1157
5	3598	2404	1124
Mean	2970	2766	1078
St. Dev (σ_{N-1})	478	535	100
Standard error of the mean (%)	210 (7.2%)	240 (8.7%)	45 (4.1%)

We then investigated the tolerance of K to a systematic error in peak position by introducing random noise to each chemical shift of the followed polymer signals during a titration (0.005 ppm, ca. 5% of the maximum signal shift in a titration). Two exemplary data sets were used, and the analysis was repeated ten times (see Table S6.3). In both cases the average deviation of K from the original, unaltered K is < 17%, which is in good agreement with the predicted errors stated for the binding constants displayed in Table 2 (20%).

Table S6.3. Comparison of the experimentally determined (real) binding constant for **pDEGA-1·XB** and the binding constants determined from the original isotherm with a random error added to the chemical shifts (0.005 ppm) of the followed signals. Deviation from the original value is reported in parentheses as percentage of the original value.

**Binding constants of pDEGA-1·XB and I^- in acetonitrile-d_3/D_2O 97.5:2.5**					
**Real Determined K (M^{-1})**	**Repeat 1**	**Repeat 2**	**Repeat 3**	**Repeat 4**	**Repeat 5**
1887	1478 (22%)	2854 (51%)	1556 (18%)	1771 (6.2%)	2042 (8.2%)
**Average Deviation**	**Repeat 6**	**Repeat 7**	**Repeat 8**	**Repeat 9**	**Repeat 10**
17%	1657 (12%)	1650 (12%)	1415 (25%)	2123 (13%)	1901 (0.7%)
**Binding constants of pDEGA-1·XB and Cl^- in acetone-d_6/D_2O 97.5:2.5**					
**Real Determined K (M^{-1})**	**Repeat 1**	**Repeat 2**	**Repeat 3**	**Repeat 4**	**Repeat 5**
911	934 (2.5%)	972 (6.7%)	1104 (21%)	842 (7.6%)	1194 (31%)
**Average Deviation**	**Repeat 6**	**Repeat 7**	**Repeat 8**	**Repeat 9**	**Repeat 10**
13%	944 (3.6%)	1187 (30%)	859 (5.8%)	975 (7.0%)	1013 (11%)

As discussed above, we are confident that the error associated with extracting data from the broader polymer signals is minimal (ca. 10%). To simulate the effect of these errors on resolved binding affinities, we introduced intentional errors to the concentrations of the host and/or guest (i.e. c_{host} +10% = c_{host} x 1.1) and refitted the binding isotherms according to these values to obtain new, modified binding constants. It should be noted that these intentional errors also in essence take into account any errors in M_n derived from GPC, which would naturally affect the calculated binding unit concentrations. The results are summarised in Tables S6.4 and S6.5 and demonstrate the tolerance of K to these induced errors. Typically, the estimated standard error introduced by uncertainty in concentration is < 20%, and variation in host concentration (most pertinent here) leads to errors of ca. < 10%.

Table S6.4. Comparison of the determined binding constants for **pDEGA-1·XB** and I^- in acetonitrile-d_3/D_2O 97.5:2.5, and the binding constants determined from the original isotherm with an intentional error of 10% or 20% in host and/or guest concentration. Deviation from the original value reported in parentheses as percentage of the original value.

	**K from isotherms with intentional errors added to c_{host} (M^{-1})**			
**Initial K Value**	**c_{host} + 20%**	**c_{host} + 10%**	**c_{host} – 10%**	**c_{host} – 20%**
1887	2191<br>(16%)	2031<br>(7.6%)	1756<br>(6.9%)	1637<br>(13%)
	**K from isotherms with intentional errors added to c_{guest} (M^{-1})**			
	**c_{guest} + 20%**	**c_{guest} + 10%**	**c_{guest} – 10%**	**c_{guest} – 20%**
	1396<br>(26%)	1606<br>(14%)	2276<br>(20%)	2846<br>(51%)
	**K from isotherms with intentional errors added to c_{host} and c_{guest} (M^{-1})**			
	**c_{host} + 10%<br>c_{guest} + 20%**	**c_{host} + 10%<br>c_{guest} + 10%**	**c_{host} + 10%<br>c_{guest} – 10%**	**c_{host} + 10%<br>c_{guest} – 20%**
	1481<br>(22%)	1715<br>(10%)	2476<br>(31%)	3142<br>(66%)
	**c_{host} – 10%<br>c_{guest} + 20%**	**c_{host} – 10%<br>c_{guest} + 10%**	**c_{host} – 10%<br>c_{guest} – 10%**	**c_{host} – 10%<br>c_{guest} – 20%**
	1317<br>(30%)	1507<br>(20%)	2097<br>(11%)	3142<br>(37%)

Table S6.5. Comparison of the determined binding constant for **pDEGA-1·XB** and Cl^- in acetone-d_6/D_2O 97.5:2.5, and the binding constants determined from the original isotherm with an intentional error of 10% or 20% in host and/or guest concentration. Deviation from the original value reported in parentheses as percentage of the original value.

	K from isotherms with intentional errors added to c_{host} (M^{-1})			
**Initial K Value**	c_{host} + 20%	c_{host} + 10%	c_{host} − 10%	c_{host} − 20%
911	993 (9.0%)	951 (4.4%)	874 (4.1%)	839 (7.9%)
	**K from isotherms with intentional errors added to c_{guest} (M^{-1})**			
	c_{guest} + 20%	c_{guest} + 10%	c_{guest} − 10%	c_{guest} − 20%
	708 (22%)	797 (13%)	1062 (17%)	1270 (39%)
	**K from isotherms with intentional errors added to c_{host} and c_{guest} (M^{-1})**			
	c_{host} + 10% c_{guest} + 20%	c_{host} + 10% c_{guest} + 10%	c_{host} + 10% c_{guest} − 10%	c_{host} + 10% c_{guest} − 20%
	733 (20%)	828 (9.1%)	1115 (22%)	1343 (47%)
	c_{host} − 10% c_{guest} + 20%	c_{host} − 10% c_{guest} + 10%	c_{host} − 10% c_{guest} − 10%	c_{host} − 10% c_{guest} − 20%
	685 (25%)	768 (16%)	1013 (11%)	1202 (32%)

S6.3 Repetition of Titration Experiments

Exemplary titrations were independently repeated (i.e. independent solution preparation of the host and guest) and processed using the resampling approach in Section S6.2, the results of which are shown in Table S6.6. This data with the polymeric host **pDEGA-1·XB** shows that all repeated experiments were in excellent agreement with each other (< 10% error in response to the halides).

Table S6.6. Anion binding constant values (M^{-1}) determined for polymeric host **pDEGA-1·XB** in acetonitrile-d_3/D_2O 97.5:2.5 in independent experiments.

	Repeat 1	Repeat 2	Average
Cl^-	210	260	240
Br^-	900	860	880
I^-	1500	1800	1650
$H_2PO_4^-$	56	110	80

Based on all the analysis presented above, we can estimate the error of binding constant values by combining contributions from the resampling analysis (< 10%) and the analysis of concentration perturbations (< 10%). The propagation formula leaves us with an estimate of $u < \sqrt{(10\%)^2 + (10\%)^2} = 14\%$. According to this, we estimate the standard error of determining anion binding constants with this system as a more conservative value of 20%. It is worth stressing here that despite any differences observed with the data processing of the polymeric hosts compared to the monomeric hosts, the analysis resulted in consistent results, e.g. the selectivity trend remained unaltered across different solvent systems (and reflects the trend observed for the monomer).

S7 Role of a Cation in Anion Binding by Polymeric Receptors

As a lipophilic cation, TBA could be, in principle, captured by the polymeric hosts and enhance anion binding due to additional electrostatic interactions. However, we are confident that this is not the case here as none of the TBA ^{1}H NMR signals shift significantly during the titrations of either the monomeric or polymeric hosts in the tested solvent systems. This strongly indicates that TBA does not interact with the studied hosts.

Table S7.1 contains the comparison of chemical shifts of TBA signals at the last titration point of **1·XB** and **pDEGA-1·XB** halide titrations in three solvent systems. It is worth noting that TBA signals are sensitive to the external environment, shown by ≈ 0.2 ppm difference in the chemical shifts of N^+CH_2 signals between acetone/D_2O 97.5:2.5 and acetone/acetonitrile/D_2O 48.75:48.75:2.5, and a further ≈ 0.1 ppm change between acetone/acetonitrile/D_2O 48.75:48.75:2.5 and acetonitrile/D_2O 97.5:2.5. However, in acetone/D_2O 97.5:2.5 and acetonitrile/D_2O 97.5:2.5 there is a negligible difference (< 0.002 ppm) in the chemical shifts of the most sensitive N+CH2 signal during titrations of the monomeric and the polymeric host. This indicates that TBA^+ is in a similar environment with both hosts and is not captured by the polymer. The slightly larger difference in acetone/acetonitrile/D_2O 48.75:48.75:2.5 (≈ 0.02 ppm) is rather a reflection of a larger relative variation in the composition of the solvent system (3 components) between the samples. Altogether these results strongly suggest that the polymeric binding enhancement observed is not caused by TBA capture within the polymeric hosts.

Table S7.1. Chemical shifts (ppm) of selected TBA signals at the last titration point of monomeric host **1·XB** and polymeric host **pDEGA-1·XB** titrations with halides (added as TBA salts) in different solvent systems.

**Acetonitrile-d$_3$:D_2O 97.5:2.5**						
	**I^-**		**Br^-**		**Cl^-**	
	N**CH_2**CH_2-	NCH_2**CH_2**-	N**CH_2**CH_2-	NCH_2**CH_2**-	N**CH_2**CH_2-	NCH_2**CH_2**-
**1·XB**	3.4311	1.8109	3.4326	1.8113	3.4330	1.8116
**pDEGA-1·XB**	3.4314	1.8103	3.4310	1.8105	3.4313	1.8100
**Acetone-d$_6$:Acetonitrile-d$_3$:D_2O 48.75:48.75:2.5**						
	**I^-**		**Br^-**		**Cl^-**	
	N**CH_2**CH_2-	NCH_2**CH_2**-	N**CH_2**CH_2-	NCH_2**CH_2**-	N**CH_2**CH_2-	NCH_2**CH_2**-
**1·XB**	3.2068	1.6704	3.2070	1.6700	3.2016	1.6700
**pDEGA-1·XB**	3.1792	ND	3.1785	1.6424	3.1792	1.6433
**Acetone-d$_6$:D_2O 97.5:2.5**						
	**I^-**		**Br^-**		**Cl^-**	
	N**CH_2**CH_2-	NCH_2**CH_2**-	N**CH_2**CH_2-	NCH_2**CH_2**-	N**CH_2**CH_2-	NCH_2**CH_2**-
**1·XB**	3.0708	1.5875	3.0709	1.5871	3.0997	1.6164
**pDEGA-1·XB**	3.0705	1.5872	3.0708	1.5880	3.0714	1.5880

To further study the role of the counter cation, an additional titration of **pDEGA-1·XB** with NaI in acetonitrile-d$_3$/D_2O was conducted. Unfortunately, a precipitate was observed when > 5 equiv. of NaI was added, preventing further studies. Data collected up to this point was used for fitting to obtain a binding constant of $K_{\mathbf{pDEGA\text{-}1\cdot XB}}$(NaI) = 1350 ± 270 M^{-1} (Figure S7.1), which is close to the one determined using TBAI, $K_{\mathbf{pDEGA\text{-}1\cdot XB}}$(TBAI) = 1650 ± 330 M^{-1}. Based on the analysis presented in this chapter we can confidently rule out a significant effect of cation on anion binding by the polymeric hosts.

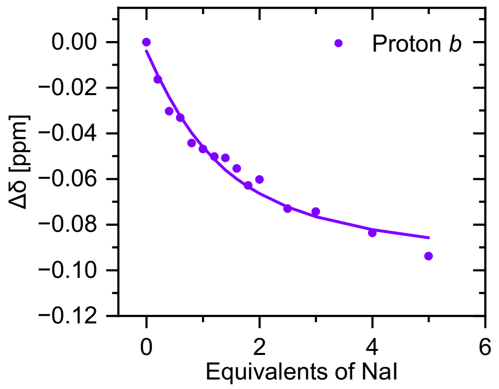

Figure S7.1. Example of iodide (added as NaI) binding isotherm for **pDEGA-1·XB** in acetonitrile-d$_3$:D_2O (97.5:2.5, v/v), where circles represent experimental data and the lines represent the fitted isotherm.

S8 Electrochemical Characterisation

S8.1 Electrochemical Characterisation of Hosts in 97.5:2.5 acetone/H_2O

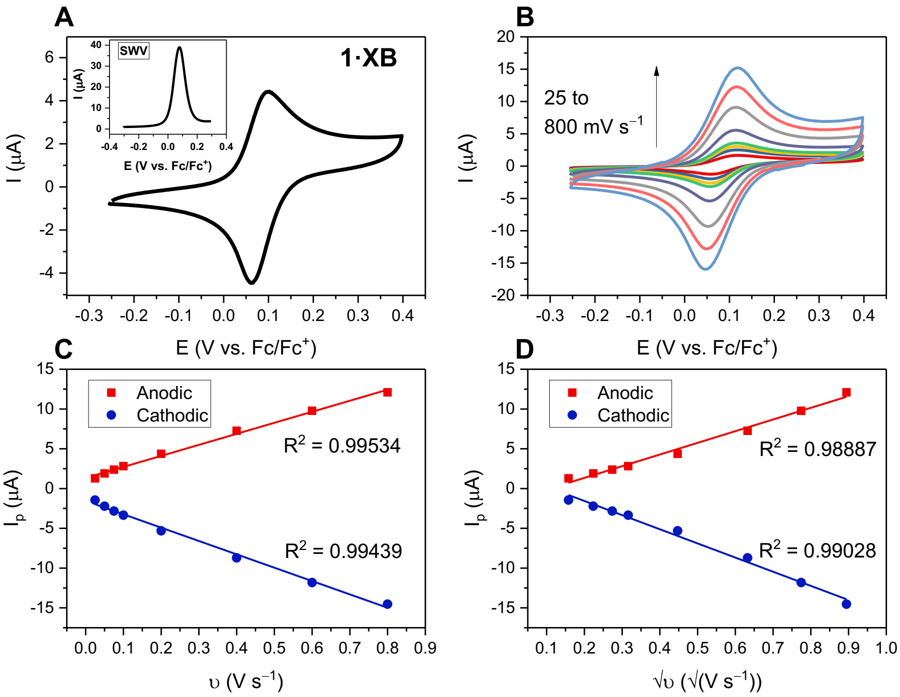

Figure S8.1. A) Cyclic voltammogram and square wave voltammogram (inset) of 0.1 mM **1·XB** in 97.5: 2.5 acetone/H_2O, 100 mM $TBAClO_4$, at a scan rate of 0.1 V s^{-1}. B) Cyclic voltammograms of the same system at varying scan rates from 0.025 to 0.8 V s^{-1}, and plots of peak current vs. C) scan rate D) the square-root of the scan rate in the same solvent system. All potentials wrt. external Fc|Fc^+ couple.

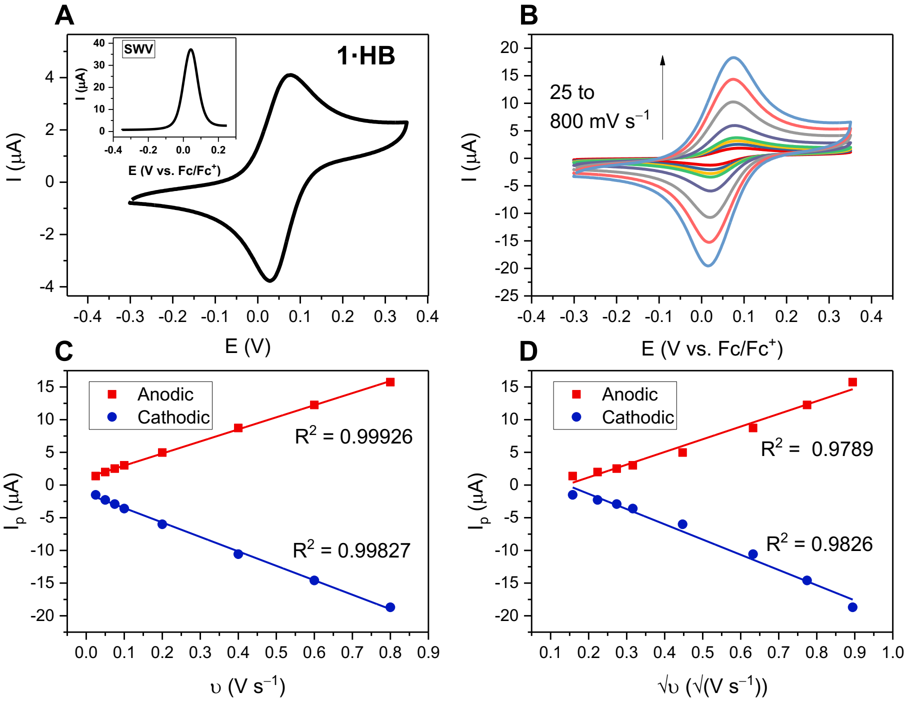

Figure S8.2. A) Cyclic voltammogram and square wave voltammogram (inset) of 0.1 mM **1·HB** in 97.5: 2.5 acetone/H_2O, 100 mM $TBAClO_4$, at a scan rate of 0.1 V s^{-1}. B) Cyclic voltammograms of the same system at varying scan rates from 0.025 to 0.8 V s^{-1}, and plots of peak current vs. C) scan rate D) the square-root of the scan rate in the same solvent system. All potentials wrt. external Fc|Fc^+ couple.

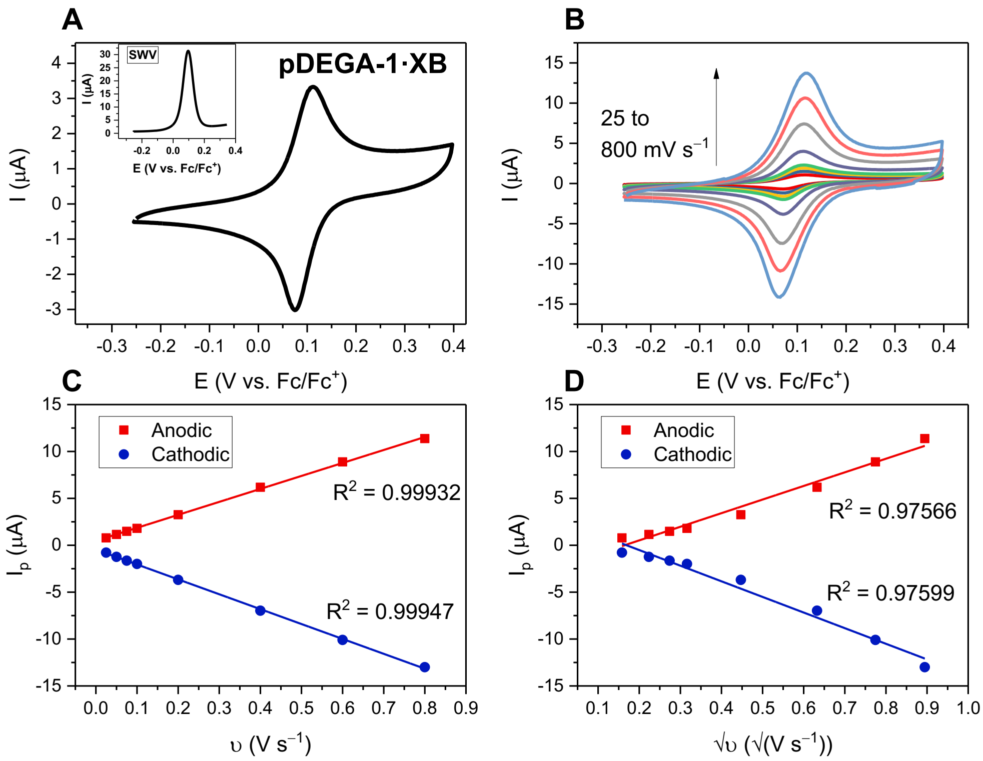

Figure S8.3. A) Cyclic voltammogram and square wave voltammogram (inset) of 0.1 mM **pDEGA-1·XB** in 97.5: 2.5 acetone/H_2O, 100 mM $TBAClO_4$, at a scan rate of 0.1 V s^{-1}. B) Cyclic voltammograms of the same system at varying scan rates from 0.025 to 0.8 V s^{-1}, and plots of peak current vs. C) scan rate D) the square-root of the scan rate in the same solvent system. All potentials wrt. external Fc|Fc^+ couple.

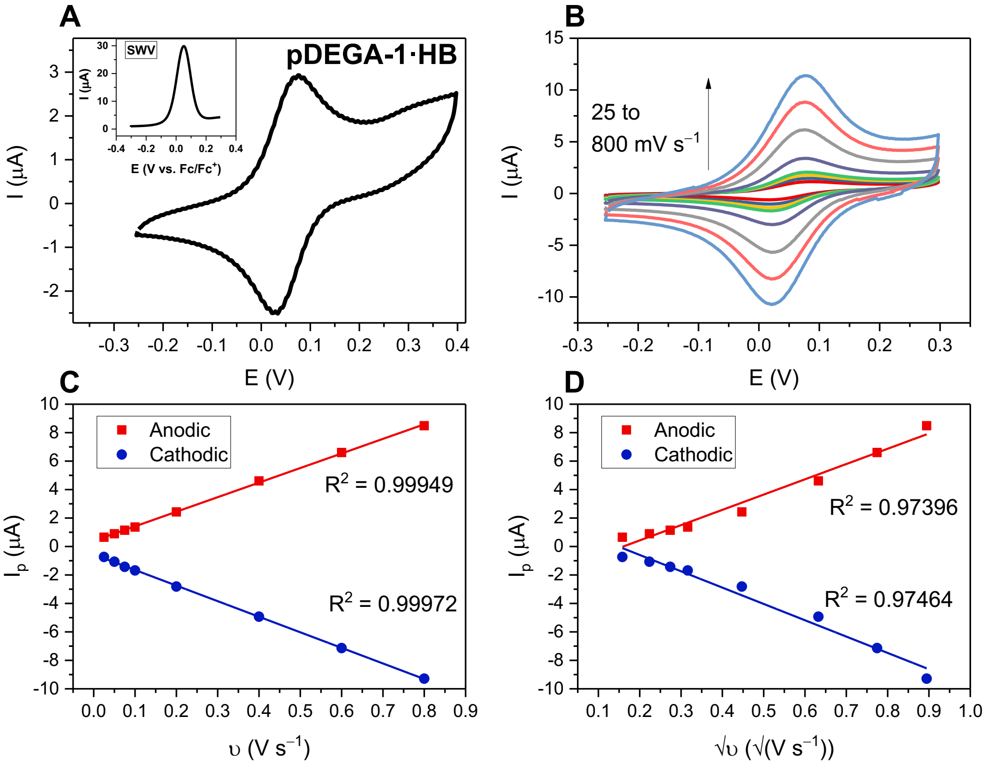

Figure S8.4. A) Cyclic voltammogram and square wave voltammogram (inset) of 0.1 mM **pDEGA-1·HB** in 97.5: 2.5 acetone/H_2O, 100 mM $TBAClO_4$, at a scan rate of 0.1 V s^{-1}. B) Cyclic voltammograms of the same system at varying scan rates from 0.025 to 0.8 V s^{-1}, and plots of peak current vs. C) scan rate D) the square-root of the scan rate in the same solvent system. All potentials wrt. external Fc|Fc^+ couple.

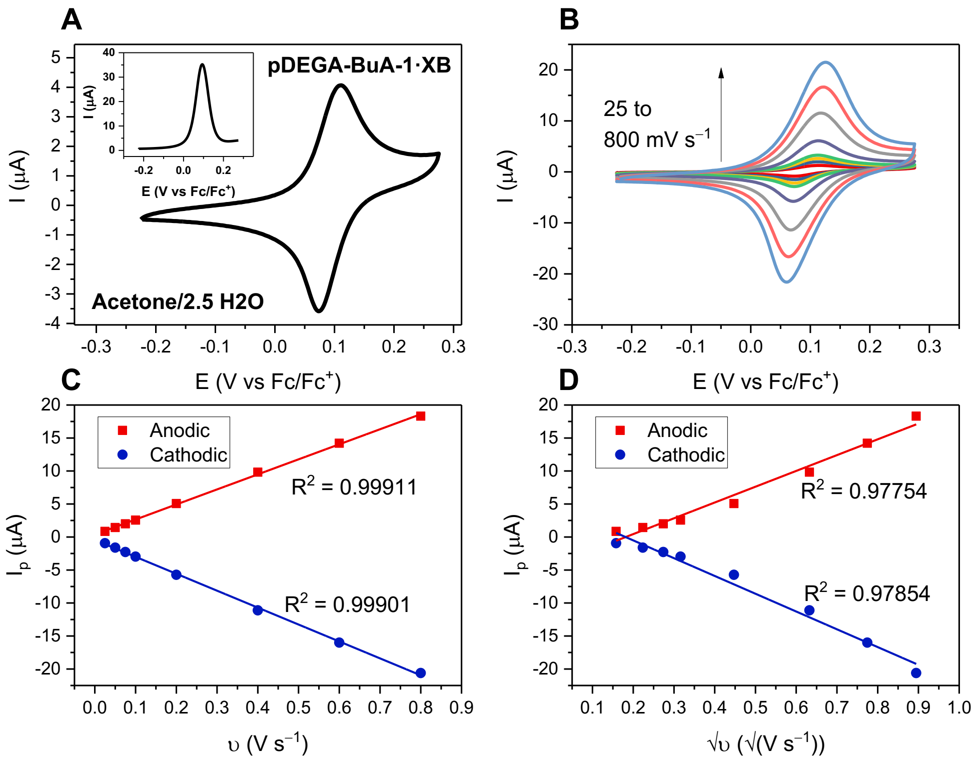

Figure S8.5. A) Cyclic voltammogram and square wave voltammogram (inset) of 0.1 mM **pDEGA-BuA-1·XB** in 97.5: 2.5 acetone/H_2O, 100 mM $TBAClO_4$, at a scan rate of 0.1 V s^{-1}. B) Cyclic voltammograms of the same system at varying scan rates from 0.025 to 0.8 V s^{-1}, and plots of peak current vs. C) scan rate D) the square-root of the scan rate in the same solvent system. All potentials wrt. external Fc|Fc^+ couple.

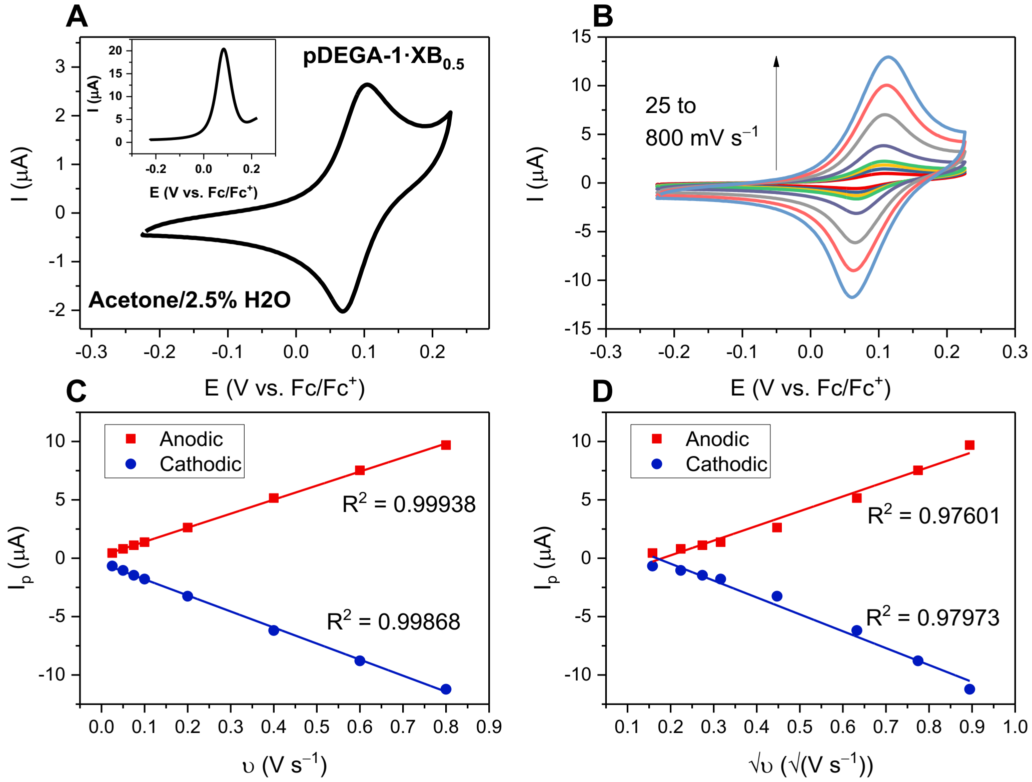

Figure S8.6. A) Cyclic voltammogram and square wave voltammogram (inset) of 0.1 mM **pDEGA-1·XB$_{0.5}$** in 97.5: 2.5 acetone/H_2O, 100 mM $TBAClO_4$, at a scan rate of 0.1 V s^{-1}. B) Cyclic voltammograms of the same system at varying scan rates from 0.025 to 0.8 V s^{-1}, and plots of peak current vs. C) scan rate D) the square-root of the scan rate in the same solvent system. All potentials wrt. external Fc|Fc^+ couple.

S8.2 Electrochemical Characterisation of Hosts in 97.5:2.5 ACN/H_2O

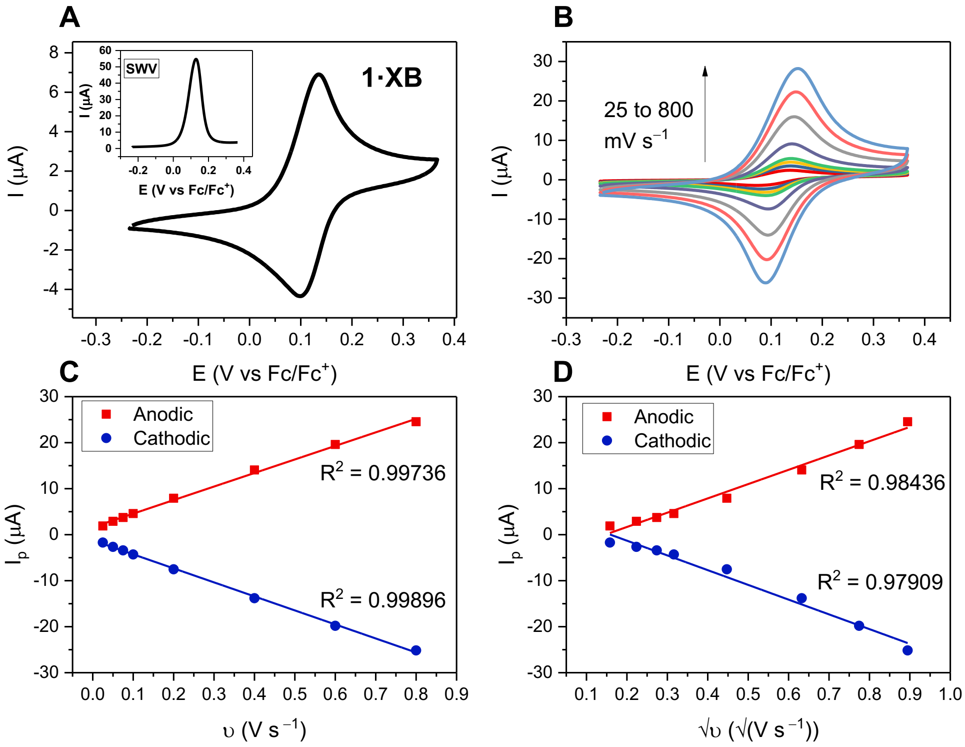

Figure S8.7. A) Cyclic voltammogram and square wave voltammogram (inset) of 0.1 mM **1·XB** in 97.5: 2.5 ACN/H_2O, 100 mM $TBAClO_4$, at a scan rate of 0.1 V s^{-1}. B) Cyclic voltammograms of the same system at varying scan rates from 0.025 to 0.8 V s^{-1}, and plots of peak current vs. C) scan rate D) the square-root of the scan rate in the same solvent system. All potentials wrt. external Fc|Fc^+ couple.

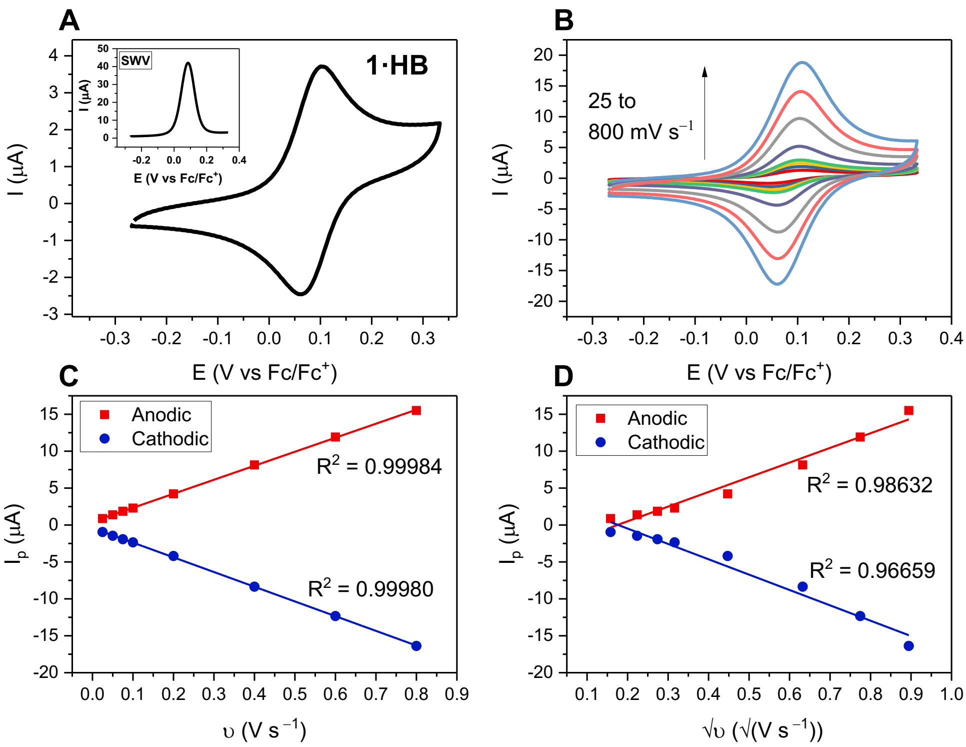

Figure S8.8. A) Cyclic voltammogram and square wave voltammogram (inset) of 0.1 mM **1·HB** in 97.5: 2.5 ACN/H_2O, 100 mM $TBAClO_4$, at a scan rate of 0.1 V s^{-1}. B) Cyclic voltammograms of the same system at varying scan rates from 0.025 to 0.8 V s^{-1}, and plots of peak current vs. C) scan rate D) the square-root of the scan rate in the same solvent system. All potentials wrt. external Fc|Fc^+ couple.

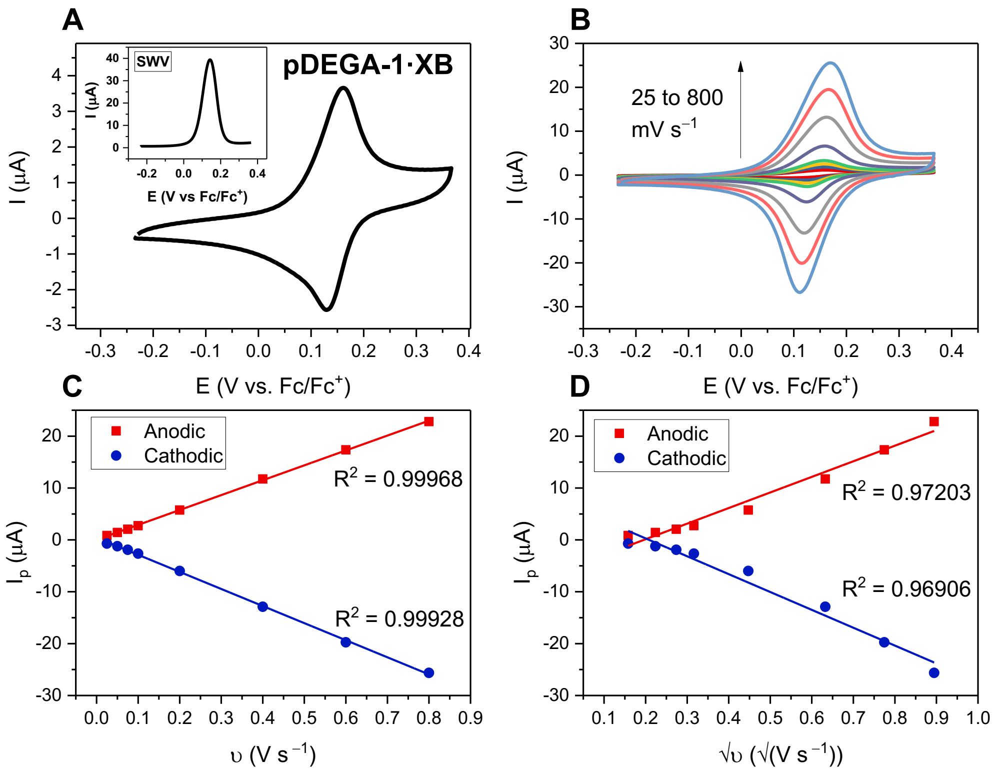

Figure S8.9. A) Cyclic voltammogram and square wave voltammogram (inset) of 0.1 mM **pDEGA-1·XB** in 97.5: 2.5 ACN/H_2O, 100 mM $TBAClO_4$, at a scan rate of 0.1 V s^{-1}. B) Cyclic voltammograms of the same system at varying scan rates from 0.025 to 0.8 V s^{-1}, and plots of peak current vs. C) scan rate D) the square-root of the scan rate in the same solvent system. All potentials wrt. external Fc|Fc^+ couple.

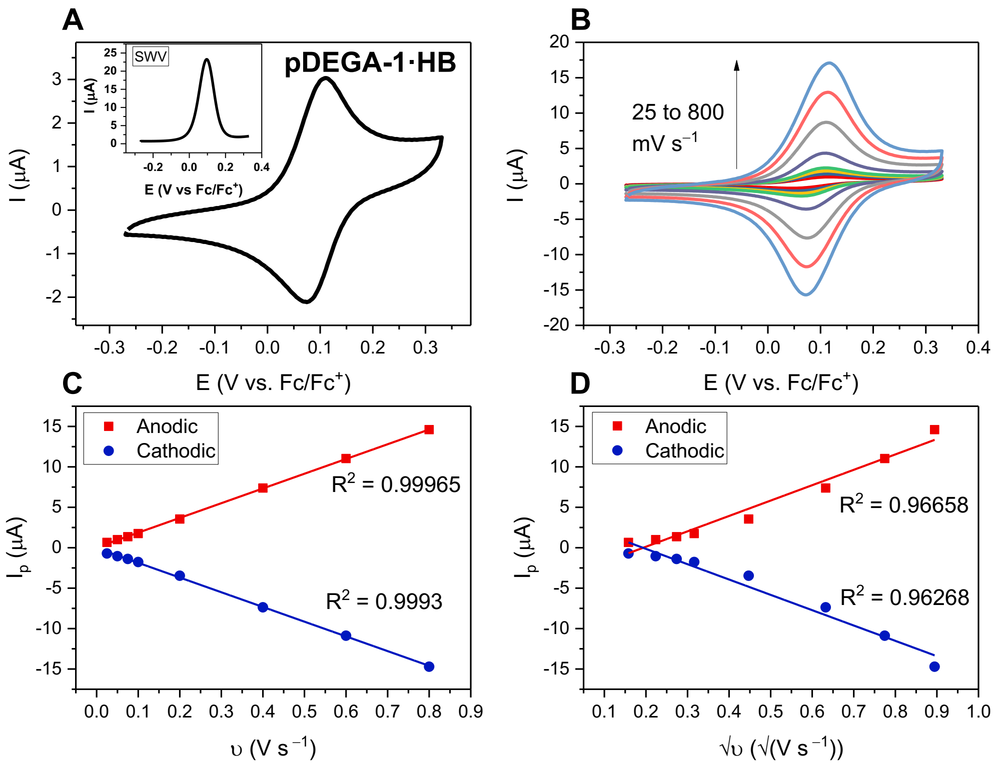

Figure S8.10. A) Cyclic voltammogram and square wave voltammogram (inset) of 0.1 mM **pDEGA-1·HB** in 97.5: 2.5 ACN/H_2O, 100 mM $TBAClO_4$, at a scan rate of 0.1 V s^{-1}. B) Cyclic voltammograms of the same system at varying scan rates from 0.025 to 0.8 V s^{-1}, and plots of peak current vs. C) scan rate D) the square-root of the scan rate in the same solvent system. All potentials wrt. external Fc|Fc^+ couple.

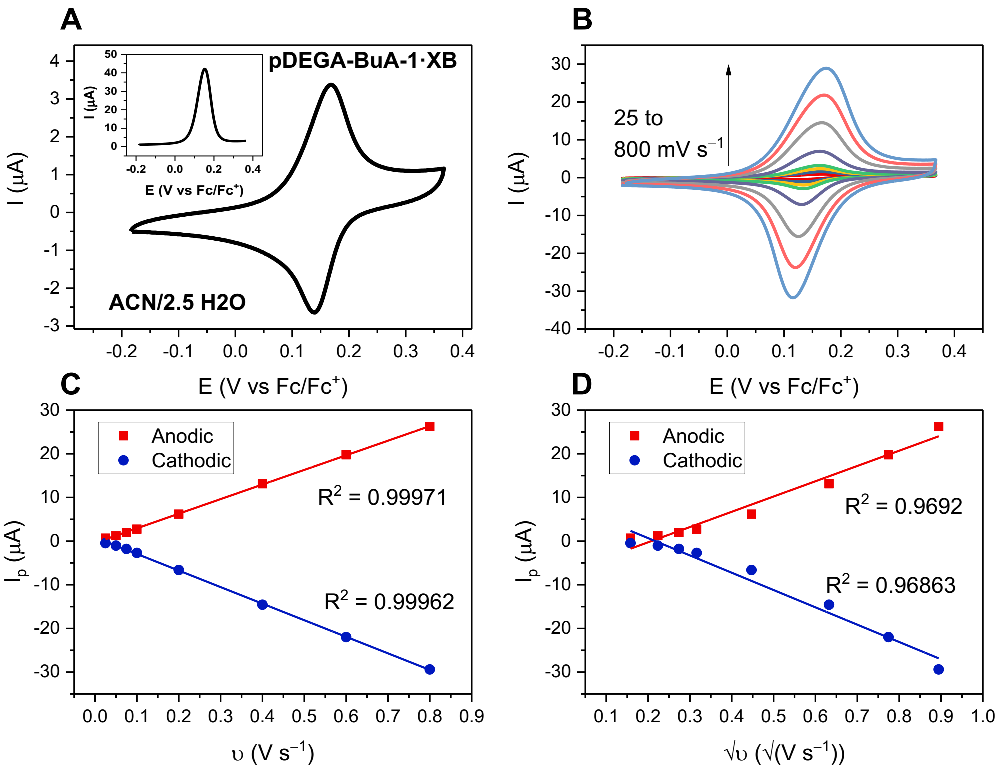

Figure S8.11. A) Cyclic voltammogram and square wave voltammogram (inset) of 0.1 mM **pDEGA-BuA-1·XB** in 97.5: 2.5 ACN/H_2O, 100 mM $TBAClO_4$, at a scan rate of 0.1 V s^{-1}. B) Cyclic voltammograms of the same system at varying scan rates from 0.025 to 0.8 V s^{-1}, and plots of peak current vs. C) scan rate D) the square-root of the scan rate in the same solvent system. All potentials wrt. external Fc|Fc^+ couple.

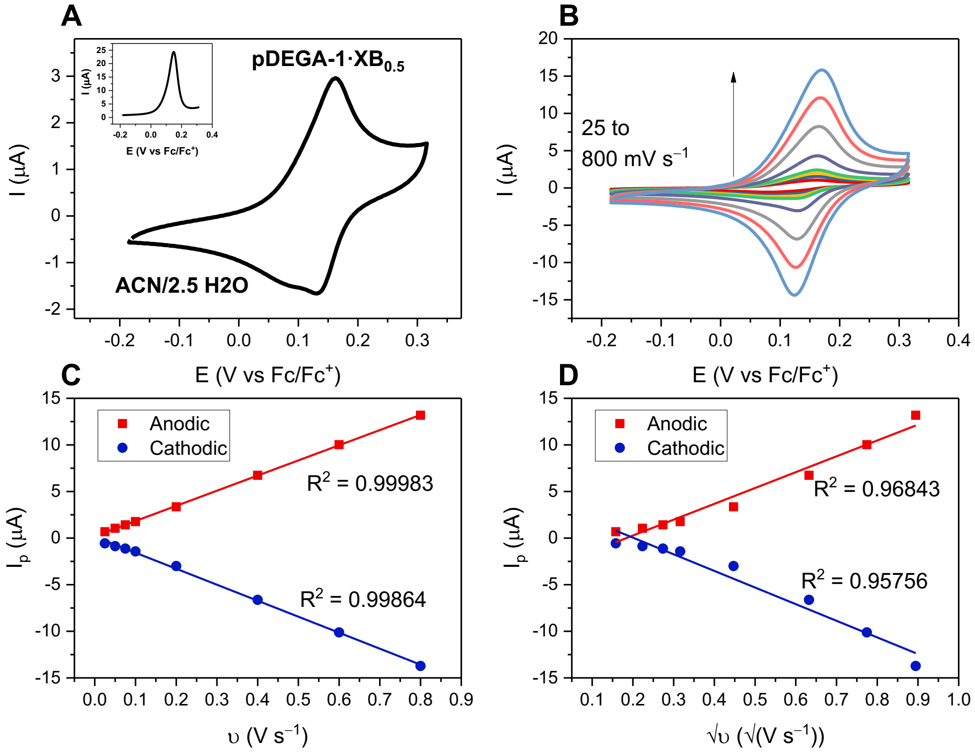

Figure S8.12. A) Cyclic voltammogram and square wave voltammogram (inset) of 0.1 mM **pDEGA-1·XB$_{0.5}$** in 97.5: 2.5 ACN/H_2O, 100 mM $TBAClO_4$, at a scan rate of 0.1 V s^{-1}. B) Cyclic voltammograms of the same system at varying scan rates from 0.025 to 0.8 V s^{-1}, and plots of peak current vs. C) scan rate D) the square-root of the scan rate in the same solvent system. All potentials wrt. external Fc|Fc^+ couple.

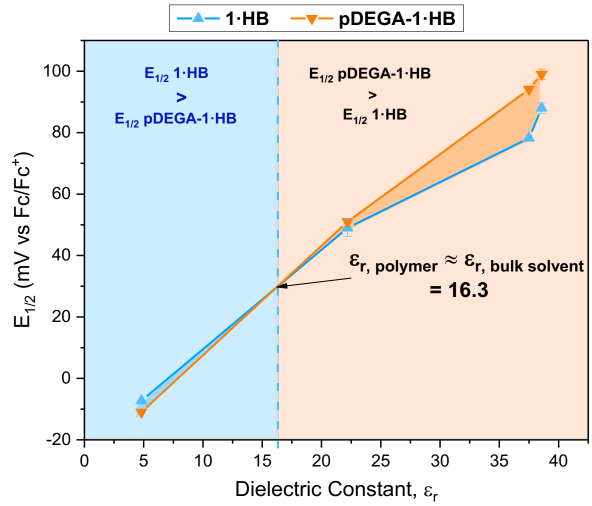

Figure S8.13. Half wave potentials of **pDEGA-1·HB** and **1·HB** against bulk solvent polarity. Connecting lines are shown to guide the eye only.

A similar difference in $E_{1/2}$ as resolved for the XB hosts was determined between **pDEGA-1·HB** and **1·HB** (99 ± 2 mV *vs*. 88 ± 2 mV, respectively). Interestingly, the relative difference between XB and HB hosts is generally larger here compared to previous studies (41 vs 14 mV difference for **1·XB/HB** *vs*. a similar bis-Fc XB/HB receptor in ACN).[4] This relative enhancement was attributed to the direct linkage/conjugation (without a methyl spacer) of the Fc transducer unit to the electron-withdrawing (iodo)triazole motif, which disproportionately affected the XB sensors more than their HB analogues (because the C-I bond is inherently more polarisable). The local dielectric around the binding unit in a polymer is a combination of material contributions (i.e. the dielectric properties of the co-monomer) and the extent of solvent penetration; it is not surprising, then, that the crossover of $E_{1/2}$s *vs.* ε_r occurs at different dielectric values for the **pDEGA-1·XB/1·XB** pair and the **pDEGA-1·HB/1·HB** pair, with the latter seemingly more accessible to solvents with strong HB accepting capabilities such as acetone, ACN, and water, making the local environment more polar in comparison to **pDEGA-1·XB**.

S9 Electrochemical Sensing Data

S9.1 Electrochemical Anion Sensing Performance of 1·HB and pDEGA-1·HB

As observed with the XB hosts, significant voltammetric perturbations of the $E_{1/2}$s of both **1·HB** and **pDEGA-1·HB** were observed in response to a range of anions in different organic/aqueous solvent mixtures. The HB hosts generally exhibited a slight selectivity trend for the oxoanions HSO_4^- and $H_2PO_4^-$ over the halides, which is consistent with previous comparative studies with similar XB/HB sensors. Importantly, the polymeric enhancement observed for the XB hosts was also observed for **pDEGA-1·HB/1·HB**, supporting the analogous enhancements observed by ^{1}H-NMR which is again attributed to the comparatively lower dielectric of the surrounding DEGA comonomer units somewhat shielding the microenvironment around each binding unit, resulting in a lower local dielectric and augmented anion binding.

Table S9.1. Change in $E_{1/2}$ of monomeric receptors **1·XB/HB**, and polymeric receptors **pDEGA-1·XB/HB** in response to a range of anions (HSO_4^-, $H_2PO_4^-$, Cl^- and Br^-), as determined by SWV titrations in 97.5:2.5 acetone:H_2O. Errors estimated to be ±5 mV.

	ΔE_{max} (mV)			
Anion	**1·XB**	**pDEGA-1·XB**	**1·HB**	**pDEGA-1·HB**
HSO_4^-	–77.4	**–97.7**	–106	**–131**
$H_2PO_4^-$	–157	**–201**	–188	**–211**
Cl^-	–53.6	**–75.4**	–51.6	**–85.3**
Br^-	–45.6	**–74.4**	–43.6	**–49.6**

Table S9.2. Change in $E_{1/2}$ of monomeric receptors **1·XB/HB**, and polymeric receptors **pDEGA-1·XB/HB** in response to a range of anions (HSO_4^-, $H_2PO_4^-$, Cl^- and Br^-), as determined by SWV titrations in 97.5:2.5 ACN:H_2O. Errors estimated to be ±5 mV.

	ΔE_{max} (mV)			
Anion	**1·XB**	**pDEGA-1·XB**	**1·HB**	**pDEGA-1·HB**
HSO_4^-	–53.6	**–76.4**	–86.3	**–91.3**
$H_2PO_4^-$	–115	**–147**	–133	**–168**
Cl^-	–53.6	**–69.4**	–33.7	**–41.7**
Br^-	–51.6	**–53.6**	–27.8	**–34.7**

Table S9.3. Ratio of maximum change in $E_{1/2}$ (ΔE_{max}) of polymeric hosts **pDEGA-1·XB/HB** and monomeric receptors **1·XB/HB**, representing the polymeric enhancement factor (PEF = $\Delta E_{max,\ poly}/\Delta E_{max,mon}$ determined by SWV titrations in 97.5:2.5 acetone/H_2O, and 97.5:2.5 ACN/H_2O. Errors estimated to be 10%.

	Polymeric Enhancement Factor ($\Delta E_{max,\ poly}/\Delta E_{max,mon}$)			
	97.5:2.5 acetone:H_2O		97.5:2.5 ACN:H_2O	
	**pDEGA-1·XB/1·XB**	**pDEGA-1·HB/1·HB**	**pDEGA-1·XB/1·XB**	**pDEGA-1·HB/1·HB**
HSO_4^-	1.3	1.2	1.3	1.1
$H_2PO_4^-$	1.3	1.1	1.3	1.3
Cl^-	1.4	1.7	1.3	1.2
Br^-	1.6	1.1	1.0	1.2

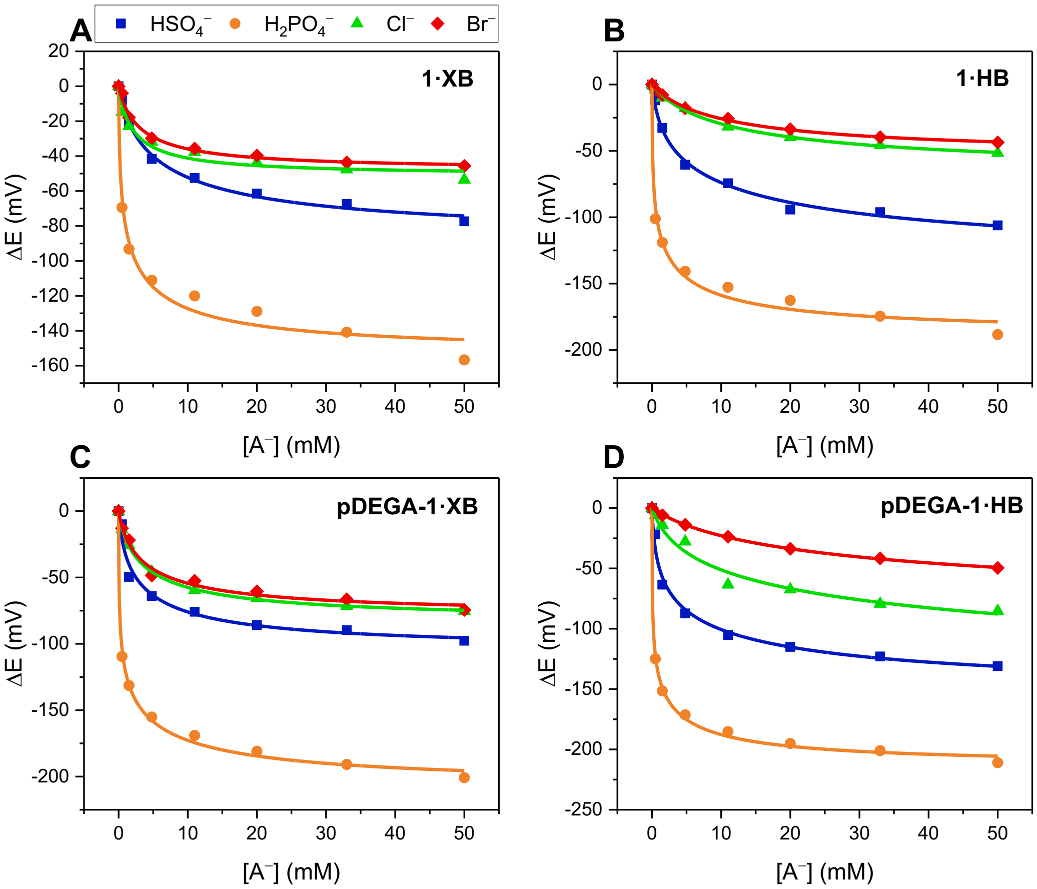

Figure S9.1. Response isotherms in 97.5:2.5 acetone/H_2O of A) **1·XB** B) **1·HB** C) **pDEGA-1·XB** and D) **pDEGA-1·HB** in response to increasing concentrations of HSO_4^- (blue), $H_2PO_4^-$ (orange), Cl^- (green) and Br^- (red) up to 50 mM. All isotherms were fitted to a derived Nernst model (Eqn. S2).

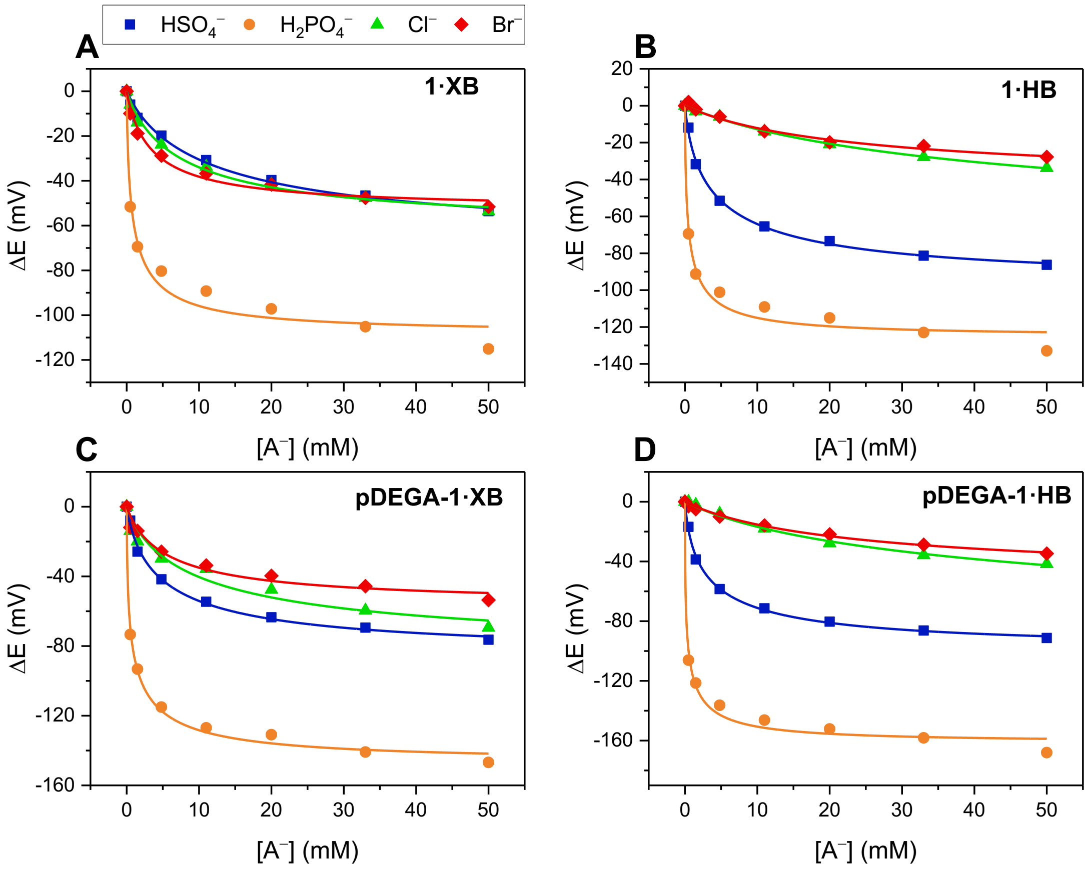

Figure S9.2. Response isotherms in 97.5:2.5 ACN/H_2O of A) **1·XB** B) **1·HB** C) **pDEGA-1·XB** and D) **pDEGA-1·HB** in response to increasing concentrations of HSO_4^- (blue), $H_2PO_4^-$ (orange), Cl^- (green) and Br^- (red) up to 50 mM. All isotherms were fitted to a derived Nernst model (Eqn. S2).

S9.2 NMR vs Echem Anion Binding Constants

Table S9.4. Comparison of the K_a values for **1·XB** and **pDEGA-1·XB** determined from ^{1}H NMR and voltammetric titrations (K_{red}, as determined from fitting isotherms with a 1:1 host-guest Nernst model (K_{Ox}/K_{red}, Eqn. 2) in 97.5:2.5 acetone:H_2O (deuterated for NMR titrations).

	1·XB		pDEGA-1·XB	
	NMR	Echem	NMR	Echem
HSO_4^-	N/A	31	N/A	46
$H_2PO_4^-$	60	59	410	36
Cl^-	170	170	910	55
Br^-	470	124	2200	58

Table S9.5. Comparison of the K_a values for **1·XB** and **pDEGA-1·XB** determined from ^{1}H NMR and voltammetric titrations (K_{red}, as determined from fitting isotherms with a 1:1 host-guest Nernst model (K_{Ox}/K_{red}, Eqn. 2) in 97.5:2.5 ACN:H_2O (deuterated for NMR titrations).

	1·XB		pDEGA-1·XB	
	NMR	Echem	NMR	Echem
HSO_4^-	N/A	22	N/A	38
$H_2PO_4^-$	10	159	80	95
Cl^-	20	38.5	230	18
Br^-	60	101	840	58

S9.3 pDEGA Homopolymer Control

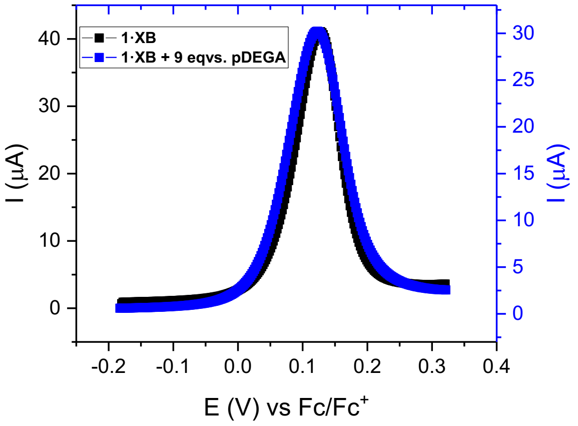

Figure S9.3. Square wave voltammograms of 0.1 mM **1·XB** (black line) and 0.1 mM **1·XB** + 9 eqvs. **pDEGA** (blue line) in 97.5: 2.5 ACN/H_2O with 100 mM $TBAClO_4$ as supporting electrolyte.

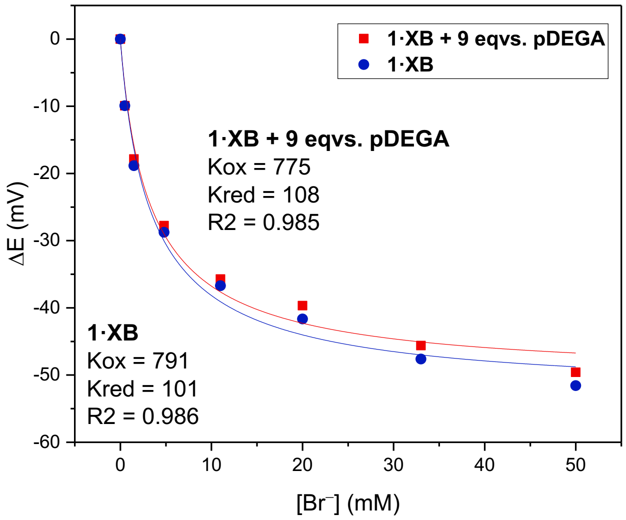

Figure S9.4. Response isotherms in 97.5:2.5 ACN/H_2O of **1·XB** (blue) and **1·XB** + 9 eqvs. **pDEGA** (red) in response to increasing concentrations of Br^- up to 50 mM. All isotherms were fitted to a derived Nernst model (Eqn. S2).

S9.4 Tabulated Nernst Binding Isotherm Data

Table S9.6. Diffusive binding constants of the oxidised (K_{Ox}) and neutral (K_{red}) hosts (M^{-1}), and BEFs in response to HSO_4^-, as determined from fitting isotherms with a 1:1 host-guest Nernst model (K_{Ox}/K_{red}, Eqn. 2) or the maximum shift (Eqn. 1) in response to 50 mM [X^-].

HSO_4^- Host	Solvent	K_{ox} (M^{-1})	K_{red} (M^{-1})	R^2	BEF (isotherm)	BEF (max shift)
**1·XB**	97.5:2.5 ACN/H_2O	301 ± 35.4	21.9 ± 6.26	0.992	13.7	8.05
**pDEGA-1·XB**	97.5:2.5 ACN/H_2O	1040 ± 84.0	37.9 ± 6.61	0.996	27.4	19.6
**1·HB**	97.5:2.5 ACN/H_2O	1580 ± 91.0	37.1 ± 4.85	0.998	42.5	28.8
**pDEGA-1·HB**	97.5:2.5 ACN/H_2O	2280 ± 121	48.3 ± 5.35	0.998	47.2	34.9
**1·XB**	97.5:2.5 Acetone/H_2O	903 ± 81.5	30.8 ± 6.54	0.995	29.3	20.3
**pDEGA-1·XB**	97.5:2.5 Acetone/H_2O	2740 ± 585	46.5 ± 21.3	0.975	58.9	44.8
**1·HB**	97.5:2.5 Acetone/H_2O	1860 ± 191	9.41 ± 5.08	0.994	197	62.4
**pDEGA-1·HB**	97.5:2.5 Acetone/H_2O	5703 ± 887	14.3 ± 9.36	0.987	339	164

Table S9.7. Diffusive binding constants of the oxidised (K_{Ox}) and neutral (K_{red}) hosts (M^{-1}), and BEFs in response to $H_2PO_4^-$, as determined from fitting isotherms with a 1:1 host-guest Nernst model (K_{Ox}/K_{red}, Eqn. 2) or the maximum shift (Eqn. 1) in response to 50 mM [X^-].

$H_2PO_4^-$ Host	Solvent	K_{ox} (M^{-1})	K_{red} (M^{-1})	R^2	BEF (isotherm)	BEF (max shift)
**1·XB**	97.5:2.5 ACN/H_2O	10800 ± 2490	159 ± 56.8	0.973	68.0	88.3
**pDEGA-1·XB**	97.5:2.5 ACN/H_2O	23100 ± 3140	95.3 ± 18.7	0.995	305	304
**1·HB**	97.5:2.5 ACN/H_2O	26900 ± 5700	203 ± 64.5	0.982	132	177
**pDEGA-1·HB**	97.5:2.5 ACN/H_2O	104000 ± 12200	193 ± 57.1	0.996	543	696
**1·XB**	97.5:2.5 Acetone/H_2O	22800 ± 4700	59.5 ± 25.9	0.981	383	447
**pDEGA-1·XB**	97.5:2.5 Acetone/H_2O	115000 ± 12200	35.6 ± 10.1	0.996	3220	2490
**1·HB**	97.5:2.5 Acetone/H_2O	72400 ± 13200	47.5 ± 20.2	0.988	1520	1540
**pDEGA-1·HB**	97.5:2.5 Acetone/H_2O	242000 ± 22600	59.3 ± 11.9	0.998	4080	3710

Table S9.8. Diffusive binding constants of the oxidised (K_{Ox}) and neutral (K_{red}) hosts (M^{-1}), and BEFs in response to Cl^-, as determined from fitting isotherms with a 1:1 host-guest Nernst model (K_{Ox}/K_{red}, Eqn. 2) or the maximum shift (Eqn. 1) in response to 50 mM [X^-].

Cl^- Host	Solvent	K_{ox} (M^{-1})	K_{red} (M^{-1})	R^2	BEF (isotherm)	BEF (max shift)
**1·XB**	97.5:2.5 ACN/H_2O	421 ± 44.1	38.5 ± 7.73	0.993	10.9	8.05
**pDEGA-1·XB**	97.5:2.5 ACN/H_2O	472 ± 131	18.4 ± 14.7	0.948	25.6	14.9
**1·HB**	97.5:2.5 ACN/H_2O	71.4 ± 4.19	4.34 ± 1.40	0.999	16.4	3.72
**pDEGA-1·HB**	97.5:2.5 ACN/H_2O	96.4 ± 9.81	2.15 ± 2.48	0.995	44.9	5.06
**1·XB**	97.5:2.5 Acetone/H_2O	1240 ± 289	170 ± 52.9	0.966	7.33	8.05
**pDEGA-1·XB**	97.5:2.5 Acetone/H_2O	1360 ± 48.6	54.7 ± 3.72	0.999	24.8	18.8
**1·HB**	97.5:2.5 Acetone/H_2O	285 ± 20.0	21.5 ± 3.67	0.997	13.3	7.45
**pDEGA-1·HB**	97.5:2.5 Acetone/H_2O	650 ± 131	1.80 ± 7.00	0.977	361.5	6.90

Table S9.9. Diffusive binding constants of the oxidised (K_{Ox}) and neutral (K_{red}) hosts (M^{-1}), and BEFs in response to Br^-, as determined from fitting isotherms with a 1:1 host-guest Nernst model (K_{Ox}/K_{red}, Eqn. 2) or the maximum shift (Eqn. 1) in response to 50 mM [X^-].

Br^- Host	Solvent	K_{ox} (M^{-1})	K_{red} (M^{-1})	R^2	BEF (isotherm)	BEF (max shift)
**1·XB**	97.5:2.5 ACN/H_2O	791 ± 122	101 ± 22.8	0.986	7.84	7.45
**pDEGA-1·XB**	97.5:2.5 ACN/H_2O	519 ± 121	58.1 ± 22.5	0.967	8.94	8.05
**1·HB**	97.5:2.5 ACN/H_2O	88.0 ± 19.0	17.1 ± 7.84	0.983	5.13	2.95
**pDEGA-1·HB**	97.5:2.5 ACN/H_2O	106 ± 14.0	13.5 ± 4.64	0.992	7.86	3.86
**1·XB**	97.5:2.5 Acetone/H_2O	810 ± 99.4	124.4 ± 21.1	0.992	6.51	5.91
**pDEGA-1·XB**	97.5:2.5 Acetone/H_2O	1222 ± 188	58.1 ± 16.4	0.986	21.0	18.1
**1·HB**	97.5:2.5 Acetone/H_2O	261 ± 16.4	32.0 ± 3.90	0.998	8.15	5.47
**pDEGA-1·HB**	97.5:2.5 Acetone/H_2O	160 ± 10.3	6.35 ± 2.06	0.998	25.1	6.90

S10 References

1. Brynn Hibbert, D.; Thordarson, P., *Chem. Commun.* **2016,** *52* (87), 12792-12805, http://supramolecular.org.
2. Zill, A. T.; Zimmerman, S. C., *Isr. J. Chem.* **2009,** *49* (1), 71-78.
3. Wu, X.; Wu, W.; Cui, X.; Zhao, J.; Wu, M., *J. Mat. Chem. C* **2016,** *4* (14), 2843-2853.
4. Patrick, S. C.; Hein, R.; Docker, A.; Beer, P. D.; Davis, J. J., *Chem. Eur. J.* **2021,** *27* (39), 10201-10209.
